# Supplementary material for: Anderson critical metal phase in trivial states protected by average magnetic crystalline symmetry
Source: Nat Commun. 2024 Apr 9;15:3069. doi: 10.1038/s41467-024-47467-2 (PMC11003978; doi:10.1038/s41467-024-47467-2)
Supplement: Supplementary file 1 — Supplementary Information [file 41467_2024_47467_MOESM1_ESM.pdf]

# Supplementary Information for "Anderson Critical Metal Phase in Trivial States Protected by Average Magnetic Crystalline Symmetry"

Fa-Jie Wang,[1] Zhen-Yu Xiao,[1] Raquel Queiroz,[2] B. Andrei Bernevig,[3] Ady Stern,[4] and Zhi-Da Song[1,5,6,*]

[1]*International Center for Quantum Materials, School of Physics, Peking University, Beijing 100871, China*
[2]*Department of Physics, Columbia University, New York, USA*
[3]*Department of Physics, Princeton University, Princeton, New Jersey 08544, USA*
[4]*Department of Condensed Matter Physics, Weizmann Institute of Science, Rehovot 7610001, Israel*
[5]*Hefei National Laboratory, Hefei 230088, China*
[6]*Collaborative Innovation Center of Quantum Matter, Beijing 100871, China*

(Dated: March 26, 2024)

**Contents**

I. Supplementary numerical data of localization behavior 2
A. Network model on the Manhattan lattice 2
B. Eight-band lattice model $H_{8B}$ 2
C. Simplified eight-band lattice model $H'_{8B}$ 4
D. Local Chern markers of $H_{8B}$ and $H'_{8B}$ 11

II. Critical metal phase in generic magnetic point groups 15
A. Two additional models of CMP 15
B. CMP in generic magnetic point groups 16

III. Network model 20
A. Network model on the Manhattan lattice 20
B. Effective Hamiltonian $H_N$ and band structure of the network model 21
C. Further discussions on the cutoff 28

IV. Mapping the network model to lattice models 31
A. Un-truncated lattice model: $H_N$ on standing wave basis 31
B. Eight-band lattice model $H_{8B}$ 34
C. $H_{8B}$ on corner state basis 36
D. Evolution of the Dirac points 38
E. Simplified eight-band lattice model $H'_{8B}$ 40
F. Disorder potential 45

V. Quasi-1D localization length and Transfer matrix method 47
A. General introduction 47
B. Transfer matrices of the network model on the Manhattan lattice 48
C. Transfer matrices of eight-band lattice models $H_{8B}$ and $H'_{8B}$ 53

References 56

* songzd@pku.edu.cn

[1] We use red characters to indicate figures, equations, and sections in this supplementary material, while green characters indicate those of the main text.

## I. Supplementary numerical data of localization behavior

### A. Network model on the Manhattan lattice

In this subsection, we exhibit the numerical results of the network model defined in Eq. (21) & (22) with random phases $\vartheta_{rand}$ on every chiral edges. Using the transfer matrices defined in Eq. (96) & (97), we can calculate the quasi-1D localization length and conductance. Supplementary Fig. 1 shows the normalized quasi-1D localization length $\Lambda = \rho_{\mathrm{q-1D}}/L$ as a function of scattering angle $\theta \in [-\pi/2, 0]$ with longitudinal size $M = 10^7$ and different transversal sizes $L$. The plot with $\theta \in [0, \pi/2]$ is merely a mirror reflection of it. The critical region is magnified on the right side of Supplementary Fig. 1. As we can see, $\Lambda$ is independent of $L$ when $|\theta| \lesssim \pi/4$, *i.e.*, when the absolute value of transmission amplitude is larger than reflection amplitude. This is indeed what percolation argument predicts: The system will become critical once $(p_1 + p_{-1})/p_0 > 1/2$.

Supplementary Fig. 2 shows the results of conductance $G$ versus $\theta$ with different transversal sizes $L_2$ and longitudinal sizes $L_1 = L_2$ (so that conductance = conductivity). Here, we use $L_1$, $L_2$ instead of $M$, $L$ to emphasis that $G$ is calculated on normal shaped samples rather than quasi-1D samples. The plot with $\theta > 0$ is a mirror reflection of Supplementary Fig. 2. We can see that $G$ is independent of $L_2$ when $|\theta| \lesssim \pi/4$, which is in agreement with Supplementary Fig. 1. It seems that there is an extended phase when $\theta \to 0$. However, the network model belongs to the class A and no extended phase is expected. Also, in contrast with the localized phase, Supplementary Fig. 2 shows that $d \ln G/dL \to 0$ as $L \to \infty$ in the 'extended phase'. Hence, we attribute this 'extended phase' to the finite size effects.

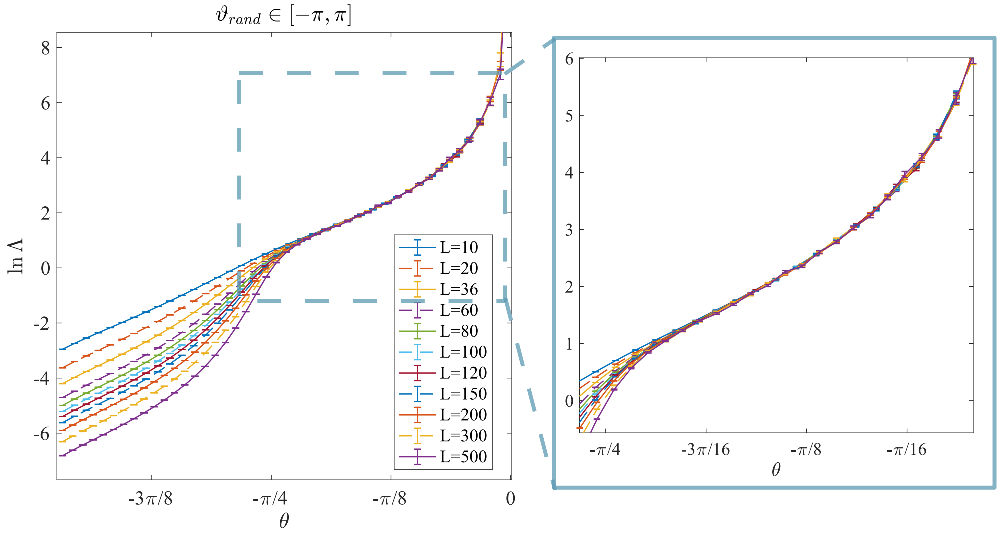


Supplementary Fig. 1. Localization length $\ln \Lambda$ versus scattering angle $\theta$ with different transversal sizes $L$ in the network model defined in Eq. (21) & (22). The quasi-1D direction (longitudinal direction) is indicated in Supplementary Fig. 32(a) and the longitudinal size $M = 10^7$. The data precision $\sigma_\Lambda/\Lambda$ reaches 1% for $\theta < -\pi/8$, 2.5% for $-\pi/8 < \theta < -\pi/16$ and 5% for other data points. $\sigma_\Lambda$ is the unbiased estimation of error mentioned in Supplementary Sec. V A. The right side is the magnified critical region.

### B. Eight-band lattice model $H_{8B}$

We then proceed to the lattice model $H_{8B}$ defined in Eq. (66). Supplementary Fig. 4(a) depicts the phase diagram in parameter space $(W, \tilde{t})$ at $E_F = 0$, where the green region represents the critical metal phase (CMP) and the white region corresponds to localized phase (LP). In LP, the normalized localization length $\Lambda$ decreases as the transversal size $L$ increases, while in CMP, $\Lambda$ remains invariant as $L$ increases. Hence, the scale dependence of $\Lambda(L)$ can be used for determining the phase boundary. Actually, the phase boundary is determined by the p-value of t-statistic of weighted linear regression $1/\Lambda = b_0 + b_1 L$ for given $\tilde{t}$ and $W$. (We offer a heuristic description of t-test and p-value in later paragraphs. Readers can refer to Ref. [1] for more details.) We denote the fitted slope of some data set as $\hat{b}_1$ and the true slope as $b_1$. There should be $\hat{b}_1 > 0$ and $\hat{b}_1 \approx 0$ inside LP and CMP, respectively. Since there is no extended state (inside which $\hat{b}_1 < 0$), we can use $|\hat{b}_1|$ as a criterion. More concretely, we regard the system as localized when the p-value of $|\hat{b}_1|$ is smaller than the commonly used threshold 0.05. That means, if the true slope $b_1 = 0$, we have less than 5% probability to obtain a fitted slope whose absolute value $> |\hat{b}_1|$. Therefore, we should

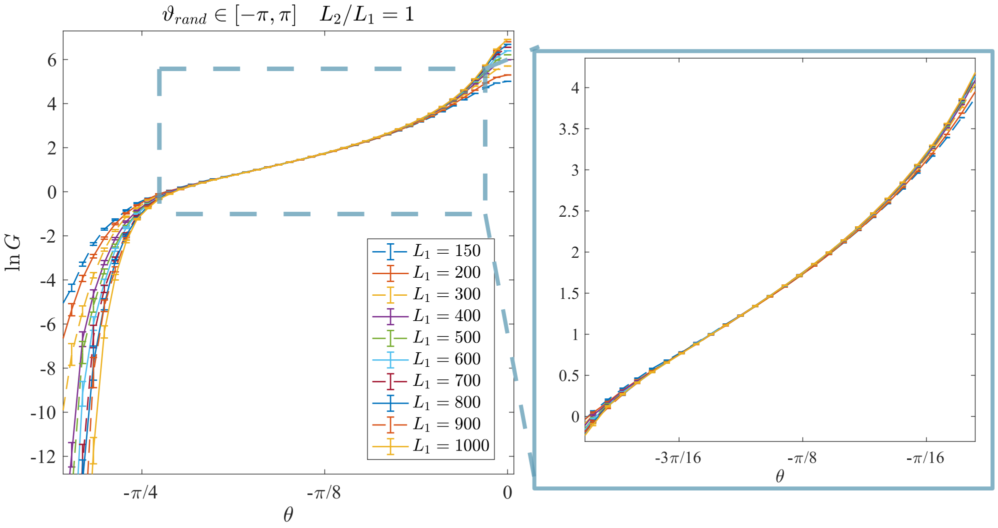


Supplementary Fig. 2. Conductance $\ln G$ versus scattering angle $\theta$ with different transversal sizes $L_2$ in the network model (Eq. (21) & (22)). The sample is normal shaped whose longitudinal length $L_1 = L_2$. And the longitudinal direction is the same as the quasi-1D direction of Supplementary Fig. 32(a). Each data points is averaged over $10^3$ samples and the precision $\sigma_G/G$ reaches 0.5% near the critical region. $\sigma_G$ is the unbiased estimation of error of the conductance average. And the critical region is magnified and shown on the right side.

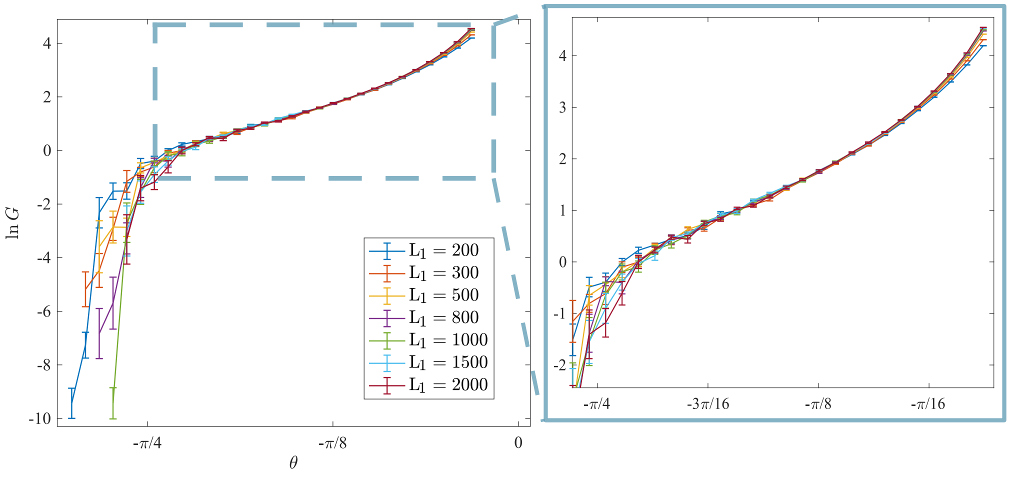


Supplementary Fig. 3. The same to Supplementary Fig. 2 but with larger transversal sizes and 10 samples. The precision reaches 5% near the critical region.

no longer view the system as critical ($b_1 = 0$) and regard it as localized instead. In statistical terminologies, scale dependence of $\Lambda$ is significant at the 5% significance level, so that we reject the null hypothesis. The system is regarded as critical when the p-value is greater than $0.05$.

In this paragraph, we justify the choice of weighted linear regression $1/\Lambda = b_0 + b_1 L$. First, the scale dependence of $\Lambda$ (also $1/\Lambda$) is insignificant near the phase boundary. Hence, no matter what the relation is, we can always take a linear approximation and use linear regression to investigate the scale dependence. Second, estimation techniques of linear regression, *e.g.*, p-value used here, need independently normally distributed data. We derive $\Lambda(L)$ from the smallest Lyapunov exponent $\nu_s = L/\Lambda(L)$, and $\nu_s$ is obtained from Eq. (94) so that only $1/\Lambda \propto \nu_s$ is normally distributed. Thus, we choose $1/\Lambda$ rather than $\Lambda$ to do the linear regression. Third, the variance of $1/\Lambda$ varies with $L$. Therefore, we should use the least square method with weights $\{1/\sigma_i^2 | i = 1, 2, \cdots, n\}$, where $\{\sigma_i^2\}$ is the variance of corresponding data points $\{1/\Lambda(L_i)\}$. Last, we take the data with $L \geq 50$ to avoid the finite size effects, which will result in an underestimation of CMP in our case.

In the following paragraphs, we present a brief introduction of t-test based on the p-value. Recall that the unknown true values of regression slope and intercept are $b_1$ and $b_0$, respectively. The fitted slope and intercept from some data set $\{1/\Lambda(L_i) | i \in$

$[1, n]\}$ are $\hat{b}_1$ and $\hat{b}_0$, respectively. As mentioned in the last paragraph, each data point satisfies $1/\Lambda(L_i) = 1/\tilde{\Lambda}(L_i) + \delta(1/\Lambda(L_i))$, where $\tilde{\Lambda}(L_i)$ is the unknown true value of $\Lambda(L_i)$ and $\delta(1/\Lambda(L_i))$ is a normally distributed random variable with standard error $\sigma_i$. The unbiased estimation of $\sigma_i$ has been described in Supplementary Sec. V A. Each of the transversal sizes $\{L_i\}$ certainly has no error, nevertheless we can take an average of them $\bar{L} = \frac{1}{n}\sum_{i=1}^{n} L_i$. Then, we point out without proof (see Chapter 4.5 of [2] for a proof) that the unbiased estimation of the variance of $\hat{b}_1$ is given by

$$\hat{\sigma}_{b_1}^2 = \frac{\frac{1}{n-2}\sum_{i=1}^{n}(\frac{1}{\Lambda(L_i)} - \hat{b}_0 - \hat{b}_1 L_i)^2/\sigma_i^2}{\sum_{i=1}^{n}(L_i - \bar{L})^2/\sigma_i^2} . \tag{1}$$

The factor $\frac{1}{n-2}$ represents that the statistical degrees of freedom is $n-2$. Then, the following random variable satisfies the Student's t-distribution with $n-2$ degrees of freedom (denoted as $t_{n-2}$):

$$\Delta_1 = \frac{\hat{b}_1 - b_1}{\hat{\sigma}_{b_1}} \sim t_{n-2} . \tag{2}$$

We omit the concrete formula of $t_{n-2}$. We merely point out that, similar to the normal distribution, the curve of probability density of Student's t-distribution is symmetric and bell-shaped around zero but with heavier tails. As the number of degrees of freedom $(n-2) \to \infty$, $t_{n-2}$ approaches the normal distribution with mean 0 and variance 1. Now, we can define the p-value as the probability of event $|\Delta_1| > C \geq 0$ and denote it as $P(C)$. Since $P(C)$ decreases as $C$ increases, it is unlikely to obtain a large $\hat{b}_1/\hat{\sigma}_{b_1}$ when $b_1 = 0$. If the regression results in a large $\hat{b}_1/\hat{\sigma}_{b_1}$, we can reject the hypothesis that $b_1 = 0$ ($\Lambda$ is independent of $L$ and the system is critical). As a consequence, we should accept that the system is localized. In our case, we choose 0.05 as the threshold, *i.e.*, the system is regarded as localized when $P(|\hat{b}_1/\hat{\sigma}_b|) \leq 5\%$ and otherwise regarded as critical. The above procedure is called a t-test (based on p-value) with threshold 0.05, where $t$ represents the Student's t-distribution.

The numerical results of $H_{8B}$ are shown in Supplementary Fig. 4. Supplementary Fig. 4(a) illustrate the phase diagram of $H_{8B}$ in the space of $W - \tilde{t}$ ($E_F = 0$). The phase boundary close to the clean limit ($W < v/a$) is hard to determine since the localization lengths have large relative errors. Hence, we focus on $W > v/a$. From Supplementary Fig. 4(a) and Supplementary Fig. 22, we can see that CMP roughly arises inside the metallic region in the clean limit (about $1 < a\tilde{t}/v < 2.5$). And the bandwidth (of all bands) of $H_{8B}$ in the metallic region is about $8v/a$, which is comparable to the maximal disorder strength reached by CMP ($5v/a$). Supplementary Fig. 4(b) and 4(c) show $\Lambda$ as functions of $\tilde{t}$ and $W$, respectively. The parameters used in these plots are indicated by the red lines in Fig, 4(a). As we can see, $\Lambda$ is indeed independent of $L$ in CMP. During the growth of $\tilde{t}$, the transition LP-CMP-LP (Supplementary Fig. 4(b)) is in accord with the network model (Supplementary Fig. 1 and its mirror reflection for $\theta > 0$). We also calculate $\ln\Lambda$ vs. $\tilde{t}$ in LP, and the results are shown in Supplementary Fig. 6. As expected, $\Lambda$ always decreases as $L$ increases.

As explained in Supplementary Sec. V A, we need further validations of the CMP in $H_{8B}$, since the localization length is fairly large in CMP: $\rho_{\text{q}-1\text{D}} = e^{3\sim4}L \approx 20 \sim 50L$. To rule out this problem, we take two points in CMP and calculate $\Lambda(L)$ up to $L_{\max} = 500$. The results are shown in Supplementary Fig. 8(a) & 8(b), and no significant scale dependence of $\Lambda$ is found. As explained in Supplementary Sec. V A, we have two more evidences that validate the CMP in $H_{8B}$: CMP does not shrink as $L$ increases (Supplementary Fig. 8(b)) and the local Chern markers highly fluctuate in CMP (Supplementary Fig. 11(a)). In addition, to make sure that the choice $E_F = 0$ is not special, we calculate $\Lambda$ versus $\tilde{t}$ with $E_F = -\pi v/10a$ (Supplementary Fig. 9), which still shows the existence of CMP.

### C. Simplified eight-band lattice model $H'_{8B}$

The results of $H'_{8B}$ (Eq. (85)) are shown in Supplementary Fig. 5 and Supplementary Fig. 7. Supplementary Fig. 5(a) shows the phase diagram in $\tilde{t} - W$ plane ($E_F = 0$), and the phase boundary is determined in the same way as $H_{8B}$. Supplementary Fig. 5(b) & 5(c) show the localization length along paths indicated by the red lines in Supplementary Fig. 5(a). The same to $H_{8B}$, the transition pattern along the $\tilde{t}$ axis is LP-CMP-LP (Supplementary Fig. 5(b)). Supplementary Fig. 7 shows $\ln\Lambda - \tilde{t}$ in LP, and, as expected, $\Lambda$ decreases as $L$ increases.

Combining Supplementary Fig. 5(a) with Supplementary Fig. 28, we see that CMP can arises inside the semi-metal region ($2a\tilde{t}/\pi v \in [1, A = 1.2]$) where the four Dirac cones in Supplementary Fig. 28 exist. And CMP of $H'_{8B}$ persists to a disorder strength ($\approx 3v/a$) that is comparable to the bandwidth of the two bands in Supplementary Fig. 28 ($\approx 3v/a$). Numerical calculations on other $A$ show that CMP centers at $2a\tilde{t}/\pi v = \sqrt{A}$ and extends most to around $\pi v/2a \sim A\pi v/2a$. It is trivial that CMP cannot arise away from the semi-metal region, since the band structure is gapped. But it is nontrivial that, for moderate disorder strength $W < 1.5v/a$, CMP can approximately fill up (in terms of $\tilde{t}$) the entire semi-metal region ($a\tilde{t}/v = \pi/2 \sim 0.6\pi$). Therefore, the existence of the four Dirac cones in Supplementary Fig. 28, *i.e.*, the transition region between the two OAI's, must have a tight connection with the critical metal phase. Also, considering that we offer a quantitative mapping from the network

model to $H'_{8B}$ in Supplementary Sec. IV, there must be a connection between the OAI transition and the conductive percolation state.

Again, the phase boundary close to the clean limit ($W < v/a$) is hard to determine. Besides the large error in $\Lambda$ as in $H_{8B}$, here we have another problem when $W$ is small: fluctuation in $DOS_F$ (the density of states at the Fermi level). As we know, the Bloch wave vector is discrete for a finite system. In our case, although the longitudinal size $M$ can reach $10^7$, the transversal size $L$ will not exceed the order of $10^2$. See Supplementary Fig. 28, due to the coarse quantization of the transversal momentum component, only a (clean and $E_F = 0$) system with fine-tuned $\tilde{t}$ & $L$ can 'see' the Dirac cones; otherwise, the system will become a gapped insulator. Since $\tilde{t}$ affects the position of the Dirac cones, when the system is clean enough, $\Lambda$ will fluctuate as $\tilde{t}$ varies in the semi-metal region. Note that $DOS_F$ is also sensitive to $\tilde{t}$ and $L$ when $E_F \neq 0$, hence the fluctuation of $\Lambda$ also exist in these cases. The weaker $W$ is, the larger $L$ is needed to erase the fluctuation. This makes it expensive to investigate the phase boundary near the clean limit. In practice, we ignore the data with $L < 50$ for the determination of phase boundary to avoid the finite size effect.

We also executed the examination of scale dependence of $\Lambda$ up to $L_{\max} = 500$. The results are shown in Supplementary Fig. 8(c) & 8(d) and no significant scale dependence can be found. In addition, to make sure that the choice $E_F = 0$ is not special, we calculate $\Lambda$ versus $\tilde{t}$ with $E_F = -\pi v/10a$ (Supplementary Fig. 10), which still shows the existence of CMP.

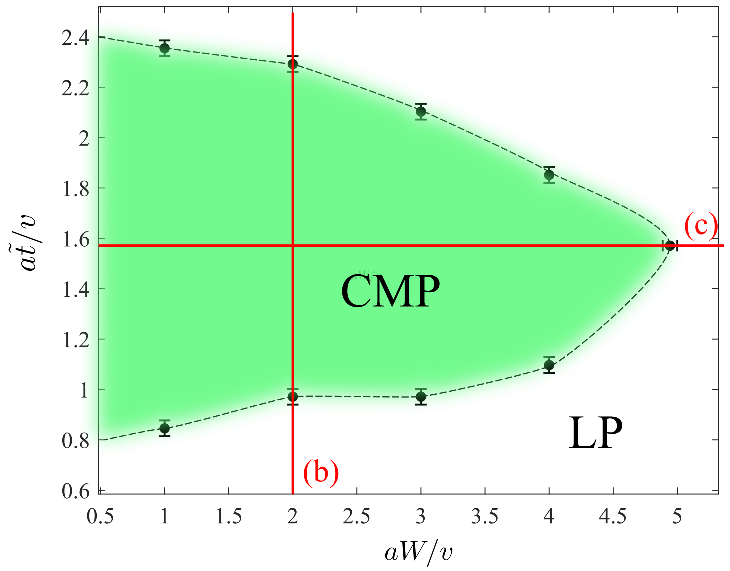


(a) Phase diagram of $H_{8B}$ with $E_F = 0$ in the parameter space spanned by $W, \tilde{t}$

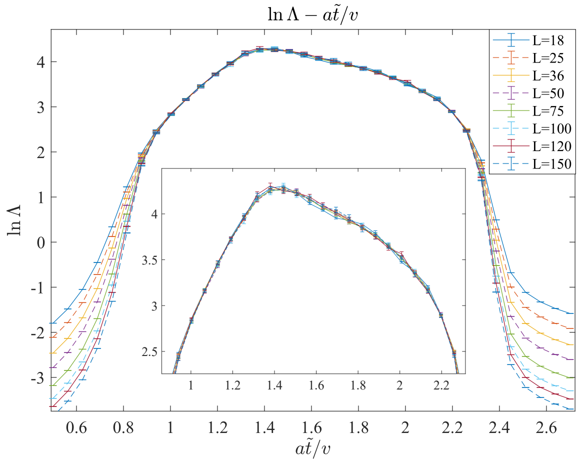


(b) $\ln \Lambda$ versus $\tilde{t}$ with $W = 2v/a$

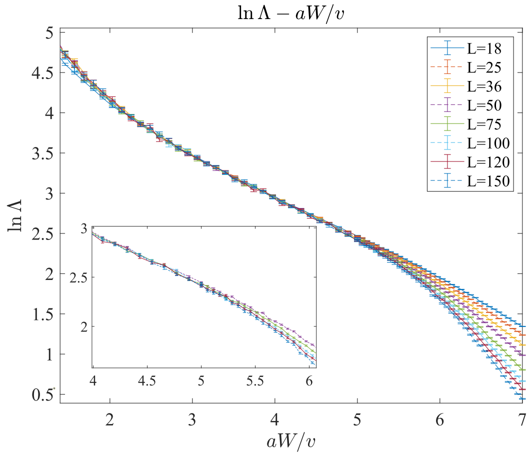


(c) $\ln \Lambda$ versus $W$ with $\tilde{t} = \pi v/2a$

Supplementary Fig. 4. Phase diagram and localization lengths of lattice model $H_{8B}$ (Eq. (66)) with $E_F = 0$. (a) The phase diagram in $\tilde{t} - W$ plane. The phase boundary is determined by the p-value of weighted linear regression of $1/\Lambda - L$ for given $\tilde{t}, W$ and $L \geq 50$. The regression weight of a data point with variance $\sigma^2$ is proportional to $1/\sigma^2$. We regard the system as localized if the p-value of fitted slope is less than $0.05$, *i.e.*, the positive fitted slope is significant at the 5% significance level. Otherwise, we regarded it as critical. (See Supplementary Sec. I B for more details) The green region in (a) indicates the critical metal phase enclosed by the localized phase. And the red lines in (a) represent the parameters used in (b) and (c), respectively. (b) Localization length as the function of $\tilde{t}$ with $W = 2v/a$ and various transversal sizes $L$. The longitudinal size $M = 10^7$ and the data precision $(\sigma_\Lambda/\Lambda)$ reaches 3%. The inset in (b) is a zoomed plot of the critical region. (c) Localization length as the function of $W$ with $\tilde{t} = \pi v/2a$, where the inset magnifies the transition zone between the critical and localized regions (we ignore the curves of $L = 18 \sim 36$ in the inset to avoid the finite size effects). The longitudinal size and data precision are the same as (b).

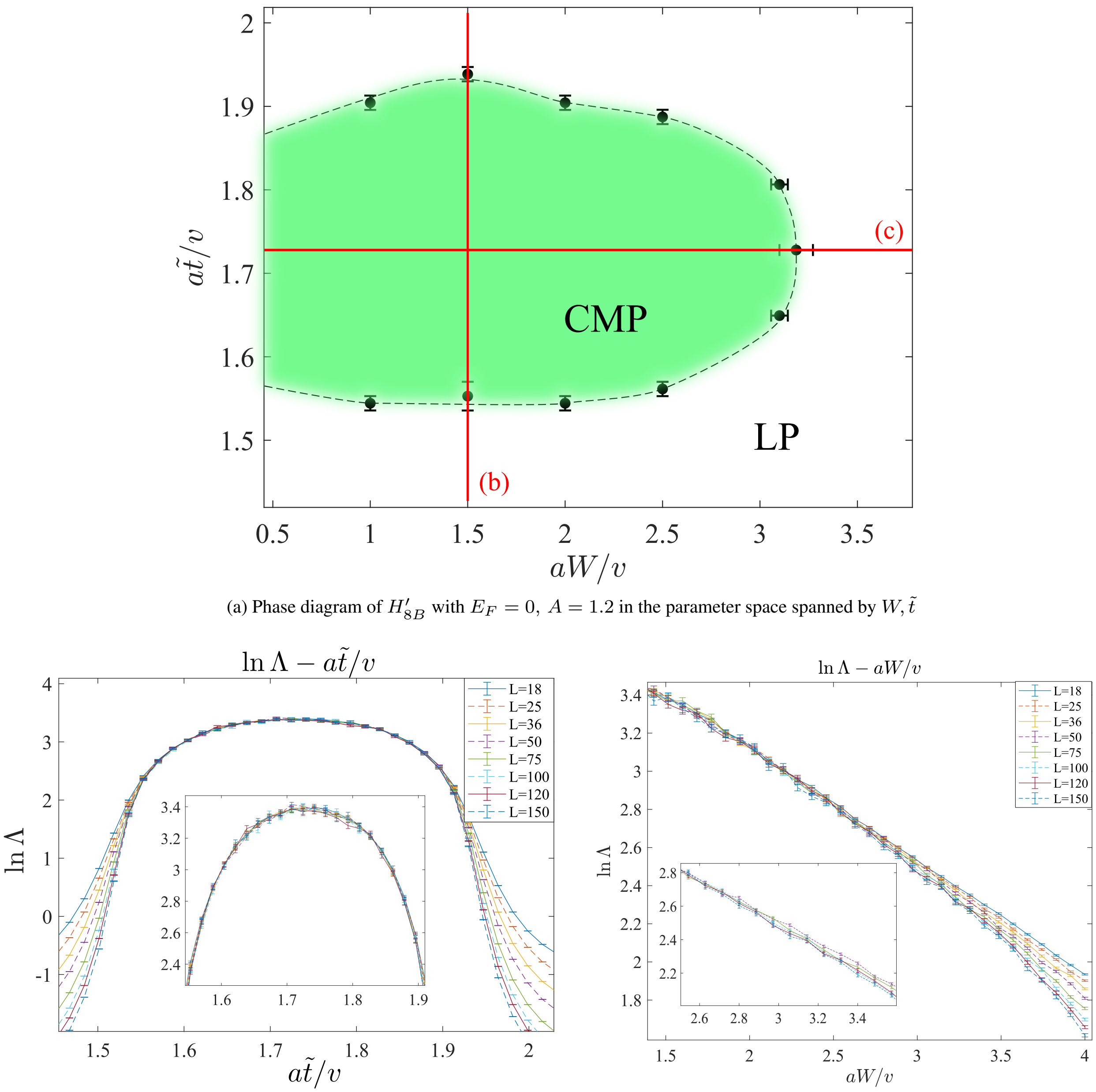


(a) Phase diagram of $H'_{8B}$ with $E_F = 0,\ A = 1.2$ in the parameter space spanned by $W, \tilde{t}$

(b) $\ln\Lambda$ versus $\tilde{t}$ with $W = 1.5v/a$

(c) $\ln\Lambda$ versus $W$ with $\tilde{t} = 1.1\pi v/2a$

Supplementary Fig. 5. Phase diagram and localization length of $H'_{8B}$ (Eq. (85)) with $E_F = 0$ and $A = 1.2$. (a) The phase diagram in $\tilde{t} - W$ plane. The phase boundary is determined in the same way as Supplementary Fig. 4. The green region and red lines also have the same meanings as that of Supplementary Fig. 4. (b) Localization length as the function of $\tilde{t}$ with $W = 1.5v/a$ and various transversal sizes. The longitudinal size $M = 10^7$ and the data precision $(\sigma_\Lambda/\Lambda)$ reaches 2%. The inset in (b) is a zoomed plot of the critical region. (c) Localization length as the function of $W$ with $\tilde{t} = 1.1\pi v/2a$, where the inset magnifies the transition zone between the critical and localized regions (we ignore the curves of $L = 18 \sim 36$ in the inset to avoid the finite size effects). The longitudinal size and data precision are the same as (b).

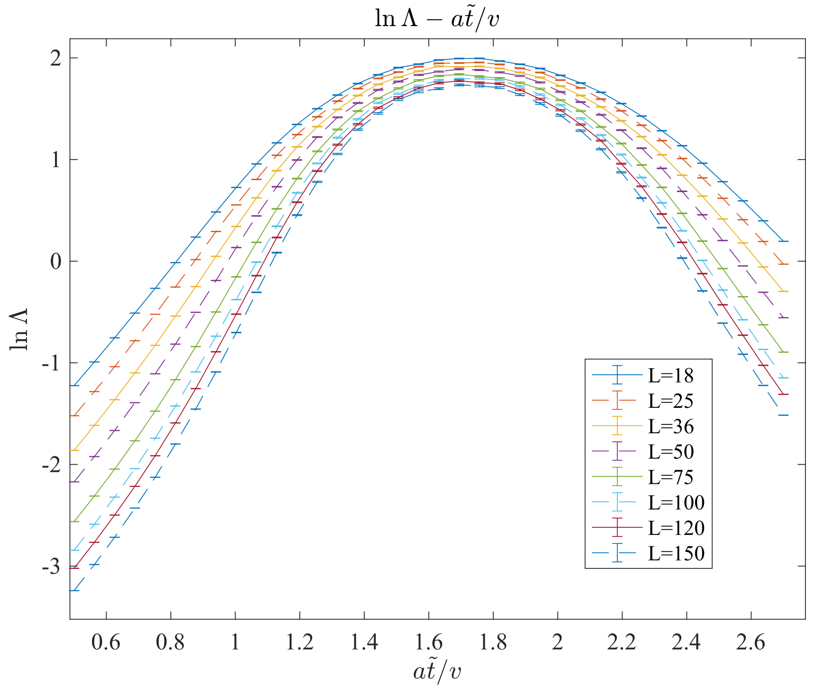


Supplementary Fig. 6. Localization length $\Lambda$ as the function of $\tilde{t}$ of $H_{8B}$ (Eq. (66)) with $W = 6v/a$ (inside the localized phase) and $E_F = 0$. The longitudinal size $M = 10^7$ and the data precision $(\sigma_\Lambda/\Lambda)$ reaches $1\%$. As expected for the localized phase, $\Lambda$ decreases as the transversal size $L$ increases.

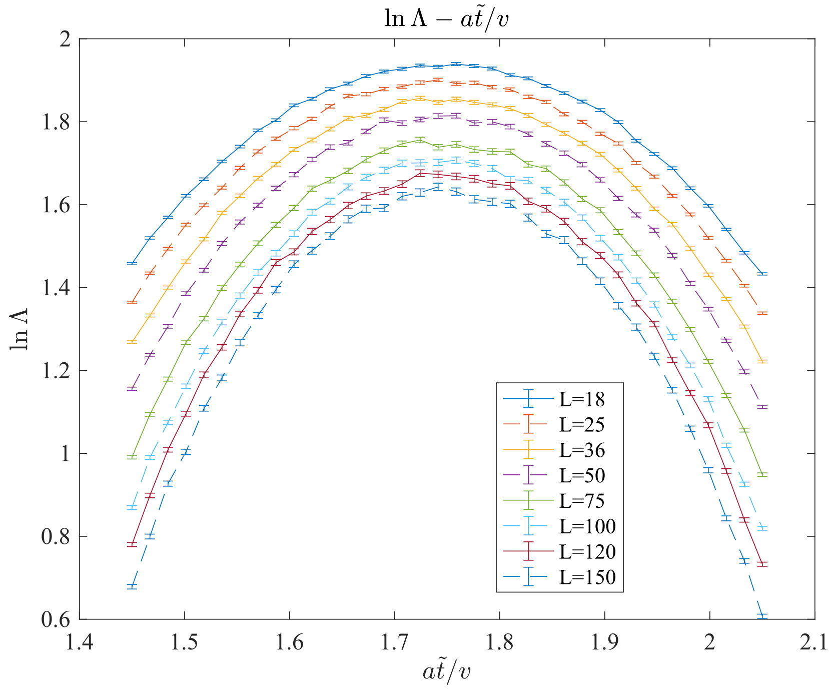


Supplementary Fig. 7. Localization length $\Lambda$ as the function of $\tilde{t}$ of $H'_{8B}$ (Eq. (85)) with $A = 1.2$, $W = 4v/a$ (inside the localized phase) and $E_F = 0$. The longitudinal size $M = 10^7$ and the data precision $(\sigma_\Lambda/\Lambda)$ reaches $0.9\%$. As expected for the localized phase, $\Lambda$ decreases as the transversal size $L$ increases.

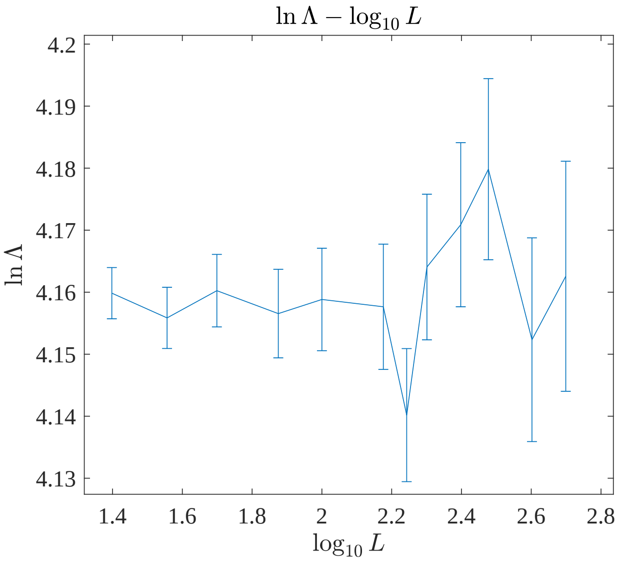


(a) $\tilde{t} = \pi v/2a$, $W = 2v/a$ in $H_{8B}$

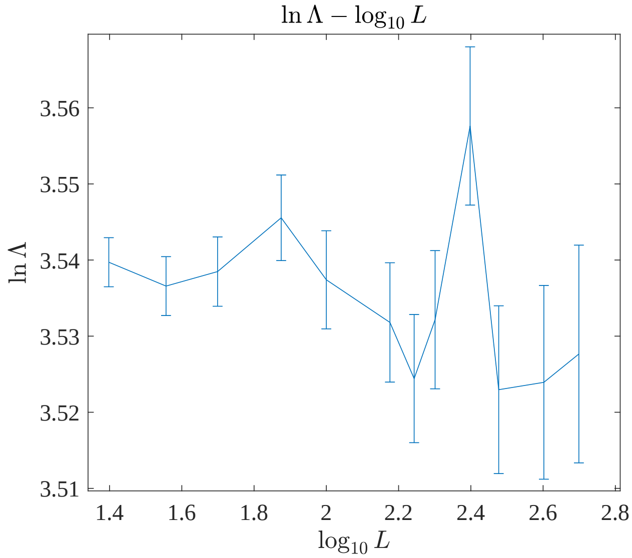


(b) $\tilde{t} = 2v/a$, $W = 2v/a$ in $H_{8B}$

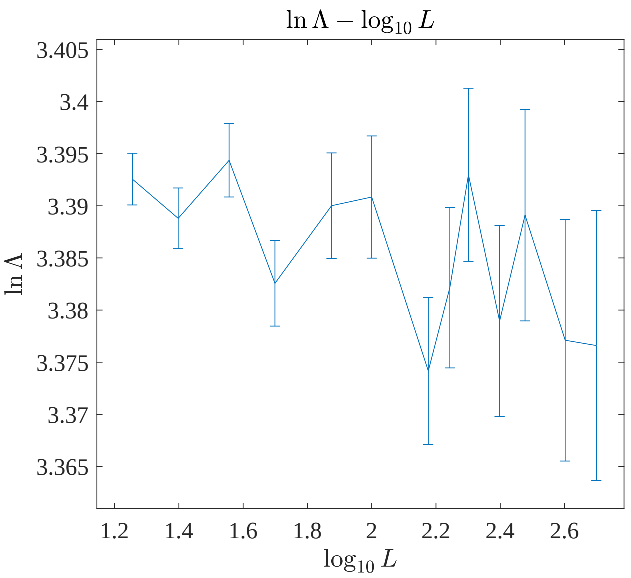


(c) $A = 1.2$, $\tilde{t} = 1.1\pi v/2a$, $W = 1.5v/a$ in $H'_{8B}$

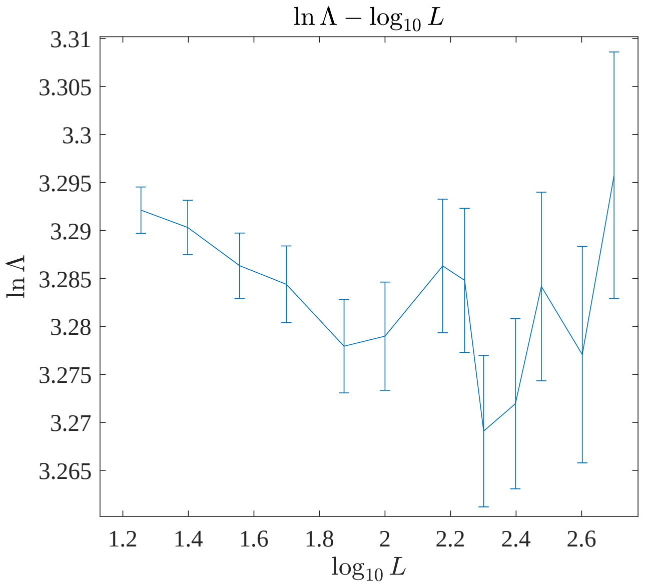


(d) $A = 1.2$, $\tilde{t} = 1.15\pi v/2a$, $W = 1.5v/a$ in $H'_{8B}$

Supplementary Fig. 8. The normalized localization length $\Lambda$ versus transversal size $L$ in CMPs of $H_{8B}$ ((Eq. (66))) and $H'_{8B}$ (Eq. (85)). (a), (b) $\Lambda$ as the function of $L$ for $H_{8B}$ with $\tilde{t} = \pi v/2a$, $W = 2v/a$ and $\tilde{t} = 2v/a$, $W = 2v/a$, respectively. (c), (d) $\Lambda$ as the function of $L$ for $H'_{8B}$ with $A = 1.2$, $W = 1.5v/a$, $\tilde{t} = 1.1\pi v/2a$ and $A = 1.2$, $W = 1.5v/a$, $\tilde{t} = 1.15\pi v/2a$, respectively. For all these four plots, the maximum transversal size reaches $L_{\max} = 500$, the longitudinal size $M = 10^8$ and the data precision $(\sigma_\Lambda/\Lambda)$ reaches 1%. None of these plots shows a significant decline of $\ln\Lambda$ when $L \to \infty$, which indicates that the scale independence of $\Lambda$ in the CMPs in Supplementary Fig. 4(a) and Supplementary Fig. 5(a) is not due to finite size effects. Therefore, this figure support the existence of CMPs in $H_{8B}$ and $H'_{8B}$.

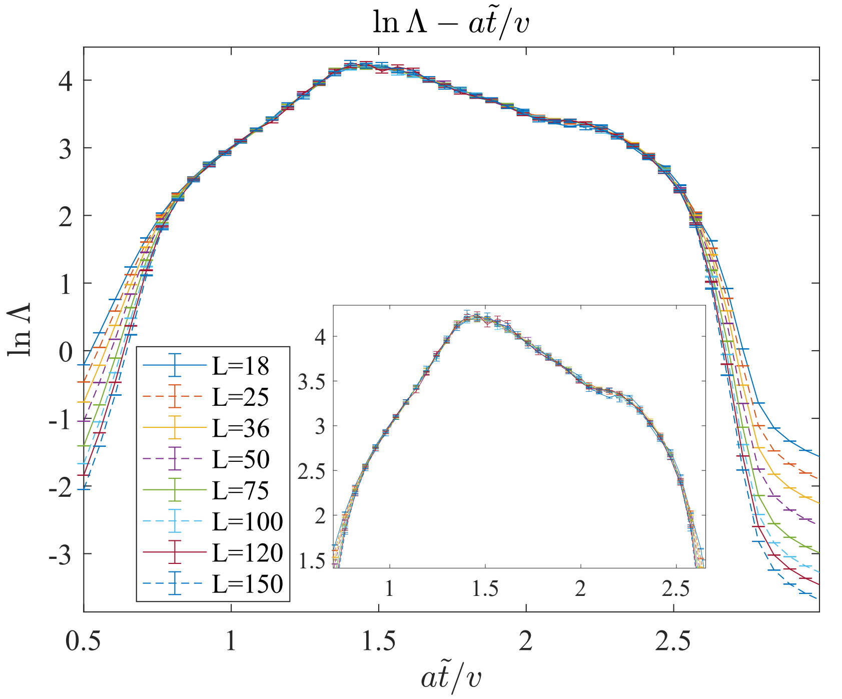


Supplementary Fig. 9. Localization length $\Lambda$ as the function of $\tilde{t}$ of $H_{8B}$ (Eq. (66)) with $W = 2v/a$ and $E_F = -\pi v/10a$. The longitudinal size $M = 10^7$ and the data precision $(\sigma_\Lambda/\Lambda)$ reaches $3\%$. The middle region in this figure is a critical phase, since $\Lambda$ remains invariant as $L \to \infty$. The inset shows the zoomed critical region. This figure shows the existence of CMP in $H_{8B}$ for $E_F \neq 0$. Thus, the CMP in $H_{8B}$ does not rely on the special choice $E_F = 0$.

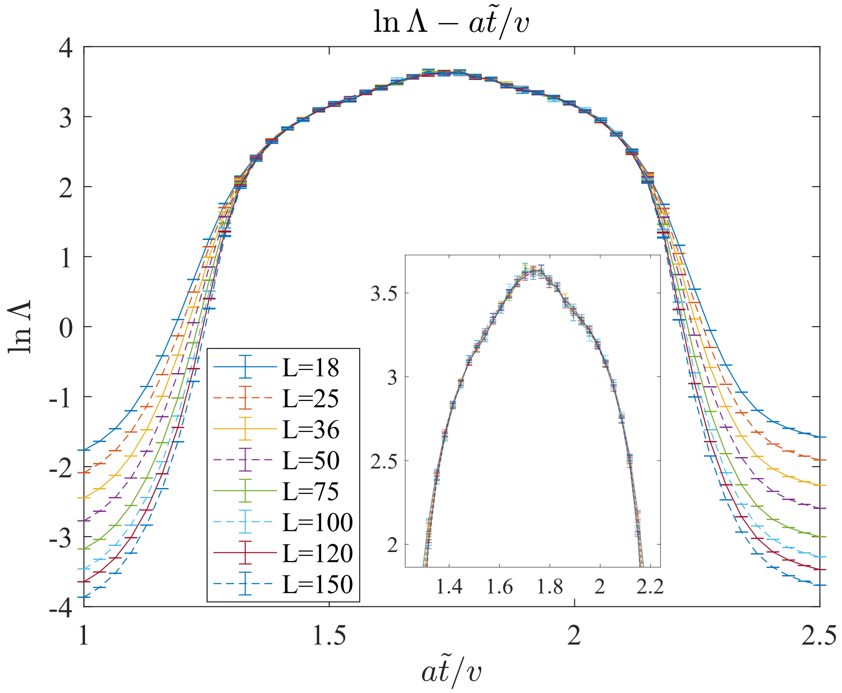


Supplementary Fig. 10. Localization length $\Lambda$ as the function of $\tilde{t}$ of $H'_{8B}$ (Eq. (85)) with $A = 1.2$, $W = 1.5v/a$ and $E_F = -\pi v/10a$. The longitudinal size $M = 10^7$ and the data precision $(\sigma_\Lambda/\Lambda)$ reaches $3\%$. The middle region in this figure is a critical phase, since $\Lambda$ remains invariant as $L \to \infty$. The inset shows the zoomed critical region. This figure shows the existence of CMP in $H'_{8B}$ for $E_F \neq 0$. Thus, the CMP in $H'_{8B}$ does not rely on the special choice $E_F = 0$ and the *averaged* chiral symmetry at $E_F = 0$ (see the last but one paragraph of Supplementary Sec. IV E).

### D. Local Chern markers of $H_{8B}$ and $H'_{8B}$

In Supplementary Sec. I, we show that the network model (Eq. (21) & (22)), $H_{8B}$ (Eq. (66)), and $H'_{8B}$ (Eq. (85)) all have critical metal phases. Recall that the CMP in network model represents a conductive percolation system (Fig. 1(b)), and the CMPs in $H_{8B}$ and $H'_{8B}$ correspond to transitions between trivial OAIs (Supplementary Sec. IV B & IV E). Also, in Supplementary Sec. IV, we show a quantitative mapping from the network model to $H_{8B}$ and $H'_{8B}$. Then, it is natural to ask whether the CMPs in $H_{8B}$ and $H'_{8B}$ represent conductive percolation systems. Especially for $H'_{8B}$, the answer is far from obvious since the band structure of $H'_{8B}$ near the Fermi level $E_F = 0$ (Supplementary Fig. 28) significantly differs from that of the network model with $E_F = \pi v/4a$ (Supplementary Fig. 18 & 23). Fortunately, the local Chern marker (LCM) enables us to investigate this question directly.

Refs. [3, 4] showed that the Chern number of a gapped lattice Hamiltonian can be computed from the Bott index as

$$C = \frac{1}{2\pi}\mathrm{Im}\left[\mathrm{Tr}\ln(\Phi_x\Phi_y\Phi_x^\dagger\Phi_y^\dagger)\right] \tag{3}$$

where

$$\Phi_x = \hat{P}\exp(i\frac{2\pi}{L}\hat{x})\hat{P}, \qquad \Phi_y = \hat{P}\exp(i\frac{2\pi}{L}\hat{y})\hat{P} \tag{4}$$

$\hat{x}, \hat{y}$ are position operators, $P$ the projector to the occupied states, and $L$ the system size. Eq. (3) applies to either clean or disordered gapped systems subject to the periodic boundary condition. When $L \to \infty$, Eq. (3) reduces to

$$C = \frac{1}{2\pi i A_{\mathcal{B}}}\sum_{\vec{R}\alpha\in\mathcal{B}}\left\langle \vec{R}\alpha\right|\left[\hat{P}\hat{x}\hat{P}, \hat{P}\hat{y}\hat{P}\right]\left|\vec{R}\alpha\right\rangle = \frac{1}{\pi A_{\mathcal{B}}}\,\mathrm{Im}\sum_{\vec{R}\alpha\in\mathcal{B}}\left\langle \vec{R}\alpha\right|\hat{P}\hat{x}\hat{P}\hat{y}\hat{P}\left|\vec{R}\alpha\right\rangle \tag{5}$$

where $\vec{R}$ is the position vector of primitive cell and $\alpha$ indicates the orbitals in one primitive cell. $\mathcal{B}$ is a large but finite region where $x/L, y/L \ll 1$, $A_{\mathcal{B}}$ is the area of this region. In the second equation of Eq. (5), we use the fact:

$$\left\langle \vec{R}\alpha\right|\hat{P}\hat{x}\hat{P}\hat{y}\hat{P}\left|\vec{R}\alpha\right\rangle = \left(\left\langle \vec{R}\alpha\right|\hat{P}\hat{y}\hat{P}\hat{x}\hat{P}\left|\vec{R}\alpha\right\rangle\right)^* \tag{6}$$

where $(...)^*$ is the complex conjugate. We can consider every term in the summation as "local Chern number of site $(\vec{R}, \alpha)$", *i.e.*, we define LCM (for a 2D system) as [5]

$$C(\vec{R}, \alpha) = \frac{4\pi n_c}{A_c}\mathrm{Im}\left\langle \vec{R}\alpha\right|\hat{P}\hat{x}\hat{P}\hat{y}\hat{P}\left|\vec{R}\alpha\right\rangle \tag{7}$$

where $A_c$ is the area of one primitive cell and $n_c$ is the number of orbitals per primitive cell. One can check that the Chern number Eq. (5) can be regarded as the average of LCM Eq. (7). Alternatively, one can use

$$C(\vec{R}) = \frac{4\pi}{A_c}\sum_\alpha \mathrm{Im}\left\langle \vec{R}\alpha\right|\hat{P}\hat{x}\hat{P}\hat{y}\hat{P}\left|\vec{R}\alpha\right\rangle \tag{8}$$

as the "local Chern number of cell $\vec{R}$". From now on, LCM stands for the local Chern marker of one cell $C(\vec{R})$, unless otherwise stated.

Eq. (5) can be applied to systems subject to open boundary conditions as long as the region $\mathcal{B}$ is well separated from the boundary such that $\mathcal{B}$ is well-gapped. Otherwise, the yielded $C$ will not reflect the bulk Chern number. For example, if we take $\mathcal{B}$ as the whole system including the boundaries, Eq. (5) would become zero because

$$\sum_{\vec{R}\alpha}\left\langle \vec{R}\alpha\right|\hat{P}\hat{x}\hat{P}\hat{y}\hat{P}\left|\vec{R}\alpha\right\rangle = \sum_{\vec{R}\alpha}\left\langle \vec{R}\alpha\right|\hat{P}\hat{y}\hat{P}\hat{x}\hat{P}\left|\vec{R}\alpha\right\rangle \tag{9}$$

due to the cyclic property of the trace operation. That means, for a $L \times L$ sample with bulk Chern number $C$, the LCMs near the boundary will diverge in the order of $\mathcal{O}(C \cdot L)$ to cancel the summation of the bulk LCMs. More generally, if the sample comprises several macroscopically (comparable to the sample size) homogeneous regions with different Chern numbers, LCMs deep inside each region still converge to the corresponding Chern number. And near the boundaries of different regions, LCMs fluctuate around the mean value of the corresponding Chern numbers. Therefore, Eqs. (7) and (8) are useful local indicators of topological properties.

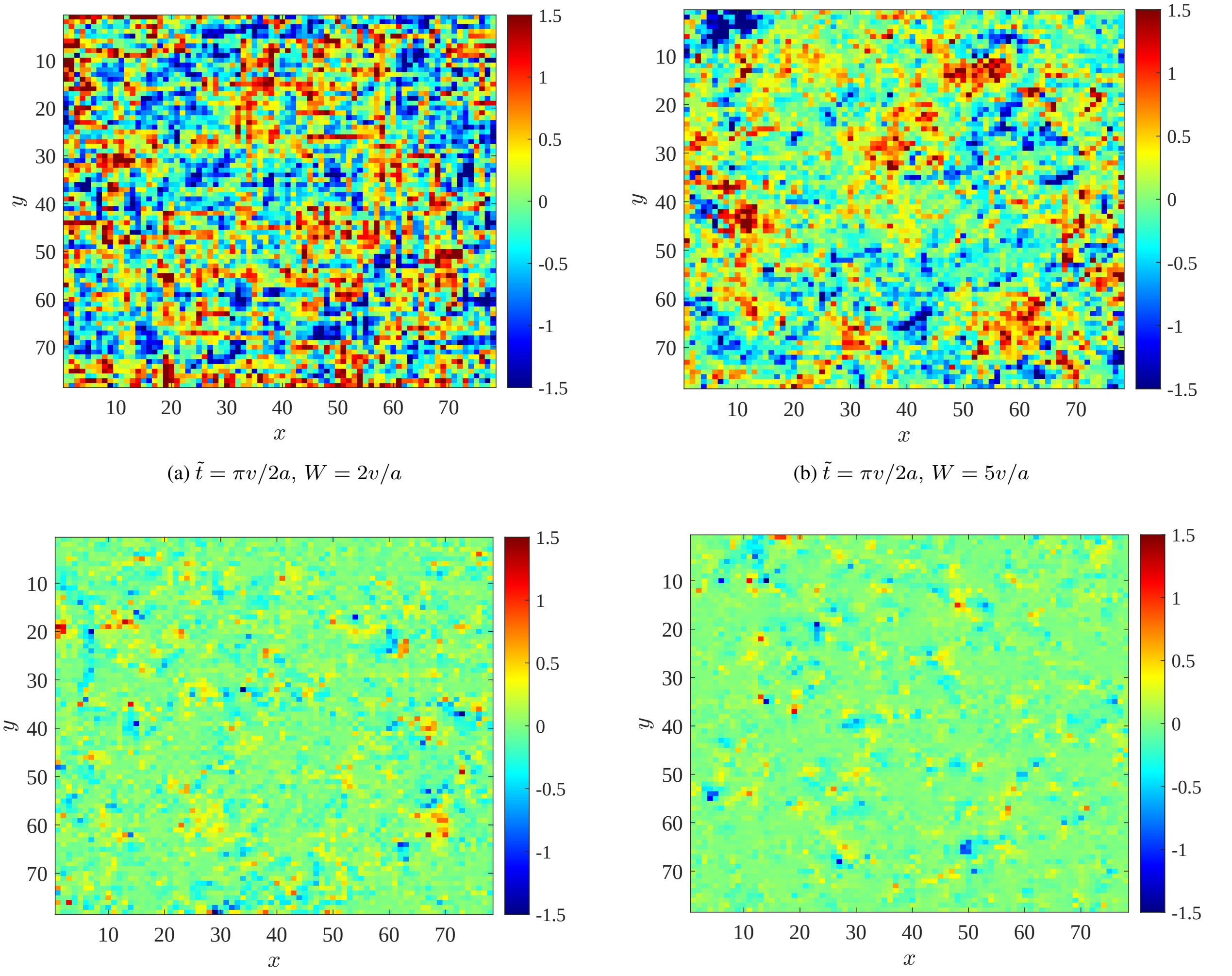


(a) $\tilde{t} = \pi v/2a,\ W = 2v/a$

(b) $\tilde{t} = \pi v/2a,\ W = 5v/a$

(c) $\tilde{t} = \pi v/2a,\ W = 7v/a$

(d) $\tilde{t} = 0.8v/a,\ W = 2v/a$

Supplementary Fig. 11. LCMs of $H_{8B}$ in given disorder configurations at $E_F = 0$ with $80 \times 80$ cells. Each pixel in the color maps corresponds to one cell. The open boundary condition is applied. LCMs with $|C(\vec{R})| > 1.5$ will be mapped to $\pm 1.5$ in the color maps. For (a) $\sim$ (b), $\tilde{t}$ is fixed on $\pi v/2a$ and $aW/v = 2,\ 5,\ 7$, respectively. (d) LCM with $\tilde{t} = 0.8v/a$ and $W = 2v/a$.

With respect to our lattice models, since the total Chern number is enforced to be zero by the averaged $C_{2z}T$ symmetry, no boundary singularity of LCM will present. If the percolation argument is true, the profile of Chern numbers will be mottled in CMP, and no macroscopically homogeneous region is guaranteed. So we expect that LCMs will strongly fluctuate in CMP.

This is indeed the case for $H_{8B}$. As shown in Supplementary Fig. 11(a)$\sim$ 11(c), the fluctuation of LCM fades as $W$ increases and drives the system into LP. Supplementary Fig. 11(d) shows that localizing the system by tuning $\tilde{t}$ will also fade the fluctuation. Therefore, LCMs indeed form a staggered pattern in CMP, which disappears once the system is localized. We also calculate the statistical distribution of $\left\langle C^2(\vec{R}) \right\rangle^{1/2}$ which reflects the ratio of regions with nontrivial Chern number. Here, $\langle \cdots \rangle$ means averaging over all the primitive cells. The results are shown in Supplementary Fig. 13(a)$\sim$13(b). As we can see, for all the configurations, $\left\langle C^2(\vec{R}) \right\rangle^{1/2}$ exceeds 0.5 in CMP and diminishes toward zero in LP. That means the nontrivial Chern blocks dominate ($p_1 + p_{-1} > 0.5$) in CMP and the trivial blocks dominate ($p_1 + p_{-1} < 0.5$) in LP. From these observations, we conclude that the percolation argument is indeed valid for $H_{8B}$.

The results of $H'_{8B}$ are shown in Supplementary Fig. 12 and Supplementary Fig. 13(c)$\sim$13(d). As shown by Supplementary Fig. 12(a)$\sim$ 12(c), the fluctuation of LCM fades as $W$ increases and drives the system into LP. Supplementary Fig. 12(d) shows that localizing the system by tuning $\tilde{t}$ will also fade the fluctuation. The statistical results is also similar to that of $H_{8B}$, $\left\langle C^2(\vec{R}) \right\rangle^{1/2} > 1/2$ in CMP and diminishes toward zero in LP. So one can conclude that the percolation argument is also valid

for $H'_{8B}$.

Before the end of this section, we can make one more comment about our LCM data. These profiles of LCM confirm our claim about CMP in an independent way from the localization length. Since electrons can propagate along the edges of Chern blocks, a sample dominated by staggered $C = \pm 1$ Chern blocks (and the ratios of $C = 1, -1$ regions are equal) can not be localized. On the other hand, the numerical results of the localization length (Supplementary Fig. 4 & Supplementary Fig. 5) show that there is no metallic phase ($\beta = \frac{\mathrm{d} ln g}{\mathrm{d} \ln L} > 0$) . Hence, the only choice is to be critical.

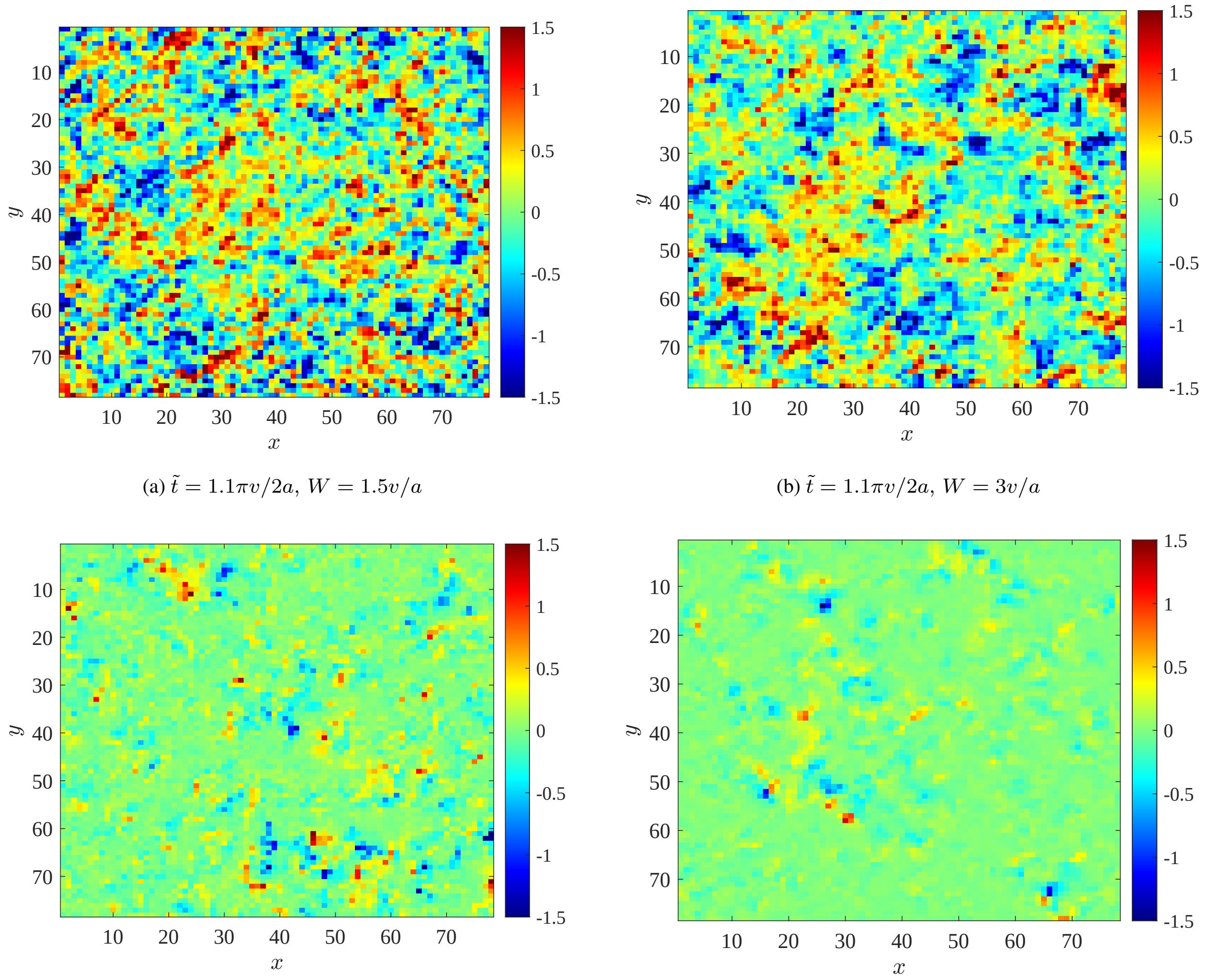


(a) $\tilde{t} = 1.1\pi v/2a,\ W = 1.5v/a$

(b) $\tilde{t} = 1.1\pi v/2a,\ W = 3v/a$

(c) $\tilde{t} = 1.1\pi v/2a,\ W = 5v/a$

(d) $\tilde{t} = 1.5v/a,\ W = 1.5v/a$

Supplementary Fig. 12. LCMs of $H'_{8B}$ in given disorder configurations with $A = 1.2$, $E_F = 0$ and $80 \times 80$ cells. Each pixel in the color maps corresponds to one cell. The open boundary condition is applied. LCMs with $|C(\vec{R})| > 1.5$ will be mapped to $\pm 1.5$ in the color maps. For (a) $\sim$ (c), $\tilde{t}$ is fixed on $1.1\pi v/2a$ and $aW/v = 1.5,\ 3,\ 5$, respectively. (d) LCM with $\tilde{t} = 1.5v/a$ and $W = 1.5v/a$.

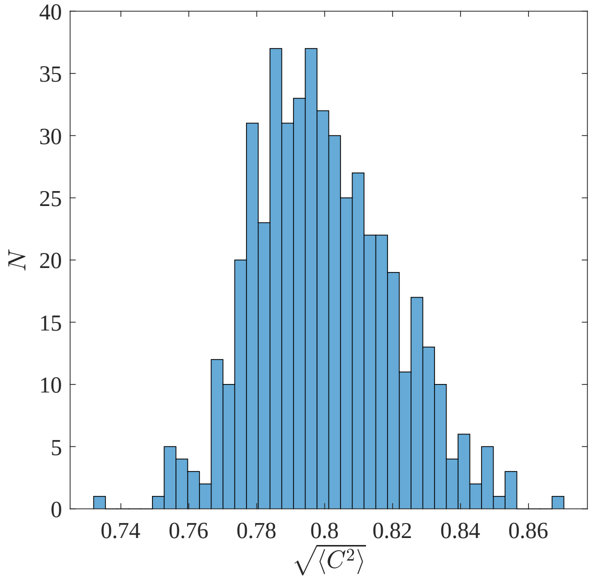


(a) $\tilde{t} = \pi v/2a,\ W = 2v/a,\ N_{sample} = 500$

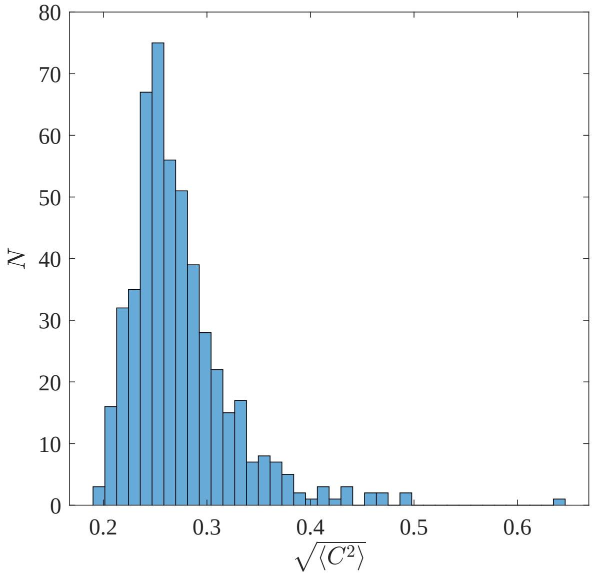


(b) $\tilde{t} = \pi v/2a,\ W = 7v/a,\ N_{sample} = 500$

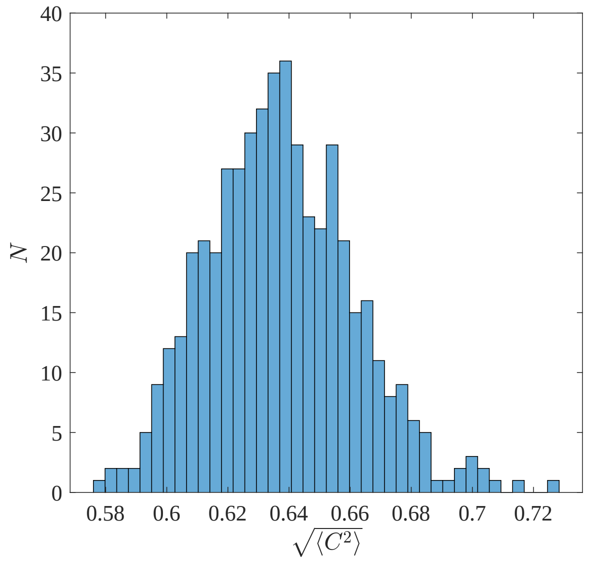


(c) $A = 1.2,\ \tilde{t} = 1.1\pi v/2a,\ W = 1.5v/a,\ N_{sample} = 500$

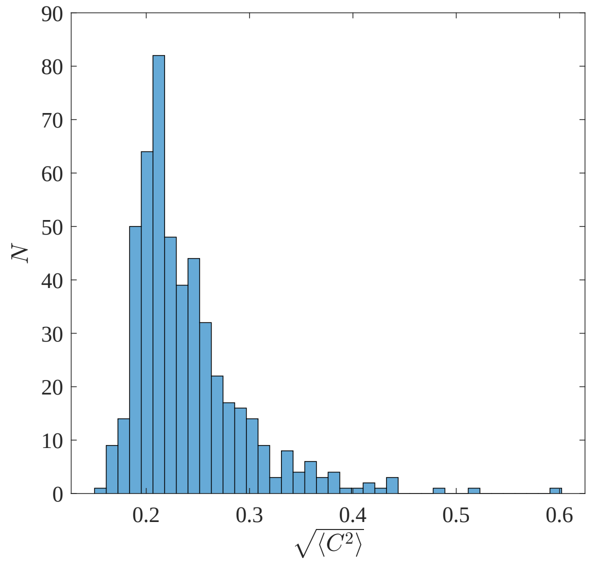


(d) $A = 1.2,\ \tilde{t} = 1.1\pi v/2a,\ W = 5v/a,\ N_{sample} = 500$

Supplementary Fig. 13. Distributions of the root mean squares of LCM in lattice models with $E_F = 0$, open boundary condition and different strengths of disorder. Each plot contains 500 disorder configurations and each sample comprises $80 \times 80$ cells. (a) Distribution of $H_{8B}$ in CMP ($\tilde{t} = \pi v/2a,\ W = 2v/a$). (b) Distribution of $H_{8B}$ in LP ($\tilde{t} = \pi v/2a,\ W = 7v/a$). (c) Distribution of $H'_{8B}$ in CMP ($A = 1.2,\ \tilde{t} = 1.1\pi v/2a,\ W = 1.5v/a$). (d) Distribution of $H'_{8B}$ in LP ($A = 1.2,\ \tilde{t} = 1.1\pi v/2a,\ W = 5v/a$).

## II. Critical metal phase in generic magnetic point groups

The models we have extensively investigated are characterized by $C_{2z}T$ on average and transition of the Stiefel-Whitney class. Since we are motivated by the semi-classical picture of conducting percolation, which is not limited to Manhattan lattice and can be protected by average symmetries other than $C_{2z}T$, CMP should not be limited to Manhattan network, $C_{2z}T$ and the Stiefel-Whitney class (a special case of Real Space Invariance in $C_{2z}T$). In this section, we will first show two additional models that have CMPs. The first model is a variant of $H'_{8B}$ (see Supplementary Fig. 27) with average symmetries $C_{4z}T$ and $m_{xy}$. The second model is a Kagome-like network model whose percolation picture is validated by average $C_{2z}T$. Then, we will discuss several simple magnetic groups that can protect the percolation picture and how they can support CMP during OAI transitions.

### A. Two additional models of CMP

First, notice that more than one average symmetry in $H'_{8B}$ can protect the percolation mechanism. $C_{4z}T$, $m_{xy}$, and glides along $x$ and $y$ axes all can protect the equal ratio of $C = \pm 1$. Hence, the percolation mechanism is still protected even if we break some of the symmetries. In addition, since a gapless band structure in the clean limit is necessary for a delocalized phase, we have to keep two OAI limits at $a\tilde{t}/v = 0, \infty$ inequivalent so that the transition will enforce a gap closure. For example, $C_{4z}T$ and $m_{xy}$ can protect the percolation mechanism alone since both can reverse the Chern number. However, the two OAI limits are adiabatically connected if only one of them is present. Hence, to enforce a gapless region supporting CMP, we need both $C_{4z}T$ and $m_{xy}$. We design the model $H''_{8B}$ (Supplementary Fig. 14(a)) by multiplying half of the edge hoppings $t'_+$ in $H'_{8B}$ by a complex factor $q = t''_+/t'_+$. The symmetry group of $H''_{8B}$ is $P4'm'm$ (# 99.165 in BNS setting) containing both $C_{4z}T$ and $m_{xy}$. Similar to $H'_{8B}$, the OAI transition ($a\tilde{t}/v = 0 \to \infty$) of $H''_{8B}$ also induces a braiding of four Dirac points stabilized at the zero energy. The numerical data (see Supplementary Fig. 14(b)) confirms that CMP survives in $H''_{8B}$.

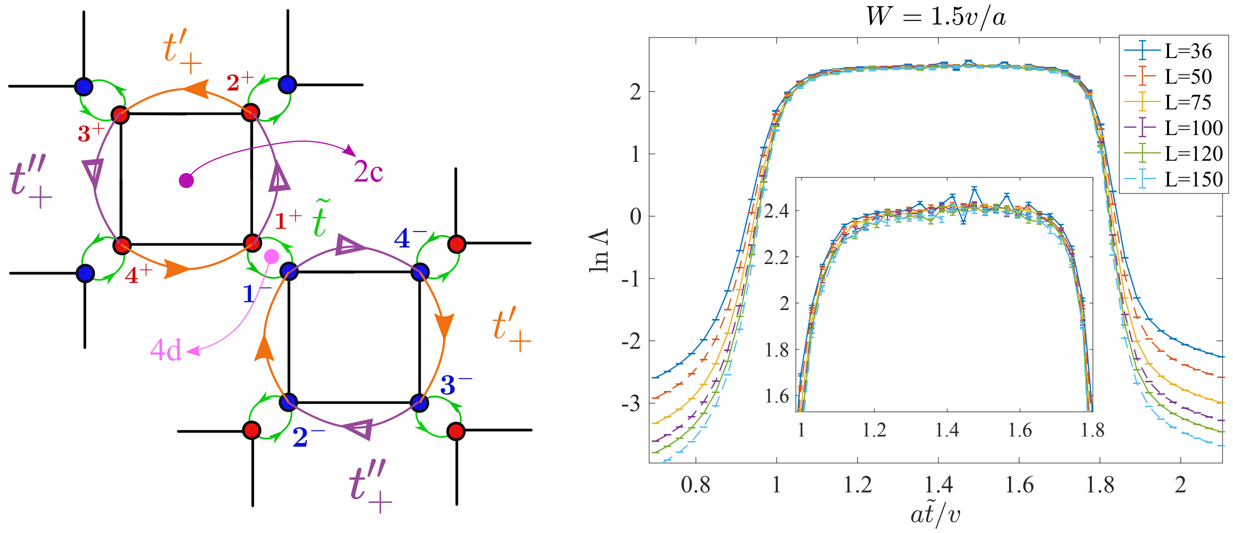


(a) (b)

Supplementary Fig. 14. Unit cell and localization length of the modified model $H''_{8B}$. (a) Unit cell of $H''_{8B}$. Each unit cell consists of eight atoms indicated by $(1 \sim 4)^{\pm}$. The orange, purple and green arrows correspond to the modified square edge hoppings $t'_+ = -(1 + Ai)\pi v/4a\,(A \in \mathbb{R}^+)$, $t''_+ = qt'_+\,(q \in \mathbb{C})$, and the inter-square hoppings $\tilde{t}\,(a\tilde{t}/v \in \mathbb{R})$, respectively. The 2c and 4d indicate the representative positions of corresponding Wyckoff positions. (b) Localization length $\Lambda$ as the function of $\tilde{t}$ of $H''_{8B}$ with $A = 1.2$, $q = 0.625\exp(-0.15\pi)$, $W = 1.5v/a$ and $E_F = 0$. The errorbars indicate the standard deviation (SD) of data points. Different colored solid and dashed lines represent data from different transversal sizes specified by the legend inside the panel. The longitudinal size is $M = 10^7$ and the data precision $(\sigma_\Lambda/\Lambda)$ reaches $1.5\%$. The inset shows the zoomed critical region.

Second, we can consider a Kagome-like network (Supplementary Fig. 15(a)) whose scattering nodes locate at the intersections of chiral wires. Each scattering node is identical to that of the Manhattan network (Supplementary Fig. 16(b), Eq. (19)). The Kagome-like network respects $C_{2z}T$ and can also mimic a tricolor percolation process. As demonstrated by Supplementary Fig. 15(b), it has a CMP similar to the Manhattan network.

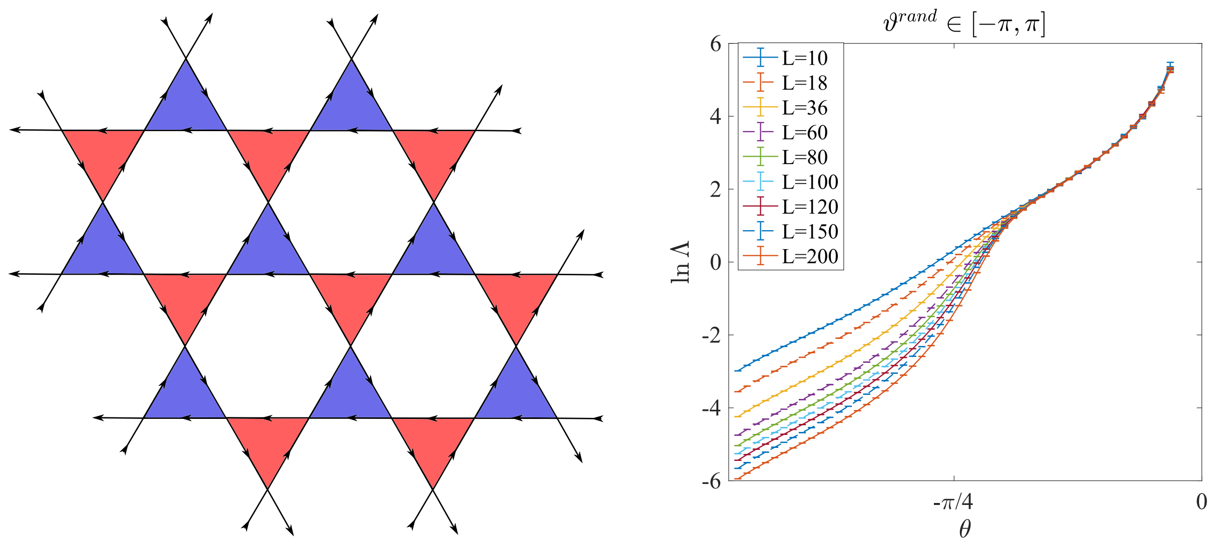


(a) (b)

Supplementary Fig. 15. Profile and localization length of the Kagome-like network model. (a) Profile of the Kagome-like network model. The arrows indicate the chirality of chiral wires. Red and blue colors indicate the chiralities of the triangles. The intersections of wires correspond to scattering nodes with effects defined in Eq. (19). (b) Localization length $\ln \Lambda$ versus scattering angle $\theta$ with different transversal sizes $L$ in the Kagome-like network model. Different colored solid and dashed lines represent data from different transversal sizes specified by the legend inside the panel. The longitudinal size is $M = 10^7$. The data precision $\sigma_\Lambda/\Lambda$ reaches 1% for $\theta < -\pi/8$, 1.5% for $-\pi/8 < \theta < -\pi/16$ and 3% for other data points.

### B. CMP in generic magnetic point groups

According to the above results, CMP could be a general phenomenon when an average symmetry protects a conducting percolation phase during a transition between inequivalent OAIs. However, we do not claim these conditions are sufficient for CMP. As we have discussed in Sec. D of the main text, the number of Dirac points and correlations of disorder-induced Dirac masses are also important. To further show the generality of CMP, we inspect the minimal OAI transitions of all the simple symmetries that can protect the percolation picture alone and how a CMP could arise. More precisely, we restrict ourselves to four simple (magnetic) space groups ($Pm$, $P2'$, $P4'$, $P6'$) with and without Spin-Orbit Coupling (SOC). The results are summarized and explained in Fig. 6 of the main text. In this section we introduce the method to obtain the results.

The quantity we used to characterize inequivalent OAIs is the real Space Invariant (RSI) [6]. RSI for a given (magnetic) space group is a collection of expressions of orbital occupations at inequivalent Wyckoff positions. RSI is invariant under symmetric charge additions/subtractions on Wyckoff positions, *i.e.*, RSI is invariant under adiabatic transitions that change orbital occupations without gap closing. Hence, for a given symmetry group, transitions between two OAIs with different RSIs must go through a gap closing, *i.e.*, they are inequivalent OAIs that cannot be connected adiabatically. With the help of RSI, we can classify OAIs for a given symmetry group. Further, by expressing RSI in terms of momentum irreps, we can deduce the band deformation and how a CMP can arise during the transition.

Let us take $P4'$ with and without SOC as pedagogical examples. The Wyckoff positions of $P4'$, except for the general positions, are 1a $(0, 0)$, 1b $(1/2, 1/2)$ and 2c $(1/2, 0)\&(0, 1/2)$ with site symmetries $4'$, $4'$ and 2, respectively. Taken from MBANDREP program on the Bilbao Crystallographic Server [7], the irreducible corepresentations of site symmetry $4'$ are A (even under $C_{2z}$) and BB (odd under $C_{2z}$) without SOC. And the co-irreps of site symmetry 2 are $A$ (even under $C_{2z}$) and $B$ (odd under $C_{2z}$).

The Wannier centers of occupied states of a Hamiltonian can adiabatically move if their displacements preserve symmetry. Hence, RSI should be invariant under the symmetric addition/subtraction of electrons. For a given Wyckoff position, these electrons will form an induced representation of the site symmetry. For 1a or 1b here, the induced representation is $A \oplus A \oplus BB$ since four electrons related by $C_{4z}T$ form two $C_{2z}$-even and two $C_{2z}$-odd orbitals. For the same reason, the induced representation on each 2c position is $A \oplus B$. Denote $m_q(R)$ as the occupation number of co-irrep $R$ at each Wyckoff position $q$, we can define RSI at 1a/1b as $\delta_{a/b} = m_{a/b}(A) - 2m_{a/b}(BB)$ and RSI at 2c as $\delta_c = m_c(A) - m_c(B)$. One can check that the RSI is invariant under symmetric electron addition/subtraction on 1a, 1b and 2c.

To see the band deformation during OAI transitions, we should express the above RSI in terms of momentum irreps at high-symmetry points. One can find a full derivation in the Appendix C.3 of [8]. The basic idea is that an occupied molecular orbital

$R$ on the Wyckoff position $q$ will introduce an MEBR $R_q \uparrow G$ (a group of bands with specific irreps at high-symmetry points) in the momentum space, hence RSI originally expressed by orbital occupations can be expressed by occupations of momentum irreps.

We skip the full derivation and directly put the results of $P4'$ without SOC here

$$\begin{aligned} \delta_a &= m(X_1) - m(\Gamma_2\Gamma_2) - m(M_2M_2) \\ \delta_b &= n_{\text{band}} - m(\Gamma_2\Gamma_2) - m(X_1) - m(M_2M_2) \\ \delta_c &= m(M_2M_2) - m(\Gamma_2\Gamma_2) \end{aligned} \tag{10}$$

where $m(R)$ indicates the occupation number of momentum irrep $R$, and $n_{\text{band}}$ is the total number of occupied bands. One can find the definitions irreps in Eq. (10) with the help of MSITESYM program on the Bilbao Crystallographic Server [7].

In Sec. II.D of the main text, we have defined the concept of minimal OAI transitions which have the simplest band deformations and can be viewed as the building block of generic OAI transitions. Eq. (10) shows that OAI transitions in $P4'$ without SOC must change the occupation numbers of $\Gamma_2\Gamma_2$, $M_2M_2$, and $X_1$. Also, we always restrict ourselves to the transitions preserving the number of occupied bands. Hence, we have three minimal OAI transitions that changes one of these occupation individually. These three minimal transitions correspond to three rows of the "$P4'$-NSOC" block of Fig. 6 in the main text. The first transition changes $m(\Gamma_1)$ only. Since there are only two irreps at the $\Gamma$ point – one dimensional $\Gamma_1$ and two dimensional $\Gamma_2\Gamma_2$, the first minimal transition will decrease $m(\Gamma_1)$ by 2 and increase $m(\Gamma_2\Gamma_2)$ by 1. Certainly, the inverse process is also a minimal OAI transition, but we will not discuss it since the property is the same. During this transition, two 1-dim irreps $\Gamma_1$ is replaced by one 2-dim irrep $\Gamma_2\Gamma_2$. Hence, if we control the transition by some parameter, there should be a finite parameter region where only one $\Gamma_1$ goes up across the Fermi surface and the low-energy physics is dominated by a quadratic touching from $\Gamma_2\Gamma_2$.

The $k \cdot p$ model of the quadratic touching is

$$H(\vec{k}) = (k_x^2 - k_y^2)\sigma_x + c k_x k_y \sigma_y \quad c = \text{Const}, \tag{11}$$

where $\vec{k}$ is the momentum deviation from $M$ point. We have taken $C_{4z}T = i\sigma_y\mathcal{K}$ since $\Gamma_2\Gamma_2$ comes from the corepresentation $BB$ where $C_{2z} = (C_{4z}T)^2 = -1$.

Now, suppose we introduce slow-varying disorders that respect $P4'$ on average, Eq. (11) can locally open a mass gap, *i.e.*,

$$H(\vec{k}, \vec{r}) = (k_x^2 - k_y^2)\sigma_x + c k_x k_y \sigma_y + m(\vec{r})\sigma_z, \tag{12}$$

which results in a Chern number $\propto \text{sign}(cm(\vec{r}))$. Since $C_{4z}T$ reverses the sign of Chern number and protects the percolation picture, we can expect a CMP in this OAI transition.

As to the minimal change of orbital occupation, Eq. (10) tells us that the first minimal OAI transition will change RSI by $\Delta\delta_a = \Delta\delta_b = \Delta\delta_c = -1$. According to RSI in terms of orbital occupations, one can check that $\Delta m_a(A) = \Delta m_b(A) = -1$, $\Delta m_c(B) = 1$ is the minimal orbital transition realizing the RSI change and preserving the particle number. Both the band deformation and real space orbital transition are illustrated in the first row of block "$P4'$-NSOC" of Fig. 6 in the main text.

The second minimal OAI transition will replace two 1-dim irreps $M_1$ by one 2-dim irrep $M_2M_2$, and hence is similar to the first minimal transition. Both the band deformation and real space orbital transition are illustrated in the second row of block "$P4'$-NSOC" of Fig. 6 in the main text.

The third transition replaces a 1-dim irrep $X_2$ by a 1-dim irrep $X_1$. Due to symmetry $C_{4z}T$, there will also be an exchange at the $Y$ point. Hence, the band gap will closed at a single parameter point where two Dirac cones appear at $X$ and $Y$ points. According to the argument in Sec. II.D of the main text, it can be delocalized if the two disorder-induced Dirac masses are positively correlated. Both the band deformation and real space orbital transition are illustrated in the third row of block "$P4'$-NSOC" of Fig. 6 in the main text.

Now we consider $P4'$ with SOC. In this case, the only site symmetry irrep of $4'$ is 2-dim ${}^1E^2E$. Hence the induced representation of symmetric electron addition is ${}^1E^2E \oplus {}^1E^2E$ and we should define $\delta_{a/b} = \mod(m_{a/b}({}^1E^2E), 2)$. The site symmetry irreps of 2 are 1-dim ${}^1E$ and ${}^2E$ where the $C_{2z}$ action are $i$ and $-i$, respectively. Hence, the induced representation of symmetric electron addition on each c position is ${}^1E \oplus {}^2E$ and the RSI of 2c should be $\delta_c = m_c({}^1E) - m_c({}^2E)$

However, there is a significant difference to the non-SOC case, not all the components of an RSI can be expressed in terms of momentum irreps. The reason is that the MEBRs of different orbitals can behave the same in terms of momentum irreps. More precisely, we can view MEBRs as vectors whose components are occupation numbers of momentum irreps. Then we can consider a matrix $R_{MEBR}$ comprises column vectors of all the involved MEBRs, *e.g.*, ${}^1E^2E_a \uparrow G$, ${}^1E^2E_b \uparrow G$, ${}^1E_c \uparrow G$, and ${}^2E_c \uparrow G$ for $P4'$ with SOC. In general, the involved MEBRs are linear dependent, *i.e.*, $\text{rank}(R_{MEBR}) < n_{\text{MEBR}}$ where $n_{\text{MEBR}}$ is the number of MEBRs. Denote the number of components of RSI as $n_{RSI}$, only $\min\{\text{rank}(R_{MEBR}), n_{RSI}\}$ (linear combinations of) RSIs can be expressed by momentum irreps (see Appendix C.3 [8] for details).

$$\begin{aligned} \delta_{a+b} &= \text{mod}\left(m_a({}^1E^2E) + m_b({}^1E^2E), 2\right) = \text{mod}(m(X_4), 2) \\ \delta_c &= 2n_{\text{band}} - m(X_4) \end{aligned} \tag{13}$$

Again, one can find the definitions of irreps $X_4$ by MSITESYM program on the Bilbao Crystallographic Server [7]. Now we have two kinds of minimal OAI transitions preserving the number of occupied bands. See the "$P4'$-SOC" block of Fig. 6 for an illustration, the first one increases $m(X_4)$ by 1 while the second one is invisible to quantities in Eq. (10).

The first minimal OAI transition is similar to the third minimal transition of $P4'$ without SOC. Since there are only two 1-dim irreps $X_3$, $X_4$ at the $X$ points, the first minimal OAI transition will induce a band inversion at the $X$ point replacing irrep $X_3$ by $X_4$. Due to $C_{4z}T$, there is also a band inversion at the $Y$ point. Hence, the first transition will go through a parameter point where two Dirac cones appear at the $X$ and $Y$ points. According to the discussion in Sec. II.D, it can be a critical point when the disorder-induced two Dirac masses are positively correlated. Both the band deformation and real space orbital transition are illustrated in the first row of block "$P4'$-SOC" of Fig. 6 in the main text.

The second transition is different from previous transitions. It increases $\delta_a$ by 1 and decreases $\delta_b$ by 1, and hence is invisible to the quantities in Eq. (13). This transition will not induce band inversions at high-symmetry points, rather, the gap will close at general k-points. Due to symmetry $C_{4z}T$, there will be at least four touching points. Also note that, if the transition is controlled by some parameter, the touching points in general can only exist at a parameter point. The low-energy physics of these touching points can be described by a Hamiltonian of four Dirac cones related by $C_{4z}T$. Such a Hamiltonian can open a gap perturbatively without breaking $C_{4z}T$. Hence, rather than going through a braiding of Dirac points, the band structure will go through a gap closure followed by an immediate reopening during the transition. Again, according to the discussion in Sec. II.D in the main text, the parameter point with four Dirac cones can be delocalized. Both the band deformation and real space orbital transition are illustrated in the second row of block "$P4'$-SOC" of Fig. 6 in the main text.

Apply the above procedure to other groups under consideration ($Pm$, $P2'$, $P6'$), we can identify the features of the minimal OAI transitions and how could they support CMPs. Since both the reciprocal and real space information of these transitions are summarized in Fig. 6 in the main text, we will only list RSIs of these groups here.

For $Pm$, we take the reflection as $m_y$ ($(x,y) \to (x,-y)$) and the Wyckoff positions are 1a $(x,0)$ and 1b $(x,1/2)$. The site symmetry for both 1a and 1b is $m_y$ whose irreps are $A'$ (even under reflection) and $A''$ (odd under reflection) without SOC. The symmetric electron addition/subtraction will induce representation $A' \oplus A''$ on each Wyckoff position. Hence, we have RSI:

$$\begin{aligned}\delta_a &= m_a(A') - m_a(A'') = m(X_1) - m(M_2)\\ \delta_b &= m_b(A') - m_b(A'') = m(X_1) + m(M_2) - n_{\rm band}.\end{aligned} \tag{14}$$

Here we use a different notation to that of Bilbao Crystallographic Server which denotes $X_a$, $M_a$ as $Y_a$, $C_a$, respectively. Although the minimal OAI transitions of $Pm$ are realized by band inversions, the transition will go through a gapless parameter region rather than a point. The first minimal transition (first row of "$Pm$-NSOC" block of Fig. 6 in main text) replaces one $X_2$ by $X_1$. According to MSITESYM program on the Bilbao Crystallographic Server [7], $X_1$ only comes from MEBR $A'_{a,b}\uparrow G$ while $X_2$ only comes from $A''_{a,b}\uparrow G$. Hence, mirror reflection action is 1 $(-1)$ on $X_1$ ($X_2$), and the first minimal transition will change the parity of $X$ point under reflection. Also notice that all points along $k_x$ axis have well-defined and identical reflection parities. Hence, to change the parity of the $X$ point, the band inversion should create at least a pair of Dirac points that moving along the $k_x$ axis and change its parity. Hence, this transition can induce a finite parameter region where two Dirac appears at the $k_x$ axis and a CMP is supported when the disorder-induced Dirac mass terms are positively correlated.

The situation is similar for transitions that changes $m(M_2)$ only. Although $M_2$ comes from both reflection parities ($A''_a\uparrow G$, $A'_b\uparrow G$), RSI in terms of orbitals Eq. (14) suggests that the transition is realized by $A'_{a/b} \leftrightarrow A''_{b/a}$. Hence the transition will create at least a pair of Dirac points moving along the $M-Y-M$ line and change its parity. Also, it turn out that $Pm$ with SOC gives out the same results as no SOC.

For $P2'$, the Wyckoff positions are 1a $(0,0)$, 1b $(1/2,0)$, 1c $(0,1/2)$ and 1d $(1/2,1/2)$. They have the same site symmetry $2'$ and irrep $A$ without SOC. Note that SOC is irrelevant in $P2'$ since we have $(C_{2z}T)^2 = {}^d1\,T^2 = 1$ for spinful electrons, and hence SOC will not affect the representation of $P2'$. Now the induced representation of symmetric electron addition/subtraction is $A \oplus A$ and the RSI should be

$$\delta_{a/b/c/d} = \mathrm{mod}(m(A_{a/b/c/d}), 2) \tag{15}$$

Only their summation $\delta_{a+b+c+d} = \mathrm{mod}_2(m(A_a) + m(A_b) + m(A_c) + m(A_d), 2)$ can be expressed in the momentum space

$$\delta_{a+b+c+d} = \mathrm{mod}(n_{\rm band}, 2) \tag{16}$$

Hence, the minimal OAI transition respecting the number of occupied bands will close the band gap at a general k-point. The Dirac Hamiltonian respecting $C_{2z}T$ cannot open a gap perturbatively. Hence, the transition will go through a finite parameter region with at least two Dirac points in contrast to the case of $C_{4z}T$.

For $P6'$, the Wyckoff positions are 1a $(0,0)$, 2b $(1/3,2/3)$ & $(2/3,1/3)$ and 3c $(1/2,0)$ & $(0,1/2)$ & $(1/2,1/2)$. The site symmetry of 1a is $6'$ and the irreps are $A_1$ and ${}^1E{}^2E$ without SOC. $A_1$ is one-dimensional and the $C_{3z} = (C_{6z}T)^2$ action is trivial. ${}^1E{}^2E$ is two-dimensional and the $C_{3z}$ action is $\mathrm{diag}(e^{2\pi i/3}, e^{-2\pi i/3})$. Hence, the symmetric electron addition/subtraction will change both $m_a(A_1)$ and $m_a({}^1E{}^2E)$ by 2. As to 2b, the site symmetry is 3 and irreps are $A_1$ ($C_{3z}=1$), ${}^1E$ ($C_{3z} = e^{-2\pi i/3}$),

and $^2E$ ($C_{3z} = e^{2\pi i/3}$). All of these irreps are one-dimensional. Hence, the symmetric electron addition/subtraction will change all of $m_b(A_1)$, $m_b(^1E)$, $m_b(^2E)$ by 1 As to 3c, the site symmetry is $2'$ and the only 1-dim irrep is A without SOC. Hence the symmetric electron addition/subtraction will change $m_c(A)$ by 2. From the above, we can conclude the RSI:

$$\begin{aligned} \delta_{a1} &= m_a(A_1) - m_a(^1E^2E) \\ \delta_{a2} &= \text{mod}(m_a(A_1), 2) \\ \delta_{b1} &= m_b(A_1) - m_b(^1E) \\ \delta_{b2} &= m_b(A_1) - m_b(^2E) \\ \delta_c &= \text{mod}(m_c(A), 2) \end{aligned} \tag{17}$$

Only four (conbinations) of them can be translated to band indicators:

$$\begin{aligned} \delta_{a1} &= n_{\text{band}} - m(\Gamma_2\Gamma_3) - m(K_2K_3) - m(K_2'K_3') \\ \delta_{b1} &= m(K_2'K_3') - m(\Gamma_2\Gamma_3) \\ \delta_{b2} &= m(K_2K_3) - m(\Gamma_2\Gamma_3) \\ \delta_{a2+c} &= \text{mod}(m_a(A_1) + m_c(A), 2) = \text{mod}(n_{\text{band}}, 2) \end{aligned} \tag{18}$$

There are four minimal OAI transitions as illustrated by the "$P6'$-NSOC" block of Fig. 6 in the main text. The first three correspond to band inversions at $\Gamma$, $K$ and $K'$ points respectively. As the first minimal transition of $P4'$-NSOC, all of them will go through finite parameter regions with quadratic touchings from 2-dim irreps near the Fermi level. The last minimal transition decreases (increases) $m_a(A_1)$, $m_a(^1E^2E)$ by 1 and increase (decreases) $m_c(A)$ by 1. Hence, the last transition is invisible to quantities in Eq. (18) and will close band gap at (at least) 6 general k-points enforced by $C_{6z}T$. Since $(C_{6z}T)^3 = C_{2z}T$ should be respected, the effective Dirac Hamiltonian cannot open a gap perturbatively. According to the discussion in Sec. II.D of the main text, a CMP can exist in the parameter region where the touching points braid each other.

The situation of $P6'$ with SOC is the same to $P6'$ without SOC. When spin is under consideration, $(C_{6z}T)^6 = 1$, $(C_{6z}T)^2 = -C_{3z}$, and hence the representations of double group is the same to that of the original group.

We would like to end this section here. One can directly generalize the above argument to more complicated magnetic groups. In a summary, by analyzing band deformations of OAI transitions in several magnetic space groups, we have seen that CMPs are commonly supported by average symmetries protecting the percolation mechanism. Therefore, CMPs in 2D class A systems have a general relevance and whose nature deserves further investigation.

## III. Network model

### A. Network model on the Manhattan lattice

The wires in Supplementary Fig. 16(a) represent the 1D chiral modes with directions indicated by the arrows. There is a $\delta$ scattering potential at every intersection of the wires. We can view the red (blue) squares as Chern blocks with Chern number $C = 1(-1)$ since the edges of colored squares can be viewed as the edge state of local Chern insulators. The scattering potentials give rise to tunnelings between Chern blocks.

The network model has $C_{2z}T = \{2'_{001}|0,0,0\}$, $C_{4z} = \{4^+_{001}|0,\frac{1}{2},0\}$ and $M_{xy} = \{m_{1\bar{1}0}|0,0,0\}$ symmetries, where the fractional translations are in units of the lattice constant $2a$ (Supplementary Fig. 16(a)), *i.e.*, $\{R|\alpha,\beta,\gamma\}$ represents a rotation or/and reflection operation $R$ followed by a translation operation $\vec{t} = (2\alpha a, 2\beta a, 2\gamma a)$. As to the rotation/reflection part, $2'_{001}$ represents the combination of a $\pi$-rotation along the $z$-direction and a time-reversal operation; $4^+_{001}$ represents an anticlockwise $\pi/2$-rotation viewed from the positive z-direction; $m_{1\bar{1}0}$ represents a reflection whose normal is along the $(1,-1,0)$-direction. The $C_{2z}T$ and $C_{4z}$ centers locate at the intersections and Chern block centers, respectively. The mirror plane of $M_{xy}$ is indicated by the dashed line in Supplementary Fig. 16(a). $C_{4z}$ rotations transform the red (blue) squares into red (blue) squares while $C_{2z}T$ transform red (blue) into blue (red) ones. Notice that the $C_{2z}T$ centers and $C_{4z}$ centers are not coincident, hence the joint action $C^2_{4z}C_{2z}T \neq T$. Actually, these symmetries generate a type-IV magnetic space group $P_C4bm$ (#100.177 in BNS setting) with the magnetic translation given by $\widetilde{T} = \{1'|\frac{1}{2},\frac{1}{2},0\} = C_{2z}T \cdot C^2_{4z} \cdot \{1|0,1,0\}$, where $1'$ represents the time reversal operation. (Here, we adopt a different convention of the origin point as the MGENPOS program on the Bilbao Crystallographic Server [9]. To obtain our symmetry operations, one should specify the "origin shift" as $(\frac{1}{4},-\frac{1}{4},0)$ when using the MGENPOS program.)

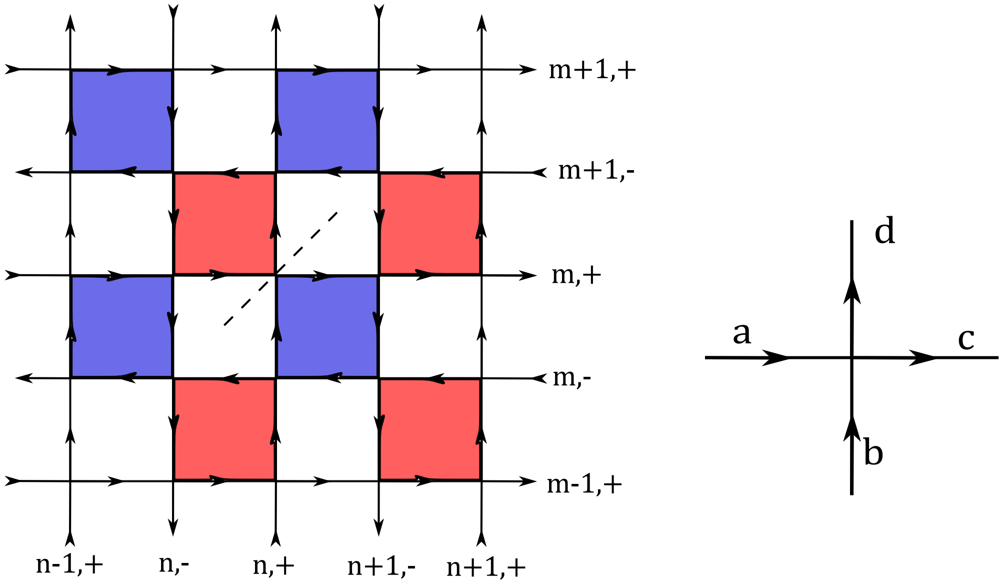


(a) (b)

Supplementary Fig. 16. Network and the scattering node. (a) Profile of the network model. A vertical (horizontal) wire labeled by $n,\alpha$ ($m,\alpha$) lies in $x = 2a(n+\frac{1+\alpha}{4})$ ($y = 2a(m+\frac{1+\alpha}{4})$). Here, $\alpha = \pm 1$, $a$ is the side length of the colored squares, and the lattice constant is $2a$. The solid lines represent chiral wires with chiralities indicated by arrows. The intersections correspond to scattering nodes defined in Eq. (19). Red and blue colors indicate the chiralities of the squares. The dashed line indicates the symmetric mirror plane. (b) Channels of a scattering node. The input channel a/b will go straight into the output channel c/d if there is no scattering potential. Arrows here have the same meanings as the left panel.

We label the four channels involved in a scattering node as a, b, c, and d. (see Supplementary Fig. 16(b)). One can regard $a$ & $b$ as the incident channels and $c$ & $d$ as the outcoming channels. The scattering potential is defined by its action on the channels:

$$\begin{pmatrix} \psi_c(0) \\ \psi_d(0) \end{pmatrix} = \begin{pmatrix} \cos\theta & -i\sin\theta \\ -i\sin\theta & \cos\theta \end{pmatrix} \begin{pmatrix} \psi_a(0) \\ \psi_b(0) \end{pmatrix} \qquad \theta \in [-\pi/2, \pi/2], \tag{19}$$

where $\psi_j(0)$ is the amplitude of channel $i$ near the scattering node. Notice that the scattering effect of $\theta + \pi$ is merely that of $\theta$ multiplied by $-1$ which can be removed by redefining $\psi_c$ and $\psi_d$. It is compatible to take such a redefinition on every

intersection, and hence the effect of replacement $\theta \to \theta + \pi$ is a pure gauge transformation. Therefore, we take $\theta \in [-\pi/2, \pi/2]$ rather than $\theta \in [-\pi, \pi]$. Also, as we will see in Supplementary Sec. I A, $\theta \in [-\pi/2, \pi/2]$ is enough to realize a localized-delocalized-localized percolation transition.

When $\theta = -\pi/2$, the network decouples to disconnected red ($C = 1$) and blue ($C = -1$) squares. The electrons then form local loop currents surrounding them. According to Eq. (19), an electron in a local current will obtain a $\pi/2$ phase at each corner when going along the arrows and accumulate a $2\pi$ phase after going around. In other words, an electron will obtain a phase $2\pi\,(-2\pi)$ after going around the red (blue) square anticlockwise, showing that the red (blue) squares indeed has Chern number $1\,(-1)$. Hence one can view the local orbitals indicated by red squares as having "angular momentum" $L = 1$, and the blue ones have $L = -1$. When $\theta = \pi/2$, we also have a group of decoupled squares, but the phase jump will be reversed, leading to "angular momentum" $L = -1$ for red squares and 1 for blue ones. So in both limits ($\theta = \pm\pi/2$), the system comprises a group of well-separated local currents and hence is localized, but the two limits have opposite "angular momentum" at each square.

The disorder in this paper is the random size of Chern blocks which can be realized by the random phases on square edges in the network. More discussion about the disorder can be found in Supplementary Sec. IV F. In this section, we focus on the clean limit, in which the system has to go through a metallic phase when we change $\theta$ from $-\pi/2$ to $\pi/2$. When $\theta = 0$, the network is merely two bundles of decoupled chiral wires states and is hence gapless.

### B. Effective Hamiltonian $H_N$ and band structure of the network model

We use the wires in the limit $\theta = 0$ as the basis of the network model and denote the fermion annihilation operator on horizontal and vertical wires as $\psi_h(x; m, \alpha)$ and $\psi_v(y; n, \alpha)$, respectively. Note that we use different notations from Eq. (2) except for the direction subscript $d = h, v$. Here, $x$ ($y$) is the coordinate inside a horizontal (vertical) wire, and $m$ ($n$) & $\alpha = \pm 1$ together label different horizontal (vertical) wires (see Supplementary Fig. 16(a), where $(m, n)$ indicates the unit cell and $\alpha$ indicates the chirality). The indices $\xi$ and $l$ in Eq. (2) correspond to $x$ or $y$ and $(2m + \alpha)a$ or $(2n + \alpha)a$, respectively. We choose a global coordinate system in which $\psi_h(x; m, \alpha)$, $\psi_v(y; n, \alpha)$ act on $(x, y = (2m + (1 + \alpha)/2)a)$, $(x = (2n + (1 + \alpha)/2)a, y)$, respectively. Here $a$ is the side length of the Chern squares. The unit cell $(m, n)$ corresponds to the intersection region of two horizontal wires $(m, -)$ & $(m, +)$ and two vertical wires $(n, -)$ & $(n, +)$. These annihilation and creation operators are subject to the anti-commutation relations

$$\{\psi_h(x; m, \alpha), \psi_h^\dagger(x'; m', \alpha')\} = \delta_{m,m'}\delta_{\alpha,\alpha'}\delta(x - x'), \qquad \{\psi_v(y; m, \alpha), \psi_v^\dagger(y'; m', \alpha')\} = \delta_{m,m'}\delta_{\alpha,\alpha'}\delta(y - y')\ . \tag{20}$$

The Hamiltonian of the network model $H_N$ has two parts. The first part is the decoupled wires:

$$H_{N,0} = \sum_{m,\alpha} \alpha v \int \mathrm{d}x\ \psi_h^\dagger(x; m, \alpha)(-i\partial_x)\psi_h(x; m, \alpha) + \sum_{n,\alpha} \alpha v \int \mathrm{d}y\ \psi_v^\dagger(y; n, \alpha)(-i\partial_y)\psi_v(y; n, \alpha)\ . \tag{21}$$

The scattering effect in Eq. (19) can be realized by the second part: *real* $\delta$-scattering potentials:

$$H_{N,1} = \sum_{m,n,\alpha,\beta} \lambda\, \psi_h^\dagger(2na + \frac{1+\beta}{2}a; m, \alpha)\, \psi_v(2ma + \frac{1+\alpha}{2}a; n, \beta)\ +\ h.c. \tag{22}$$

where $\lambda$ has the dimension of velocity. For a scattering node shown in Supplementary Fig. 16(b), we can rotate the vertical wire to $x$-direction and view the scattering as a 1D problem shown in Supplementary Fig. 17. By solving this problem, we will obtain the relation between $\lambda$ and $\theta$ of Eq. (19).

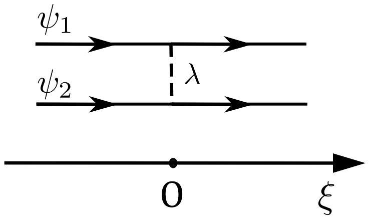


$$E \begin{pmatrix} \psi_1(\xi) \\ \psi_2(\xi) \end{pmatrix} = [-iv\partial_\xi\sigma_0 + \lambda\delta(\xi)\sigma_x] \begin{pmatrix} \psi_1(\xi) \\ \psi_2(\xi) \end{pmatrix}$$

Supplementary Fig. 17. Scattering problem of a single node

We can define $\begin{pmatrix} \psi_1(\xi) \\ \psi_2(\xi) \end{pmatrix} = \psi_+(\xi)\mathbf{e}_+ + \psi_-(\xi)\mathbf{e}_-$ on the basis basis $\mathbf{e}_+ = \begin{pmatrix} 1 \\ 1 \end{pmatrix}$, $\mathbf{e}_- = \begin{pmatrix} 1 \\ -1 \end{pmatrix}$ and decouple the Schrodinger

equation to

$$E\psi_\pm(\xi) = [-iv\frac{\mathrm{d}}{d\xi} \pm \lambda\delta(\xi)]\psi_\pm(\xi)\ . \tag{23}$$

Divide both sides by $\psi_\pm(\xi)$, we obtain

$$-iv\frac{\mathrm{d}\ln\psi_\pm(\xi)}{\mathrm{d}\xi} = E \mp \lambda\delta(\xi). \tag{24}$$

For $\xi \neq 0$, there must be

$$\psi_\pm(\xi) = \begin{cases} \psi_\pm(0^-)\exp\left(i\frac{E}{v}\xi\right) & (\xi < 0) \\ \psi_\pm(0^+)\exp\left(i\frac{E}{v}\xi\right) & (\xi > 0) \end{cases} . \tag{25}$$

Integrate Eq. (24) near zero, *i.e.*, $\int_{0^-}^{0^+}$, we have

$$\psi_\pm(0^+) = e^{\mp i\lambda/v}\psi_\pm(0^-) \Longrightarrow \begin{pmatrix}\psi_1(0^+)\\ \psi_2(0^+)\end{pmatrix} = \begin{pmatrix}\cos\frac{\lambda}{v} & -i\sin\frac{\lambda}{v}\\ -i\sin\frac{\lambda}{v} & \cos\frac{\lambda}{v}\end{pmatrix}\begin{pmatrix}\psi_1(0^-)\\ \psi_2(0^-)\end{pmatrix}. \tag{26}$$

Now we can conclude that $\theta = \lambda/v$. Recall that only $\theta \in [-\pi/2, \pi/2]$ is considered because $\theta \to \theta + \pi$ is merely a gauge transformation that will not affect the spectrum. We should take $\lambda/v \in [-\pi/2, \pi/2]$. Certainly, $\lambda/v \in [(2n-1/2)\pi, (2n+1/2)\pi]$ is also feasible, but numerical calculation with smaller $|\lambda/v|$ will be more accurate (see Eq. (33) and the discussion below Eq. (34), a larger $|\lambda/v|$ will introduce a larger cutoff error).

The actions of $C_{2z}T$, $C_{4z}$ and $M_{xy}$ are:

$$\begin{aligned}
(C_{2z}T)\psi_h(x;m,\alpha)(C_{2z}T)^{-1} &= \psi_h(-x;(-m-\frac{1+\alpha}{2}),\alpha)\\
(C_{2z}T)\psi_v(y;n,\alpha)(C_{2z}T)^{-1} &= \psi_v(-y;(-n-\frac{1+\alpha}{2}),\alpha)\\
C_{4z}\psi_h(x;m,\alpha)C_{4z}^{-1} &= \psi_v(x+a;(-m-\frac{1+\alpha}{2}),\alpha)\\
C_{4z}\psi_v(y;n,\alpha)C_{4z}^{-1} &= \psi_h(-y;(n+\frac{1+\alpha}{2}),-\alpha)\\
M_{xy}\psi_h(x;m,\alpha)M_{xy}^{-1} &= \psi_v(x;m,\alpha)\\
M_{xy}\psi_v(y;n,\alpha)M_{xy}^{-1} &= \psi_h(y;n,\alpha)
\end{aligned} \tag{27}$$

It is direct to check that

$$(C_{2z}T)(H_{N,0}+H_{N,1})(C_{2z}T)^{-1} = C_{4z}(H_{N,0}+H_{N,1})C_{4z}^{-1} = m_{xy}(H_{N,0}+H_{N,1})m_{xy}^{-1} = H_{N,0}+H_{N,1} \tag{28}$$

In addition, we should note that this model has an accidental particle-hole symmetry

$$P\psi_h(x;m,\alpha)P^{-1} = \psi_h^\dagger(x;m,\alpha) \quad P\psi_v(y;n,\alpha)P^{-1} = -\psi_v^\dagger(y;n,\alpha) \tag{29}$$

However, this will be broken after introducing the disorder (in the form of vector potential, see Supplementary Sec. IV F).

To obtain the band structure of the network model, we need to rewrite the Hamiltonian in reciprocal space. Considering the discrete translation symmetry of $H_{N,1}$, we write down the Fourier transform of $\psi_h(x)$, $\psi_v(y)$ in this form:

$$\begin{aligned}
\psi_h(x;m,\alpha) &= \frac{1}{\sqrt{NL}}\sum_{k_x,k_y}\sum_{G_x}\exp\left[i(k_x+G_x)x + ik_y\left(2m+\frac{1+\alpha}{2}\right)a\right]\phi_h(k_x,k_y;\alpha,G_x)\\
\psi_v(y;n,\alpha) &= \frac{1}{\sqrt{NL}}\sum_{k_x,k_y}\sum_{G_y}\exp\left[i(k_y+G_y)y + ik_x\left(2n+\frac{1+\alpha}{2}\right)a\right]\phi_v(k_x,k_y;\alpha,G_y),
\end{aligned} \tag{30}$$

where $L = 2Na$ is the system size in each direction, and the system contains $N \times N$ unit cells. Since $x$, $y$ are continuous variables, their Fourier co-variables should be unbounded. We use $k_j = \frac{2\pi n_j}{2Na}$ ($n_j = 0\cdots N-1$) to fit the lattice periodicity

(the "first" BZ) and the boundlessness of $k_j$ is compensated by the reciprocal vector $G_j = \frac{2\pi N_j}{2a}$ ($N_j = 0, \pm1, \pm2\cdots$). The prefactor $\frac{1}{\sqrt{NL}}$ is used to normalize the operator such that

$$\{\phi_{h/v}(k_x, k_y; \alpha, G_x), \phi^\dagger_{h/v}(k'_x, k'_y; \alpha', G'_x)\} = \delta_{k_x,k'_x}\delta_{k_y,k'_y}\delta_{\alpha,\alpha'}\delta_{G_x,G'_x} . \tag{31}$$

The inverse transformation can be written as

$$\begin{aligned}\phi_h(k_x, k_y; \alpha, G_x) =& \frac{1}{\sqrt{NL}}\sum_m \int dx \ \exp\left[-i(k_x+G_x)x - ik_y\left(2m+\frac{1+\alpha}{2}\right)a\right]\psi_h(x; m, \alpha)\\ \phi_v(k_x, k_y; \alpha, G_y) =& \frac{1}{\sqrt{NL}}\sum_n \int dy \ \exp\left[-i(k_y+G_y)y - ik_x\left(2n+\frac{1+\alpha}{2}\right)a\right]\psi_v(y; n, \alpha)\end{aligned} . \tag{32}$$

The Hamiltonian in reciprocal space can be written as

$$\begin{aligned}H_{N,0} =& \sum_{k_x,k_y}\sum_{G_x,\alpha} \alpha v(k_x+G_x)\phi^\dagger_h(k_x, k_y; \alpha, G_x)\phi_h(k_x, k_y; \alpha, G_x)\\ &+ \sum_{k_x,k_y}\sum_{G_y,\alpha} \alpha v(k_y+G_y)\phi^\dagger_v(k_x, k_y; \alpha, G_y)\phi_v(k_x, k_y; \alpha, G_y)\\ H_{N,1} =& \frac{\lambda}{2a}\sum_{k_x,k_y}\sum_{G_x,G_y}\sum_{\alpha,\beta}\exp\left[i(\frac{1+\alpha}{2})G_y a - i(\frac{1+\beta}{2})G_x a\right]\phi^\dagger_h(k_x, k_y; \alpha, G_x)\phi_v(k_x, k_y; \beta, G_y) \ + \ h.c.\end{aligned} \tag{33}$$

The symmetry operations on the reciprocal basis can also be derived as

$$\begin{aligned}C_{2z}T\phi_h(k_x, k_y; \alpha, G_x)(C_{2z}T)^{-1} =& \phi_h(k_x, k_y; \alpha, G_x)\\ C_{2z}T\phi_v(k_x, k_y; \alpha, G_y)(C_{2z}T)^{-1} =& \phi_v(k_x, k_y; \alpha, G_y)\\ C_{4z}\phi_h(k_x, k_y; \alpha, G_x)C_{4z}^{-1} =& e^{i(k_x+G_x)a}\phi_v(-k_y, k_x; \alpha, G_x)\\ C_{4z}\phi_v(k_x, k_y; \alpha, G_y)C_{4z}^{-1} =& e^{ik_x a}\phi_h(-k_y, k_x; -\alpha, -G_y)\\ M_{xy}\phi_h(k_x, k_y; \alpha, G_x)M_{xy}^{-1} =& \phi_v(k_y, k_x; \alpha, G_x)\\ M_{xy}\phi_v(k_x, k_y; \alpha, G_y)M_{xy}^{-1} =& \phi_h(k_y, k_x; \alpha, G_y)\end{aligned} . \tag{34}$$

Here the phase factors $e^{ia(k_x+G_x)}$ and $e^{iak_x}$ result from the fractional translation in the $C_{4z}$ operator. Take $\phi_h(k_x, k_y; \alpha, G_x)$ for an example:

$$\begin{aligned}C_4\phi_h(k_x, k_y; \alpha, G_x)C_4^{-1} &\overset{\text{Eq. (32)}}{=} \frac{1}{\sqrt{NL}}\sum_m \int dx \exp\left[-i(k_x+G_x)x - ik_y(2m+\frac{1+\alpha}{2})a\right] C_4\psi_h(x; m, \alpha)C_4^{-1}\\ &\overset{\text{Eq. (27)}}{=} \frac{1}{\sqrt{NL}}\sum_m \int dx \exp\left[-i(k_x+G_x)x - ik_y(2m+\frac{1+\alpha}{2})a\right]\psi_v(x+a; (-m-\frac{1+\alpha}{2}), \alpha)\\ &= \frac{e^{i(k_x+G_x)a}}{\sqrt{NL}}\sum_m \int dx' \exp\left[-i(k_x+G_x)x' + ik_y\left(-2(m+\frac{1+\alpha}{2})+\frac{1+\alpha}{2}\right)a\right]\psi_v(x'; (-m-\frac{1+\alpha}{2}), \alpha)\\ &= e^{i(k_x+G_x)a}\phi_v(-k_y, k_x; \alpha, G_x)\end{aligned} \tag{35}$$

When we numerically calculate the band structure of the network model, we have to adopt a cutoff $\Lambda_1$ for the $G_x, G_y$ indices. The hybridization introduced by $H_{N,1}$ between any two $G$ indices is on the order of $\lambda/a$. Therefore, as long as the kinetic energy at the cutoff, *i.e.*, $v\Lambda_1$, is much larger than $\lambda/a$, the off-diagonal terms between high/low lying bands and the middle bands are negligible. Hence, a high enough cutoff $\Lambda_1$ should not affect the structure of the middle bands. However, simply discarding terms in Eq. (33) that involve $|G| > \Lambda_1$ is problematic (see Supplementary Sec. III C for details). Another cutoff parameter $\Lambda_2$ on $H_{N,1}$ should be considered. The scattering between two modes with a momentum difference larger than $\Lambda_2$ will be strongly suppressed, which corresponds to a broadened $\delta$-potential with a characteristic length proportional to $1/\Lambda_2$.

In practice, we will (i) use a basis set up to the cutoff $\Lambda_1$ and (ii) omit the $\lambda$ coupling between modes with momentum

| | $\Gamma_1$ | $\Gamma_2$ | $\Gamma_3$ | $\Gamma_4$ | $\Gamma_5$ | | $M_1$ | $M_2$ | $M_3$ | $M_4$ | $M_5$ | | $X_1$ |
|---|---|---|---|---|---|---|---|---|---|---|---|---|---|
| $\{1\|0,0,0\}$ | 1 | 1 | 1 | 1 | 2 | $\{1\|0,0,0\}$ | 1 | 1 | 1 | 1 | 2 | $\{1\|0,0,0\}$ | 2 |
| $C_{2z}=\{2_{001}\|-\frac{1}{2},\frac{1}{2},0\}$ | 1 | 1 | 1 | 1 | -2 | $C_{2z}=\{2_{001}\|-\frac{1}{2},\frac{1}{2},0\}$ | $-1$ | $-1$ | $-1$ | $-1$ | 2 | $C_{2z}=\{2_{001}\|-\frac{1}{2},\frac{1}{2},0\}$ | 0 |
| $C_{4z}=\{4^+_{001}\|0,\frac{1}{2},0\}$ | 1 | -1 | -1 | 1 | 0 | $C_{4z}=\{4^+_{001}\|0,\frac{1}{2},0\}$ | $i$ | $-i$ | $-i$ | $i$ | 0 | $\{m_{100}\|0,\frac{1}{2},0\}$ | 0 |
| $M_{xy}=\{m_{1\bar{1}0}\|0,0,0\}$ | 1 | -1 | 1 | -1 | 0 | $M_{xy}=\{m_{1\bar{1}0}\|0,0,0\}$ | -1 | 1 | -1 | 1 | 0 | $\{m_{010}\|\frac{1}{2},0,0\}$ | 0 |

Supplementary Tab. I. Character table of irreps at high-symmetry momenta in magnetic space group $P_C4bm$ (#100.177 in BNS setting), taken from the COREPRESENTATIONS program on the Bilbao Crystallographic Server [7]. Characters of the listed symmetry operations can uniquely determine the irreps One should notice that we use a different convention of the origin point as the Bilbao Crystallographic Server, as explained in the second paragraph in Supplementary Sec. III A. Our $C_{2z}=\{2_{001}|-\frac{1}{2},\frac{1}{2},0\}$, $C_{4z}=\{4^+_{001}|0,\frac{1}{2},0\}$, $M_{xy}=\{m_{1\bar{1}0}|0,0,0\}$, $\{m_{100}|0,\frac{1}{2},0\}$, and $\{m_{010}|\frac{1}{2},0,0\}$ correspond to $\{2_{001}|0,0,0\}$, $\{4^+_{001}|0,0,0\}$, $\{m_{1\bar{1}0}|\frac{1}{2},-\frac{1}{2},0\}$, $\{m_{100}|\frac{1}{2},\frac{1}{2},0\}$, and $\{m_{010}|\frac{1}{2},-\frac{1}{2},0\}$ in the standard convention of the Bilbao Crystallographic Server, respectively.

| Wyckoff pos. | 2b $(\frac{3}{4}\frac{1}{4}0)$, $(\frac{1}{4}\frac{3}{4}0)$ | | | | 4c (000), $(0\frac{1}{2}0)$, $(\frac{1}{2}00)$, $(\frac{1}{2}\frac{1}{2}0)$ |
|---|---|---|---|---|---|
| Site sym. | $4m'm'$, 4 | | | | $2'm'm$, $m$ |
| MEBR | $\mathrm{A_b}\uparrow\mathrm{G}$ | $\mathrm{B_b}\uparrow\mathrm{G}$ | ${}^1\mathrm{E_b}\uparrow\mathrm{G}$ | ${}^2\mathrm{E_b}\uparrow\mathrm{G}$ | $\mathrm{A_c''}\uparrow\mathrm{G}$ |
| Orbital | 1 | $d_{x^2-y^2}+id_{xy}$ | $p_x+ip_y$ | $p_x-ip_y$ | $p_y$ |
| Irreps at $\Gamma$ | $\Gamma_1\oplus\Gamma_4$ | $\Gamma_2\oplus\Gamma_3$ | $\Gamma_5$ | $\Gamma_5$ | $\Gamma_2\oplus\Gamma_4\oplus\Gamma_5$ |
| Irreps at $M$ | $M_5$ | $M_5$ | $M_2\oplus M_3$ | $M_1\oplus M_4$ | $M_2\oplus M_4\oplus M_5$ |
| Irreps at $X$ | $X_1$ | $X_1$ | $X_1$ | $X_1$ | $2X_1$ |

Supplementary Tab. II. MEBRs of $P_C4bm$ (#100.177 in BNS setting) involved in this work, taken from the MBANDREP program on the Bilbao Crystallographic Server [7]. The real space orbital character of each MEBR is shown in the "Orbital" row. For example, the MEBR $\mathrm{B_b}\uparrow\mathrm{G}$ can be generated by an $d_{x^2-y^2}+id_{xy}$ type orbital at the first 2b position $(\frac{3}{4},\frac{1}{4},0)$. Note that $\mathrm{A_c''}\uparrow\mathrm{G}$ is the only decomposable MEBR, although bands with this MEBR are always connected in this work. One should notice that we use a different convention of the origin point as the Bilbao Crystallographic Server, as explained in the second paragraph in Supplementary Sec. III A.

difference larger than $\Lambda_2$. Therefore, the actual Hamiltonian with finite $\Lambda_{1,2}$ can be written as

$$
\begin{aligned}
H_{N,0} =& \sum_{k_x,k_y}\sum_{\alpha}\sum_{|G_x|<\Lambda_1} \alpha v(k_x+G_x)\phi_h^\dagger(k_x,k_y;\alpha,G_x)\phi_h(k_x,k_y;\alpha,G_x) \\
&+\sum_{k_x,k_y}\sum_{\alpha}\sum_{|G_y|<\Lambda_1} \alpha v(k_y+G_y)\phi_v^\dagger(k_x,k_y;\alpha,G_y)\phi_v(k_x,k_y;\alpha,G_y) \\
H_{N,1} =& \frac{\lambda}{2a}\sum_{k_x,k_y}\sum_{\alpha,\beta}\sum_{|G_x|<\Lambda_1}\sum_{|G_y|<\Lambda_1} \Xi_{\Lambda_2}(\alpha(k_x+G_x)-\beta(k_y+G_y))\exp\left[i(\frac{1+\alpha}{2})G_ya - i(\frac{1+\beta}{2})G_xa\right] \\
&\times \phi_h^\dagger(k_x,k_y;\alpha,G_x)\phi_v(k_x,k_y;\beta,G_y) + h.c.
\end{aligned}
\tag{36}
$$

where $\Xi_{\Lambda_2}$ is a hard-cutoff factor

$$
\Xi_{\Lambda_2}(\tau)=\begin{cases} 0 & (|\tau|>\Lambda_2) \\ 1 & (|\tau|<\Lambda_2) \\ \frac{1}{2} & (|\tau|=\Lambda_2) \end{cases}. \tag{37}
$$

In all the calculations, we choose $\Lambda_1 \gg \Lambda_2$ such that the degrees of freedom on a chiral wire inside the $\delta$ potential region ($\sim 1/\Lambda_2$) can be treated as continuous. We leave further discussions on $\Lambda_{1,2}$ to Supplementary Sec. III C.

The band structures and the corresponding irreps (irreducible representations) of the middle ten bands with $v=a=1$, $\Lambda_1=50\pi$, $\Lambda_2=10\pi$, and various $\theta=\lambda/v$'s are shown in Supplementary Fig. 18 Irreps are defined in Supplementary Table I. The degeneracy at the X point comes from the magnetic translation $\widetilde{T}=\{1'|1/2,1/2,0\}$ (here the length unit is lattice constant 2a), which squares to $\{1|1,1,0\}=-1$ at $X$ and hence protects Kramers' pairs. One may notice the periodicity in energy - the only difference between the nth and the n + 8th band is a constant energy shift (the red panes in Supplementary Fig. 18 indicate the repeating unit). We will prove this periodicity at the end of this subsection. Nevertheless, we should note that this periodicity is not essential for the main conclusion of our work (the appearance of critical metal phase). In fact, we build 8-band lattice models in later sections, and they also have critical metal phases as the network model.

Now let us analyze the representations of the band structure. We take the upper eight of the ten bands as the repeating unit in energy and focus on it. Then we notice that the band structure comprises disconnected branches, each of them containing two

bands. One branch forms one of the following four MEBRs [7] (magnetic element band representations) defined in Supplementary Table II: $\mathrm{A_b}\!\uparrow\!\mathrm{G}$, $\mathrm{B_b}\!\uparrow\!\mathrm{G}$, ${}^1\mathrm{E_b}\!\uparrow\!\mathrm{G}$, ${}^2\mathrm{E_b}\!\uparrow\!\mathrm{G}$. One MEBR is the minimal trivial group of bands formed by symmetric local orbitals in real space, and the left part of a MEBR notation indicates the site symmetry representation of orbitals. These four MEBRs ($\mathrm{A_b}\!\uparrow\!\mathrm{G}$, $\mathrm{B_b}\!\uparrow\!\mathrm{G}$, ${}^1\mathrm{E_b}\!\uparrow\!\mathrm{G}$, ${}^2\mathrm{E_b}\!\uparrow\!\mathrm{G}$) are formed by effective $s$, $d_{x^2-y^2}+id_{xy}$ (or equivalently $d_{x^2-y^2}-id_{xy}$), $p_x-ip_y$, $p_x+ip_y$ orbitals, respectively. All these orbitals center at the Wyckoff position $2\mathrm{b}$ $((\frac{3}{4}\frac{1}{4}0)$ and $(\frac{1}{4}\frac{3}{4}0)$ ). Since $2\mathrm{b}$ has multiplicity 2 (two $C_{4z}$ centers per unit cell), each MEBR contains two bands. The transition between two localized limits at $\theta=\pm\pi/2$ manifests in the transitions of MEBRs. When $\lambda<0$ ( $\theta<0$), the bands are gapped and the representation of the lower four bands in one repeating window is a direct sum of two MEBRs: ${}^2\mathrm{E_b}\!\uparrow\!\mathrm{G}\oplus\mathrm{A_b}\!\uparrow\!\mathrm{G}$. When $\lambda=0$ ($\theta=0$), the gaps close and the band structure is indeed that of ballistic 1D metals with linear dispersion relations. As $\lambda$ increases across $0$, if one focus on the energy level indicated by the gray dashed lines in Supplementary Fig. 18 (the middle of the chosen window), one will find that irreps $\Gamma_1$, $M_1$ ($\Gamma_2$, $M_2$) go up (down) across the energy level. Similar irrep exchanges also happen beyond and below the window. After the transition ($\lambda>0$), the gaps reopen and the MEBRs of the lower four bands in one repeating window change to ${}^1\mathrm{E_b}\!\uparrow\!\mathrm{G}\oplus\mathrm{B_b}\!\uparrow\!\mathrm{G}$. The transition is reversed for the upper four bands. Hence, $\theta=\pm\pi/2$ corresponds to two different trivial phases.

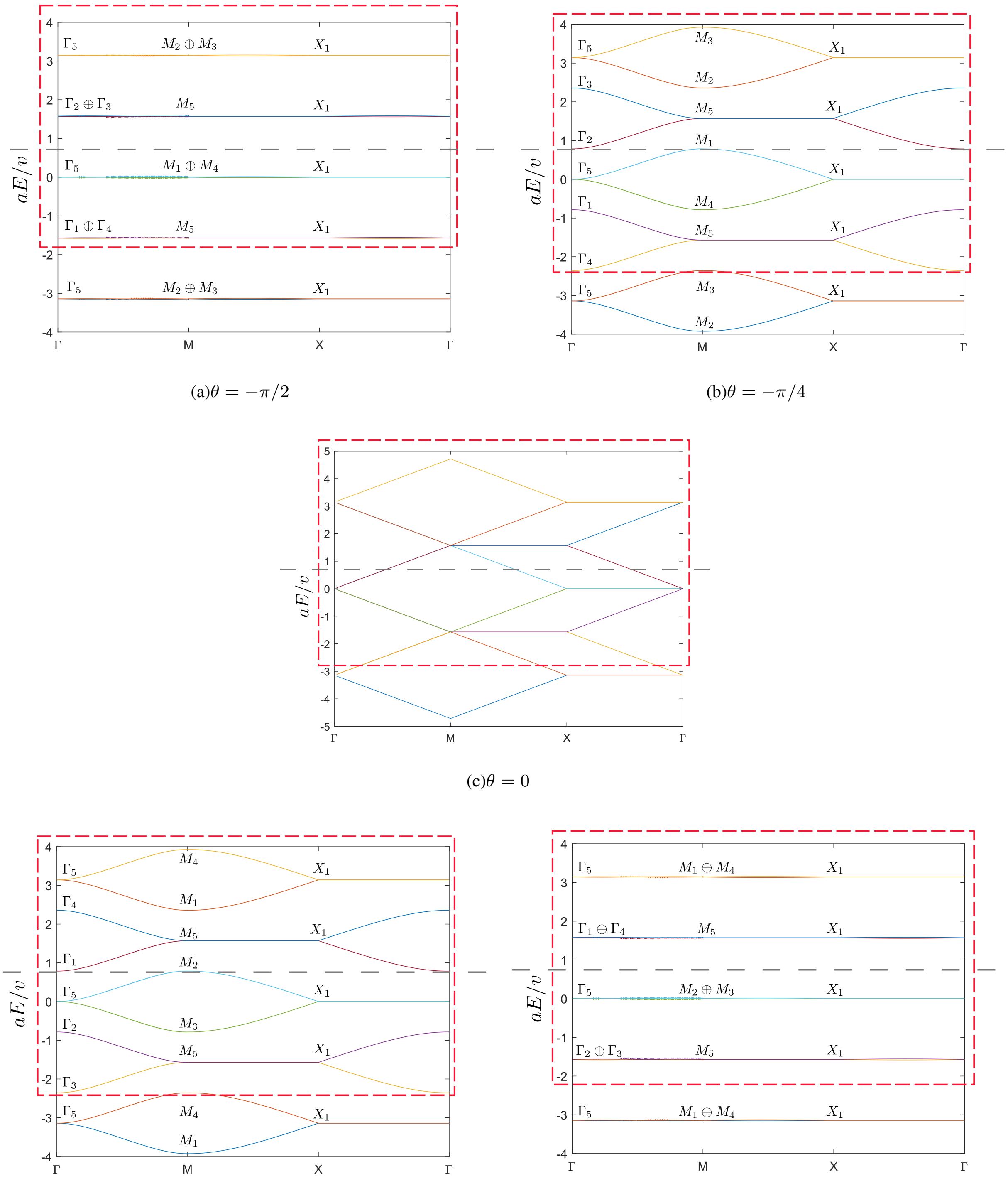


(a)$\theta = -\pi/2$ (b)$\theta = -\pi/4$

(c)$\theta = 0$

(d)$\theta = \pi/4$ (e)$\theta = \pi/2$

Supplementary Fig. 18. Band representations and structures of the network model with different scattering strengths ($\Lambda_1 = 50\pi/a$, $\Lambda_2 = 10\pi/a$). Panel (a) with $\theta = -\pi/2$ corresponds to the first insulated limit. Panel (b) with $\theta = -\pi/4$ happens to close the indirect gap and hence represents the intermediate state between insulated and conductive phases. Panel (c) with $\theta = 0$ corresponds to the ballistic limit comprising decoupled 1D chiral wires. Panels (d) and (e) are similar to (b) and (a) but in another insulated limit. The red dashed panes indicate the repeating units of the band structures. The gray dashed lines indicate the Fermi level used in later sections. Note that the corresponding main text plots, i.e., Fig. 1(g), (h), (i), take $\pi v/4a$ here as the zero energy. Definitions of irreps at high symmetry points can be found in Supplementary Table I.

The band structure periodicity in energy can be understood heuristically by the locality of $H_{N,1}$. For a given wave vector $\vec{k}$, we take the eigenstates of $H_{N,0}(k_x, k_y)$ as a basis $\{|\phi_d(k_x, k_y; \alpha, G)\rangle \,|d = h, v,\ \alpha = \pm 1,\ G = \mathbb{Z}\pi/a\}$. Since $H_{N,1}$ corresponds to $\delta$-scattering potentials at square corners, it can only perceive the phases (of states) at square corners. According to Eq. (32), $|\phi_d(k_x, k_y; \alpha, G)\rangle$ has the same phases as $|\phi_d(k_x, k_y; \alpha, G + 2\mathbb{Z}\pi/a)\rangle$ at square corners. Therefore, the scattering amplitude (caused by $H_{N,1}$) between $|\phi_d(k_x, k_y; \alpha, G)\rangle$ & $|\phi_{d'}(k_x, k_y; \alpha', G')\rangle$ is the same as that of $|\phi_d(k_x, k_y; \alpha, G + 2\pi/a)\rangle$ & $|\phi_{d'}(k_x, k_y; \alpha', G' + 2\pi/a)\rangle$. The only difference between these two pairs is an overall energy shift $2\pi v/a$ caused by $H_{N,0}$. Hence, the band structure should have a period of $2\pi v/a$ in energy. Notice that the spectrum of $H_{N,0}$ is continuous and there are eight bands between $E = vG$ and $vG + 2\pi v/a$ correspond to $\{\phi_h(k_x, k_y; +, G),\ \phi_h(k_x, k_y; -, -G),\ \phi_h(k_x, k_y; +, G + \pi v/a),\ \phi_h(k_x, k_y; -, -G - \pi v/a);\ h \leftrightarrow v\}$. (We ignore the dispersion energy $\alpha v k_{x/y}$ for clarity since the energy order of bands only depends on $\alpha G$ when $\vec{k}$ is restricted to the first BZ.) The scattering induced by $H_{N,1}$ will open gaps among the connected bands of $H_{N,0}$, so there are still eight bands between $E = vG$ and $vG + 2\pi v/a$. Therefore, the band structure of $H_{N,0} + H_{N,1}$ has an energy period of $2\pi v/a$, and each period contains eight bands.

To prove the periodicity more quantitatively, we introduce the unitary transformation (we ignore $\Lambda_1$, $\Lambda_2$ or take $\Lambda_1$, $\Lambda_2 \to \infty$ in this proof)

$$U\phi_h(k_x, k_y; \alpha, G_x)U^\dagger = \phi_h(k_x, k_y; \alpha, G_x - \alpha 2\pi/a), \qquad U\phi_v(k_x, k_y; \alpha, G_x)U^\dagger = \phi_v(k_x, k_y; \alpha, G_y - \alpha 2\pi/a)\ . \tag{38}$$

Or in the representation of the invariant subspace spanned by $\left\{\left|\phi_{h/v}(k_x, k_y; \alpha, G)\right\rangle |G \in \mathbb{Z}\pi/a,\ \alpha = \pm 1\right\}$

$$(U(k_x, k_y; h/v))_{\alpha G, \alpha' G'} = \delta_{\alpha,\alpha'}\delta_{G, G' - \alpha 2\pi/a} \tag{39}$$

It is direct to verify that, when the cutoff $\Lambda_1$ is infinity, $U$ transforms $H_{N,0}$ to itself plus a density term

$$\begin{aligned} UH_{N,0}U^\dagger =& H_{N,0} + 2\pi\frac{v}{a}\sum_{k_x,k_y,\alpha}\sum_{G}\Big(\phi_v^\dagger(k_x, k_y; \alpha, G)\phi_v(k_x, k_y; \alpha, G) + \phi_h^\dagger(k_x, k_y; \alpha, G)\phi_h(k_x, k_y; \alpha, G)\Big) \\ =& H_{N,0} + 2\pi\frac{v}{a}\hat{N} \end{aligned} \tag{40}$$

with $\hat{N}$ being the particle number operator. One can also verify that

$$UH_{N,1}U^\dagger = H_{N,1}\ . \tag{41}$$

Notice that the momentum shifted by $U$ is chosen as twice the minimal reciprocal lattice length, *i.e.*, $2 \times 2\pi/(2a)$, such that the phase factors in $H_{N,1}$ are invariant under the transformation $G_x \to G_x - \alpha\frac{2\pi}{a}$, $G_y \to G_y - \beta\frac{2\pi}{a}$, *i.e.*,

$$\begin{aligned} &\Xi_{\Lambda_2}(\alpha(k_x + G_x) - \beta(k_y + G_y))\exp\left[i(\frac{1+\alpha}{2})G_y a - i(\frac{1+\beta}{2})G_x a\right] \\ \to& \Xi_{\Lambda_2}(\alpha(k_x + G_x - \alpha 2\pi/a) - \beta(k_y + G_y - \beta 2\pi/a))\exp\left[i(\frac{1+\alpha}{2})(G_y - \beta 2\pi/a)a - i(\frac{1+\beta}{2})(G_x - \alpha 2\pi/a)a\right] \\ =& \Xi_{\Lambda_2}(\alpha(k_x + G_x) - \beta(k_y + G_y))\exp\left[i(\frac{1+\alpha}{2})G_y a - i(\frac{1+\beta}{2})G_x a\right]\ . \end{aligned} \tag{42}$$

In summary there is

$$U(H_{N,0} + H_{N,1})U^\dagger = H_{N,0} + H_{N,1} + \frac{2\pi v}{a}\hat{N}\ . \tag{43}$$

Suppose $|\psi_{\vec{k},n}\rangle$ is a single-particle eigenstate of $H_{N,0} + H_{N,1}$ with the energy $E_n(\vec{k})$, then $U^\dagger|\psi_{\vec{k},n}\rangle$ is a single-particle state with the energy $E_n(\vec{k}) + \frac{2\pi v}{a}$. Therefore, we have proven the energy periodicity.

Now, we argue that every energy window $[E, E + \frac{2\pi v}{a})$ contains eight bands by counting the Hilbert space dimension. Suppose there are $N_G$ number of $G$'s satisfying $|G| < \Lambda_1$. Then the Hilbert space dimension at each $\vec{k}$ is $4N_G$ since for each $G$ there are $\alpha = \pm 1$ horizontal and vertical modes, *i.e.*, $\phi_h(k_x, k_y; \alpha, G)$ and $\phi_v(k_x, k_y; \alpha, G)$ ($\alpha = \pm 1$). As $U$ shifts $G$ by twice the minimal reciprocal lattice, $U$ can act in the Hilbert space at most $\frac{N_G}{2}$ times. To be particular, $U^{\frac{N_G}{2}}$ sends the largest negative $G$ to the largest postive $G$. Therefore, $U$ divides the total Hilbert space into $N_G/2$ pieces. If we act $U$ on the energy bands, it will yield $N_G/2$ groups, with every group $4N_G/(N_G/2) = 8$ bands. Nearby groups have an energy difference of $2\pi v/a$ according to the last paragraph. Thus, eight nearby bands form the smallest repeating unit in energy.

In practice, finite cutoff factors $\Lambda_1$, $\Lambda_2$ will be introduced. The above argument, in fact, fails when applied to high-energy bands close to the cutoff. Nevertheless, when the cutoff is sufficiently large, it applies to low-energy states far away from the cutoff.

### C. Further discussions on the cutoff

The effect of $\Lambda_1$ is straightforward: it discretizes the in-line coordinates of chiral wires, *i.e.*, the $x$ in $\psi_h(x;m,\alpha)$ and $y$ in $\psi_v(y;n,\alpha)$. We denote the granularity as $b\sim\frac{1}{\Lambda_1}$.

To see the role of $\Lambda_2$, we begin with the scattering potential with a (hard) cutoff in reciprocal space and ignore $\Lambda_1$ temporarily

$$\begin{aligned}H_{N,1}=&\sum_{\vec{k}}\sum_{G_xG_y}\sum_{\alpha\beta}\frac{\lambda}{2a}\,\Xi_{\Lambda_2}\left[\alpha(k_x+G_x)-\beta(k_y+G_y)\right]\\&\times\exp\left[i(\frac{1+\alpha}{2})G_ya-i(\frac{1+\beta}{2})G_xa\right]\phi_h^\dagger(k_x,k_y;\alpha,G_x)\phi_v(k_x,k_y;\beta,G_y)+h.c.\end{aligned}\tag{44}$$

The cutoff factor $\Xi_{\Lambda_2}$ is defined in Eq. (37). Then we carry out the Fourier transform Eq. (32):

$$\begin{aligned}H_{N,1}=&\sum_{nm\alpha\beta}\iint\mathrm{d}x\mathrm{d}y\,\delta[\alpha(x-2na-\frac{1+\beta}{2}a)-\beta(y-2ma-\frac{1+\alpha}{2}a)]\\&\times\frac{\lambda\sin\left[\Lambda_2(x-2na-\frac{1+\beta}{2}a)\right]}{\pi(x-2na-\frac{1+\beta}{2}a)}\psi_h^\dagger(x;m\alpha)\psi_v(y;n\beta)+h.c.\end{aligned}\tag{45}$$

We obtain a broadened $\delta$-scattering potential in the form of $sinc$ function. Other (hard or soft) momentum cutoff factors will result in different broadening profiles. Regardless of the concrete formula of cutoff factor, $\Lambda_2$ always characterizes the width $\zeta\sim\frac{1}{\Lambda_2}$ of broadened $\delta$-potential.

Now let us reinspect Eq. (26). The derivation of Eq. (26) includes an integration of Eq. (24) near zero ($\int_{0^-}^{0^+}$). Since the generalized function $\delta(\xi)$ is usually understood as the limit of some ordinary function sequence, integration of Eq. (24) should be understood in this way:

$$\lim_{\xi_0\to0^+}\int_{-\xi_0}^{\xi_0}\mathrm{d}\xi\left[-iv\frac{\mathrm{d}\ln\psi_\pm(\xi)}{\mathrm{d}\xi}\right]=\lim_{\xi_0\to0^+}\left\{\lim_{\zeta\to0^+}\int_{-\xi_0}^{\xi_0}\mathrm{d}\xi\left[E\mp\lambda\delta_\zeta(\xi)\right]\right\}\tag{46}$$

where $\delta_\zeta$ is a broadened $\delta$ function with characteristic length $\zeta\sim\frac{1}{\Lambda_2}$. The order of limits on the right side of Eq. (46) tells us that $\zeta\ll\xi_0$, since it takes $\zeta\to0$ first and then $\xi_0\to0$. We should further notice that, when taking these two limits, $\xi$ is viewed as a continuous integral variable, *i.e.*, the granularity of $\xi$ is much smaller than $\zeta$. Hence, we have the hierarchy $b\ll\zeta\ll\xi_0$ ($\Lambda_2\ll\Lambda_1$). Therefore, if we take $\Lambda_2\geq\Lambda_1$, Eq. (26) is invalid.

We can now understand why introducing $\Lambda_2$ is necessary. If we only take a cutoff $\Lambda_1$ on $G_{x,y}$, then effectively $\Lambda_2\to\infty$. In this case, Eq. (26) is invalid. Physically, it is equivalent to discretizing the chiral wires without broadening the $\delta$-potentials.

The above analysis informs us that although Eq. (26) is an inevitable result from the perspective of the differential equation, the situation is subtle in the numerical calculation that involves $\Lambda_{1,2}$. More concretely, we should distinguish two cases of $b$ versus $\zeta$, *i.e.*, $b\ll\zeta$ ($\Lambda_1\gg\Lambda_2$) and $b\geq\zeta$ ($\Lambda_1\leq\Lambda_2$). To validate Eq. (26), we should take the former case. Nevertheless, it is beneficial to show how such two cases are different. In fact, these two cases of $b$ vs. $\zeta$ correspond to two system sequences (Supplementary Fig. 19) that both take a $\delta$-potential on a continuous chiral wire (Supplementary Fig. 17) as their limitations. However, such two system sequences lead to different scattering results.

When $b\ll\zeta$, *i.e.*, $b$ is much smaller than any scale, $\xi$ can be viewed as a continuous variable, and the scattering process can be effectively depicted by the system sequence shown in Supplementary Fig. 19(a). In Supplementary Fig. 19(a), we set continuous wires & ordinary-function-type scattering potential first and then shrinks the potential. (We should mention that the ordinary function sequence is not limited to $\{\frac{1}{\pi}\sin\frac{\mu\xi}{\xi}|\mu\to\infty\}$ shown in Supplementary Fig. 19(a), any sequence of ordinary functions that converges to $\delta(\xi)$ is allowed.) This system sequence validates Eq. (26). And if we integrate Eq. (23) near zero, we will obtain

$$\begin{aligned}\int_{0^-}^{0^+}d\xi E\psi_\pm(\xi)=&\int_{0^-}^{0^+}d\xi[-iv\frac{d}{d\xi}\pm\lambda\delta(\xi)]\psi_\pm(\xi)\\0=&-iv[\psi_\pm(0^+)-\psi_\pm(0^-)]\pm\lambda\int_{0^-}^{0^+}\delta(\xi)\psi_\pm(\xi)\end{aligned}\tag{47}$$

Since now Eq. (24) is valid, we can use its corollary $\psi_\pm(0^+) = e^{\mp i\lambda/v}\psi_\pm(0^-)$, then we have

$$\begin{aligned}\int_{0^-}^{0^+} \mathrm{d}\xi\, \delta(\xi)\psi_\pm(\xi) &= \pm i\frac{v}{\lambda}(e^{\mp i\lambda/2v} - e^{\pm i\lambda/2v})\sqrt{\psi_\pm(0^-)\psi_\pm(0^+)} \\ &= \frac{\sin(\lambda/2v)}{(\lambda/2v)}\sqrt{\psi_\pm(0^-)\psi_\pm(0^+)}.\end{aligned} \tag{48}$$

For the second sequence (Supplementary Fig. 19(b)), we first discretize the chiral wire (which results in a discrete wire with infinite-long range hopping), then add one-point exchange hopping between two discretized chiral wires, and finally take the granularity of the discretized wires to zero. Hence, the second sequence corresponds to $b \geq \zeta$ and has a different integral result from Eq. (48):

$$\int_{0^-}^{0^+} \mathrm{d}\xi\, \delta(\xi)\psi_\pm(\xi) = \frac{\psi_\pm(0^+) + \psi_\pm(0^-)}{2}. \tag{49}$$

which can be numerically confirmed by calculating a single node system defined in Supplementary Fig. 19(b). Combining Eq. (47)and Eq. (49), the scattering result of the second sequence is

$$\begin{pmatrix}\psi_1(0^+)\\ \psi_2(0^+)\end{pmatrix} = \begin{pmatrix}\frac{1-\widetilde{\lambda}^2}{1+\widetilde{\lambda}^2} & \frac{-2i\widetilde{\lambda}}{1+\widetilde{\lambda}^2}\\ \frac{-2i\widetilde{\lambda}}{1+\widetilde{\lambda}^2} & \frac{1-\widetilde{\lambda}^2}{1+\widetilde{\lambda}^2}\end{pmatrix}\begin{pmatrix}\psi_1(0^-)\\ \psi_2(0^-)\end{pmatrix} \qquad \widetilde{\lambda} = \frac{\lambda}{2v}, \tag{50}$$

which can reproduce Eq. (19) when $\lambda \in [-2v, 2v]$. However, Eq. (50) lacks the periodicity on $\lambda$ compared to Eq. (26). More importantly, the second sequence discretizes chiral wires, which results in infinite-long-range hopping on each discretized wire. In Sec. IV A, we need to redistribute degrees of freedom in the crossed two wires into two corners (see Supplementary Fig. 21(b)). For the second sequence, such redistribution will result in infinite-long-range hopping between corners (rather than inside each corner). This feature of the second sequence make the situation complicated. On the other hand, the first sequence does not discretize chiral wires and has no such a problem. Therefore, we take the first sequence ($b \ll \zeta$) in this section and use it for constructing the lattice models in Supplementary Sec. IV.

In terms of numerical program, where we first have discrete wires, the first sequence corresponds to $\Lambda_1, \Lambda_2 \to \infty$, $\Lambda_2/\Lambda_1 \to 0$ ($\zeta, b \to 0, \zeta/b \to \infty$), the second case corresponds to $\Lambda_1, \Lambda_2 \to \infty$, $\Lambda_2 \geq \Lambda_1$ ($\zeta, b \to 0, \zeta \leq b$). Numerical works on the band structure of $H_{N,0} + H_{N,1}$ proved the above analysis. When $\Lambda_1, \Lambda_2 > 10\,\&\,\Lambda_2/\Lambda_1 \leq 1/2$, the bands are flat at $\lambda = (\mathbb{Z} + 1/2)\pi v$ and are quasi-1D linearly dispersed at $\lambda = \mathbb{Z}\pi v$ (see Supplementary Fig. 20(a) & 20(c)). This corresponds to Supplementary Fig. 19(a) which subjects to Eq. (26). Since Eq. (26) implies that the eigenstates are decoupled local currents when $\lambda = (\mathbb{Z} + 1/2)\pi v$ and are decoupled horizontal & vertical wires when $\lambda = \mathbb{Z}\pi v$. When $\Lambda_1, \Lambda_2 > 10\,\&\,\Lambda_2/\Lambda_1 \geq 2$, the bands are flat at $\lambda/v = \pm 2$ (see Supplementary Fig. 20(e)) and are quasi-1D linearly dispersed at $\lambda = 0, \pm\infty$ (see Supplementary Fig. 20(f)). This corresponds to Supplementary Fig. 19(b) which subjects to Eq. (50), since Eq. (50) implies that the eigenstates are decoupled local currents when $\lambda = \pm 2v$ and are decoupled horizontal & vertical wires when $\lambda = 0, \pm\infty$.

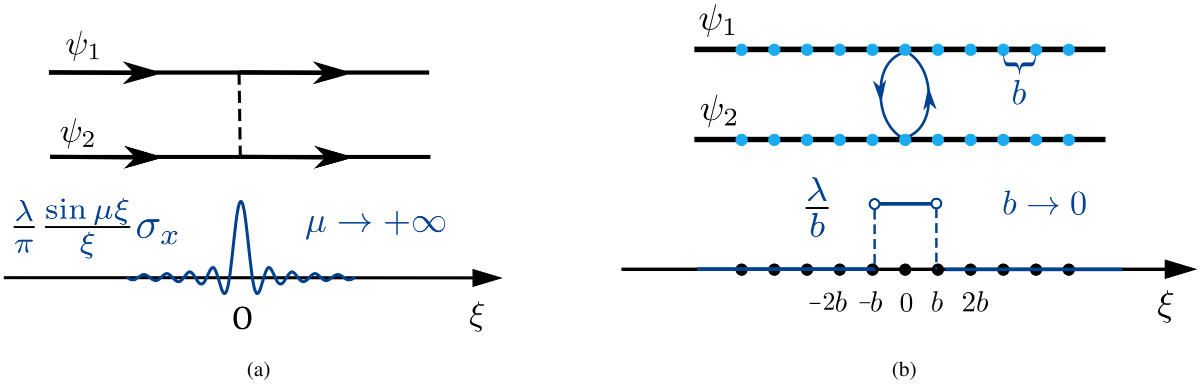


Supplementary Fig. 19. Two methods to approach a $\delta$ scattering problem. (a) Continuous wire with shrinking scattering potential. (b) One-point scattering hopping between discrete wires that are more and more fine-grained.

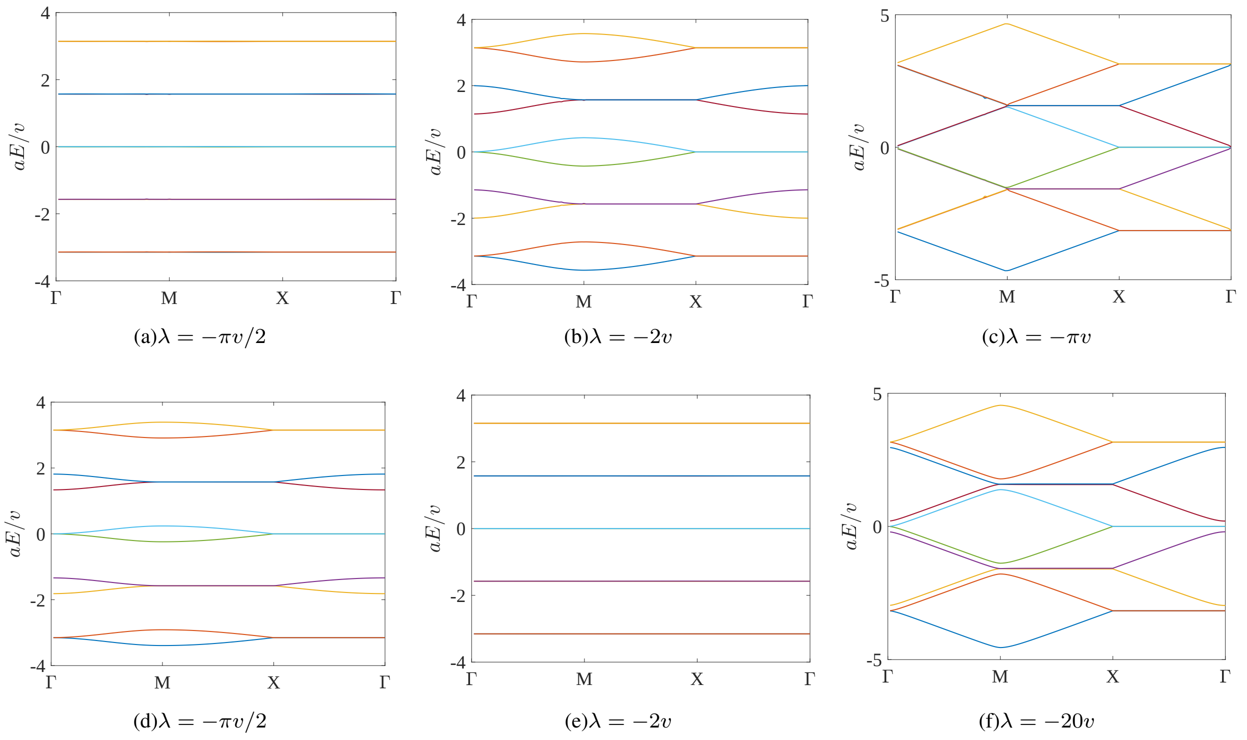


Supplementary Fig. 20. Band structures of Eq. (36) with $\Lambda_1 = 50\pi/a$, $\Lambda_2 = 25\pi/a$ for (a) $\sim$ (c) and $\Lambda_1 = 50\pi/a$, $\Lambda_2 = 100\pi/a$ for (d) $\sim$ (f). (a) $\sim$ (c) correspond to Supplementary Fig. 19(a), which subjects to Eq. (26). According to Eq. (26), the eigenstates are decoupled local currents at $\lambda = (\mathbb{Z} + \frac{1}{2})\pi v$ and are decoupled horizontal & vertical wires at $\lambda = \mathbb{Z}\pi v$. These predictions are confirmed by (a) and (c), respectively. (d) $\sim$ (f) correspond to Supplementary Fig. 19(b), which subjects to Eq. (50). According to Eq. (50), the eigenstates are decoupled local currents at $\lambda = \pm 2v$ and are decoupled horizontal & vertical wires at $\lambda = 0, \pm\infty$. These are consistent with (d) $\sim$ (f). Though not illustrated in this figure, it is obvious that the dispersion relations for these two systems at $\lambda = 0$ are quasi-1D linear as (c), since it is just $H_{N,0}$.

## IV. Mapping the network model to lattice models

### A. Un-truncated lattice model: $H_N$ on standing wave basis

The network model was introduced through the wire construction; we can equivalently rewrite it in a local orbital basis. Without loss of generality, we can choose the basis as the local current loop states in the limit $\theta = -\pi/2$ discussed in Supplementary Sec. III A, *i.e.*, standing waves going around the red/blue squares. We emphasize that the existence of the local current loop basis means our model has no symmetry anomaly associated to the crystalline symmetries or the accidental particle-hole symmetry discussed in Supplementary Sec. III B. (Notice that the accidental particle-hole symmetry will be broken when the disorder is considered.)

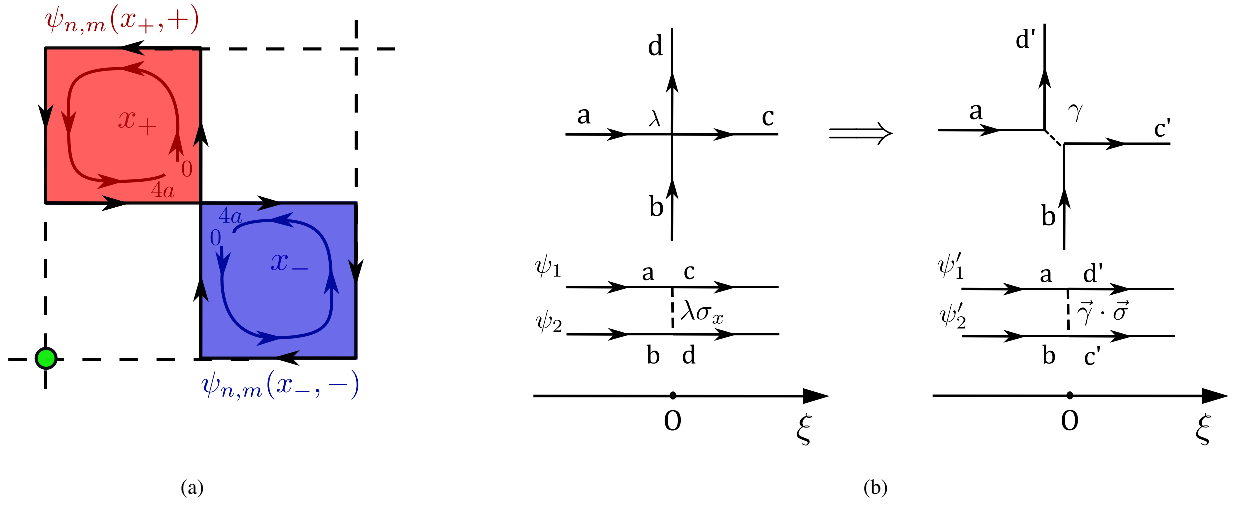


Supplementary Fig. 21. Unit cell and scattering potential of un-truncated lattice model. (a) Unit cell and its coordinate convention. The green dot indicates the origin of unit cell used in the network model. One should distinguish it from the origin of coordinates $x_\pm$. (b). Mapping of a scattering node (left: network, right: lattice). $\lambda$ and $\vec{\gamma}$ are constants represent the scattering strengths. The basis of the $\sigma_x$ on the left is $\psi_1, \psi_2$ and that of the $\vec{\sigma}$ on the right is $\psi'_1, \psi'_2$

In order to obtain the standing wave representation, we first introduce the circular chiral states $\psi_{n,m}(x_\alpha, \alpha)$ going around Chern blocks centered at $(2n+1-\frac{\alpha}{2}, 2m+1+\frac{\alpha}{2})a$, as shown in Supplementary Fig. 21(a). Here $\alpha$ represents the Chern number of the associated Chern block, and $x_\alpha \in [0, 4a)$ is a local coordinate of the circular chiral states, which goes in the anti-clockwise directions for both $\alpha = +$ and $-$ blocks. One should not confuse $x_\alpha$ here with the global coordinate $(x, y)$ defined in Supplementary Sec. III B. By definition, there is $\psi_{n,m}(x_\alpha, \alpha) = \psi_{n,m}(x_\alpha + 4a, \alpha)$. We also remind the reader to distinguish $\psi_{n,m}$ from the fields in the wire construction $\psi_{h/v}$. The fields $\psi_{n,m}(x_\alpha, \alpha)$ are basically re-combinations of the vertical and horizontal wires: on each edge of a Chern block, $\psi_{n,m}(x_\alpha, \alpha)$ equals to some $\psi_h$ or $\psi_v$. For instance, $\{\psi_{n,m}(x_+, +)|x_+ \in [0, a)\}$ equals to $\{\psi_v(y; n, +)|y \in [(2m+1)a, (2m+2)a)\}$. However, one must be careful about how different segments of $\psi_h$ and $\psi_v$ are connected in $\psi_{n,m}(x_\alpha, \alpha)$.

To see the connection of different segments, we consider a scattering potential $v\theta\sigma_x$ between two chiral modes $\psi_1$ and $\psi_2$ (which is also the basis of $\sigma_x$), as shown on the left side of Supplementary Fig. 21(b). Here $v$ is the velocity of the chiral modes. As established in Supplementary Sec. III B, $\theta$ is the scattering angle, *i.e.*, $\psi_c = \cos\theta\psi_a - i\sin\theta\psi_d$, $\psi_d = \cos\theta\psi_b - i\sin\theta\psi_a$. ($\psi_a = \psi_1(0^-)$, $\psi_b = \psi_2(0^-)$, $\psi_c = \psi_1(0^+)$, $\psi_d = \psi_2(0^+)$.) In order to investigate the scattering potential in circular chiral state basis $\psi_{n,m}$, we need to recombine $\psi_{1,2}$ to $\psi'_{1,2}$ (see the right side of Supplementary Fig. 21(b)) such that

$$\psi'_1(\xi) = \psi_1(\xi), \qquad \psi'_2(\xi) = \psi_2(\xi), \qquad (\xi < -\xi_0) \tag{51}$$

$$\psi'_1(\xi) \propto \psi_2(\xi), \qquad \psi'_2(\xi) \propto \psi_1(\xi), \qquad (\xi > \xi_0) \tag{52}$$

where $\xi_0 \ll a$ is a small positive quantity. In other words, $\psi_1$ and $\psi_2$ are interchanged (up to some phase factors) on the "right

side" of the $\delta$-potential. To realize such an interchange, we use a smooth transformation

$$\begin{aligned}
&\begin{pmatrix}\psi_1'(\xi)\\ \psi_2'(\xi)\end{pmatrix} = \begin{pmatrix}\cos\Phi(\xi) & -i\sin\Phi(\xi)\\ -i\sin\Phi(\xi) & \cos\Phi(\xi)\end{pmatrix}\begin{pmatrix}\psi_1(\xi)\\ \psi_2(\xi)\end{pmatrix}\\
&\Phi(\xi) = \begin{cases} 0 \quad (\xi \le -\xi_0) \\ \text{quickly and monotonicly} \\ \text{becomes } (2n+1/2)\pi & (-\xi_0 < \xi \le \xi_0) \\ (2n+1/2)\pi \quad (\xi > \xi_0)\end{cases}
\end{aligned} \tag{53}$$

where $n$ is an arbitrary integer. (Now we keep $n$ for generality. Nevertheless, we will later show that $n$ has no effect and can be set as 0.) From the perspective of probability conservation, both $-i\sin\Phi$ and $i\sin\Phi$ are permitted. But since we choose the eigenstates of the network model at $\theta = -\pi/2$ as the basis of desired lattice model, and these eigenstates have phase jumps $i$ at corners ($\psi_{1,2}(0^+) = i\psi_{2,1}(0^-)$), we should choose $-i\sin\Phi$ to remove the phase jump in the representation of $\psi'_{1,2}$. More explicitly, taking $-i\sin\Phi$ means $\psi'_{1,2}(0^+) = -i\psi_{2,1}(0^+)$, $\psi'_{1,2}(0^-) = \psi_{1,2}(0^-)$. The eigenstates of $\theta = -\pi/2$ satisfy $\psi_{1,2}(0^+) = i\psi_{2,1}(0^-)$. Combine these together, we have $\psi'_{1,2}(0^+) = -i\psi_{2,1}(0^+) = \psi_{1,2}(0^-) = \psi'_{1,2}(0^-)$, *i.e.*, the eigenstats at $\theta = -\pi/2$ are smooth functions of $\xi$ in the representation of $\psi'_{1,2}$.

As shown in the next equation, the transformed Hamiltonian has the same form as the original one but has a different scattering angle. The scattering Hamiltonian in terms of $\psi'_{1,2}$ reads

$$\begin{aligned}
&\begin{pmatrix}\psi_1^\dagger(\xi) & \psi_2^\dagger(\xi)\end{pmatrix}(-iv\partial_\xi\sigma_0 + v\theta\delta(\xi)\sigma_x)\begin{pmatrix}\psi_1(\xi)\\ \psi_2(\xi)\end{pmatrix}\\
=&\begin{pmatrix}\psi'^\dagger_1(\xi) & \psi'^\dagger_2(\xi)\end{pmatrix}\begin{pmatrix}\cos\Phi(\xi) & -i\sin\Phi(\xi)\\ -i\sin\Phi(\xi) & \cos\Phi(\xi)\end{pmatrix}(-iv\partial_\xi\sigma_0 + v\theta\delta(\xi)\sigma_x)\begin{pmatrix}\cos\Phi(\xi) & i\sin\Phi(\xi)\\ i\sin\Phi(\xi) & \cos\Phi(\xi)\end{pmatrix}\begin{pmatrix}\psi_1'(\xi)\\ \psi_2'(\xi)\end{pmatrix}\\
=&\begin{pmatrix}\psi'^\dagger_1(\xi) & \psi'^\dagger_2(\xi)\end{pmatrix}[-iv\partial_\xi\sigma_0 + v\partial_\xi\Phi(\xi)\sigma_x + v\theta\delta(\xi)\sigma_x]\begin{pmatrix}\psi_1'(\xi)\\ \psi_2'(\xi)\end{pmatrix}\\
=&\begin{pmatrix}\psi'^\dagger_1(\xi) & \psi'^\dagger_2(\xi)\end{pmatrix}[-iv\partial_\xi\sigma_0 + v(\theta + 2n\pi + \pi/2)\delta(\xi)\sigma_x]\begin{pmatrix}\psi_1'(\xi)\\ \psi_2'(\xi)\end{pmatrix}
\end{aligned} \tag{54}$$

We made use of the steep growth of $\Phi(\xi)$ in the last equation, *i.e.*, $\partial_\xi\Phi(\xi) = (2n\pi + \pi/2)\delta(\xi)$. The result of the transformation is a shift of the scattering angle: $\theta \to \theta + \pi/2 + 2\pi n$. One should notice that the scattering problem in terms of $\psi'_{1,2}$ is similar to that of $\psi_{1,2}$. Hence, the scattering effect is also periodic on the potential strength, and the ambiguity of integer $n$ does not make any difference. We take $n = 0$ hereafter.

The transformed scattering potential can be understood in some limits. The local orbital basis $\mathcal{B}$ we use in this lattice model comprises the eigenstates of the network model at $\theta = -\pi/2$. Therefore, there must be no scattering between the local orbitals when $\theta = -\pi/2$, *i.e.*, the scattering potential in the representation of $\mathcal{B}$ should be 0. When $\theta = \pi/2$, the eigenstates are also local currents, but the phase jumps are reversed (see 19, when $\theta = \pm\pi/2$, the phase jump is $\mp i$ from the incoming to the outgoing channel). Effectively, in the representation of $\mathcal{B}$, these eigenstates are local currents with phase jumps $-1$ at corners. Hence the effective scattering angle in terms of $\mathcal{B}$ should be $\pi$. When $\theta = 0$, the eigenstates are decoupled horizontal and vertical wires. Since a complete transmission between two wires (electron goes straight along the vertical or horizontal wire at the intersection without phase jump) can be viewed as a complete reflection between two local orbitals (electron completely jumps to the adjacent square at the intersection with a phase jump $-i$), the effective scattering angle for $\mathcal{B}$ should be $\pi/2$. One can directly check that the shifting $\theta \to \theta + \pi/2$ satisfies all these conditions.

After applying the above transformation to every corner of Chern blocks, the scattering potential $v\theta\delta_x$ between horizontal and vertical wires will be mapped to $v(\theta + \pi/2)\delta_x$ between the circular chiral modes at nearby squares (choosing $n = 0$). Therefore, the network model Hamiltonian can be equivalently written as

$$\begin{aligned}
H_{N,0} =& \sum_{nm}\sum_{\alpha}\int \mathrm{d}x\, \psi^\dagger_{n,m}(x,\alpha)(-i\alpha v\partial_x)\psi_{n,m}(x,\alpha)\\
H_{N,1} =& \sum_{nm}\sum_{\alpha} a\tilde{t}\,[\psi^\dagger_{n,m}(0,\alpha)\psi_{n,m}(0,\bar{\alpha}) + \psi^\dagger_{n,m}(a,\alpha)\psi_{n(m+\alpha)}(a,\bar{\alpha})\\
&+ \psi^\dagger_{n,m}(2a,\alpha)\psi_{(n-\alpha)(m+\alpha)}(2a,\bar{\alpha}) + \psi^\dagger_{n,m}(3a,\alpha)\psi_{(n-\alpha)m}(3a,\bar{\alpha})]
\end{aligned} \tag{55}$$

where $\bar{\alpha} = -\alpha$, and $a\tilde{t} = v(\theta + \pi/2)$ is the transformed scattering potential. For $\alpha = 1$ ($\alpha = -1$) the four terms in $H_{N,1}$ couples $\psi^\dagger_{n,m}$ to its neighboring circular chiral states in the right lower (left upper), right upper (left lower), left upper (right lower), left lower (right upper) directions, respectively. Again, the reader should not confuse the '$x$' in Eq. (55) with the global

coordinate $(x,y)$ defined in Sec. III B. '$x$' in Eq. (55) is the coordinate inside one square, *i.e.*, the $x_\alpha$ defined in Supplementary Fig. 21(a), and we have ignored the subscript $\alpha$ for simplicity .

When $\theta = -\frac{\pi}{2}$, we can see that $\tilde{t} = \frac{v}{a}(\theta + \pi/2) = 0$ and hence the circular chiral modes are decoupled from each other. Then the kinetic energy Hamiltonian $H_{N,0}$ can be diagonalized in representation of the circular chiral modes,

$$\phi^\dagger_{n,m}(K,\alpha) = \frac{1}{\sqrt{4a}} \int \mathrm{d}x_\alpha \exp\left[i\alpha \frac{2\pi K}{4a} x_\alpha\right] \psi^\dagger_{n,m}(x_\alpha,\alpha) \qquad \alpha = \pm 1,\ K \in \mathbb{Z}\,. \tag{56}$$

$\phi_{n,m}(K,\alpha)$ can be thought of as a standing wave going around the $C = \alpha$ Chern block in the unit cell $(n,m)$. $K$ being an integer is required by the periodicity $\psi_{n,m}(x_\alpha,\alpha) = \psi_{n,m}(x_\alpha + 4a,\alpha)$. Note that we use $\phi_{n,m}(K,\alpha)$ to denote the standing wave with phase factor $\propto \alpha K x_\alpha$. Such a special choice has the convenience that kinetic energy of $\phi_{n,m}(K,\alpha)$ depends on $K$ only, and this is due to the special direction choice of $x_\alpha$. For the red square, $x_+$ increases along the direction of chiral wires, so that a standing wave with phase factor $\propto Kx_+$ has kinetic energy $vK\pi/2a$. For the blue square, $x_-$ increases against the chiral direction, so that a standing wave with phase factor $\propto -Kx_-$ also has kinetic energy $vK\pi/2a$.

The inverse transformation is

$$\psi^\dagger_{n,m}(x_\alpha,\alpha) = \frac{1}{\sqrt{4a}} \sum_K \exp\left[-i\alpha \frac{2\pi K}{4a} x_\alpha\right] \phi^\dagger_{n,m}(K,\alpha)\,. \tag{57}$$

After the Fourier transform, $H_{N,0}$ can be written as

$$H_{N,0} = \sum_{n,m,\alpha,K} \frac{\pi v K}{2a} \phi^\dagger_{n,m}(K,\alpha)\phi_{n,m}(K,\alpha)\,. \tag{58}$$

And $H_{N,1}$ introduces hoppings between the standing waves:

$$\begin{aligned} H_{N,1} =& \frac{\tilde{t}}{4} \sum_{nm\alpha} \sum_{KK'} \Xi_{K'_{\max}}\left[\alpha K + \bar{\alpha}K'\right] \Big[ \phi^\dagger_{n,m}(K,\alpha)\phi_{n,m}(K',\bar{\alpha}) + e^{-i\alpha\frac{\pi}{2}K + i\bar{\alpha}\frac{\pi}{2}K'} \phi^\dagger_{n,m}(K,\alpha)\phi_{n,m+\alpha}(K',\bar{\alpha}) \\ &+ e^{-i\alpha\pi K + i\bar{\alpha}\pi K'} \phi^\dagger_{n,m}(K,\alpha)\phi_{n-\alpha,m+\alpha}(K',\bar{\alpha}) + e^{-i\alpha\frac{3\pi}{a}K + i\bar{\alpha}\frac{3\pi}{a}K'} \phi^\dagger_{n,m}(K,\alpha)\phi_{n-\alpha,m}(K',\bar{\alpha}) \Big]\,. \end{aligned} \tag{59}$$

Here we introduce a cutoff factor $\Xi_{K'_{\max}}[\alpha K + \bar{\alpha}K']$ (defined in Eq. (37)) to describe the broadening of the $\delta$ scattering potential. Note that $\frac{\pi}{2a}(\alpha K + \bar{\alpha}K')$ is the transferred momentum in the scattering. $K'_{\max}$ should have the order of $2a\Lambda_2/\pi$, with $\Lambda_2$ being the broadening truncation in the wire construction introduced in Supplementary Sec. III B. Therefore, we have successfully rewritten the network model as a lattice model with an infinite number of orbitals per cell (we have not introduced the cutoff $\Lambda_1$ on $K$), where $H_{N,0}$ sets the on-site energy and $H_{N,1}$ are the hopping terms.

Eqs. (58) and (59) should have the same symmetries as the original network model. The actions of $C_{2z}T$, $C_{4z}$, and $M_{xy}$ (defined in Supplementary Sec. III A) act on the circular chiral basis as

$$\begin{aligned} (C_{2z}T)\psi_{n,m}(x,\alpha)(C_{2z}T)^{-1} &= \psi_{-n-1,-m-1}(x,\bar{\alpha}) \\ C_{4z}\psi_{n,m}(x,\alpha)C_{4z}^{-1} &= \psi_{-m-1,n+1-(1+\alpha)/2}(x+a,\alpha)\,. \\ M_{xy}\psi_{n,m}(x,\alpha)M_{xy}^{-1} &= \psi_{m,n}(4a-x,\bar{\alpha}) \end{aligned} \tag{60}$$

Applying these actions to the standing wave basis, one should obtain

$$\begin{aligned} (C_{2z}T)\phi_{n,m}(K,\alpha)(C_{2z}T)^{-1} &= \phi_{-n-1,-m-1}(K,\bar{\alpha}) \\ C_{4z}\phi_{n,m}(K,\alpha)C_{4z}^{-1} &= e^{i\frac{\pi}{2}\alpha K}\phi_{-m-1,n+1-(1+\alpha)/2}(K,\alpha)\,. \\ M_{xy}\phi_{n,m}(K,\alpha)M_{xy}^{-1} &= \phi_{m,n}(K,\bar{\alpha}) \end{aligned} \tag{61}$$

It is direct to check that they commute with Eqs. (58) and (59).

In order to obtain the band structure of Eqs. (58) and (59), we introduce the Bloch basis of the standing wave basis as

$$\phi_{n,m}(K,\alpha) = \frac{1}{N} \sum_{\vec{q}} \exp\left(i\vec{q}\cdot(\vec{R}_{nm} + \vec{t}_\alpha)\right) \phi_{\vec{q}}(K,\alpha) \tag{62}$$

where $N$ is the number of unit cells in each direction, and $\vec{R}_{nm} = (2na, 2ma)$ and $\vec{t}_\alpha = ((2-\alpha)a/2, (2+\alpha)a/2)$ are the cell position and the relative position of square center under the *global* coordinate system (see the first paragraph in Supplementary

Sec. III B), respectively. $\vec{q}$ takes values in the first Brillouin zone $[-\pi/(2a), \pi/(2a)) \times [-\pi/(2a), \pi/(2a))$. (Note that the lattice constant is $2a$.) We then obtain the Hamiltonian in reciprocal space:

$$\begin{aligned} H_{N,0} &= \sum_{\vec{q}} \sum_{\alpha K} \frac{\pi v K}{2a} \phi^\dagger_{\vec{q}}(K,\alpha)\phi_{\vec{q}}(K,\alpha) \\ H_{N,1} &= \frac{\tilde{t}}{4} \sum_{\vec{q}} \sum_{\alpha} \sum_{K_1,K_2} \Xi_{K'_{\text{max}}}[\alpha K_1 + \bar{\alpha} K_2] \Big[ e^{i\alpha(q_x a - q_y a)} + (-\alpha i)^{(K_1+K_2)} e^{i\alpha(q_x a + q_y a)} \\ &\qquad + (-1)^{(K_1+K_2)} e^{-i\alpha(q_x a - q_y a)} + (\alpha i)^{(K_1+K_2)} e^{-i\alpha(q_x a + q_y a)} \Big] \phi^\dagger_{\vec{q}}(K_1,\alpha)\phi_{\vec{q}}(K_2,\bar{\alpha}) \end{aligned} \quad . \tag{63}$$

As in Eq. (59), for $\alpha = 1$ $(-1)$, the four terms in above $H_{N,1}$ correspond to couplings between neighbor circular chiral states in the right lower (left upper), right upper (left lower), left upper (right lower), left lower (right upper) directions, respectively.

In actual calculations, we should take a cutoff on $K$ ($K \in [K_{\text{min}}, K_{\text{max}}]$), and there will be $2(K_{\text{max}} - K_{\text{min}} + 1)$ bands in total. Note that the network model and un-truncated lattice model represent the same system on different bases. Eq. (63) will follow the band periodicity of the network model when $(K_{\text{max}} - K_{\text{min}}) \to \infty$. Hence, only $K_{\text{max}} - K_{\text{min}}$ matters, and we can take $K_{\text{min}} = 0$ for notation convenience. We notice that $K_{\text{max}}$ plays the same role as $\Lambda_1$ in the network model. So in principle, the cutoff $K'_{\text{max}}$ of $\delta$-potential in $H_{N,1}$, should be (i) much smaller than $K_{\text{max}}$ such that the degrees of freedom in chiral edges can be treated as continuous and (ii) much larger than one such that the characteristic length of the $\delta$-potential is much smaller than the unit cell size. (See the discussions in Supplementary Sec. III C for details). Numerical calculation confirms that the middle eight bands of Eq. (63) can approximately reproduce the band structure of the network model (Supplementary Fig. 18) in one repeating unit when $K_{\text{max}} = 7$ and $K'_{\text{max}} = 2$. To reproduce more repeating units, a higher cutoff is needed, *e.g.*, $K_{\text{max}} = 12$, $K'_{\text{max}} = 2$ for two repeating units.

### B. Eight-band lattice model $H_{8B}$

The above analysis is a basis transformation of Eq. (36). Eq. (63) will reproduce the band structure of the network model when $K_{\text{max}}$, $K'_{\text{max}} \to \infty$ and $K'_{\text{max}}/K_{\text{max}} \to 0$. This verifies that the un-truncated lattice model is an equivalent description of the network model with local basis. However, the un-truncated lattice model still contains infinite bands and numerically requires a high cutoff to reproduce the band structure of the network model. In the end of Supplementary Sec. IV A, we mentioned that 15 bands are needed to reproduce the network model's band structure in one repeating window. In this subsection, we attempt to construct a truncated lattice model that contains fewer bands but still capture the low energy physics of the network model.

In this paragraph, we sketch the procedure that leaded us to the desired model and show its properties in later paragraphs. We started from a large $K_{\text{max}}$ such as 200 and fixed $K'_{\text{max}}$ to some finite integer such as 10. Then we decreased $K_{\text{max}}$ until the low energy physics is essentially changed. "Low energy physics" here means the physics near the Fermi surface which is chosen as the middle of the bands to minimize the effects of cutoff on $K$. When $K_{\text{max}} \gg K'_{\text{max}}$, we had basically the same band structure as the network model except for several highest and lowest bands that are significantly influenced by the cutoff on $K$. As $K_{\text{max}}$ decreased, the band structure gradually changed away from that of the network model. Such a deviation is not uniform for all the bands, the middle bands deviate less while the high-lying and low-lying bands deviate more. When $K_{\text{max}} \gtrsim K'_{\text{max}}$, the 8-band periodicity of the network model cannot be reproduced, but some low energy properties of the network model (will shown later) still remain. Finally, we got the minimal model with $K_{\text{max}} = 3$ (eight bands) that can reproduce the low energy physics of the network model and we will justify this result later. We refer to the resulting Hamiltonian as $H_{8B}$

One may notice that the condition $\Lambda_1 \gg \Lambda_2$ in the network model is now violated. Cutoff $K_{\text{max}}$ discretizes the chiral edges and $K'_{\text{max}}$ broaden the $\delta$ scattering potential. In the network model and its mapping to the un-truncated lattice model, we require the granularity of chiral edges to be much smaller than the broaden of the $\delta$ potential. But this requirement is violated now, since we take $K_{\text{max}} < K'_{\text{max}}$ (and $K'_{\text{max}} = \infty$ effectively) on the way to $H_{8B}$, although it finally turns out that $H_{8B}$ can reproduce the network model's low energy physics. Nevertheless, we can offer two reasons about why $H_{8B}$ with $K_{\text{max}} = 3$, $K'_{\text{max}} = \infty$ is a valid approximation. First, the repeating unit of the the network model's band structure comprises eight bands, so it is reasonable to presume that the essential physics of the network model can be realized by only eight bands, *i.e.*, $K_{\text{max}} = 3$. Second, when $K_{\text{max}} = 3$, the condition $K_{\text{max}} \propto \Lambda_1 \to \infty$ is not satisfied, *i.e.*, the granularity of chiral edges is too coarse to discriminate Supplementary Fig. 19(a) and Supplementary Fig. 19(b). And numerical calculation confirmed that, given $K_{\text{max}} = 3$, different $K'_{\text{max}}$ choices result in similar band structures. Therefore, we can take $K'_{\text{max}} = \infty$, *i.e.*, ignore the truncation factor $\Xi_{K'_{\text{max}}}$ for simplicity. Now, we will show the numerical results and illustrate that $H_{8B}$ indeed captures the low energy features of the network model's band structure.

We calculate the band structure and representations at high-symmetry points of $H_{8B}$ and show the results in Supplementary Fig. 22. (We also shift the zero energy to the middle of the eight bands.) First, if we take the Fermi energy in the middle of the eight bands (the gray dashed lines in Supplementary Fig. 22), *i.e.*, $E_{F-8B} = 0$, the system is insulating when $\tilde{t} \to 0$

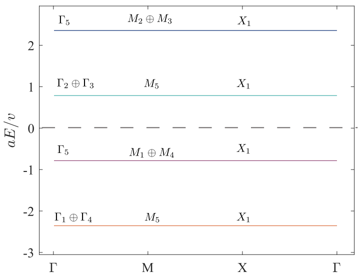


(a)$\tilde{t} = 0$

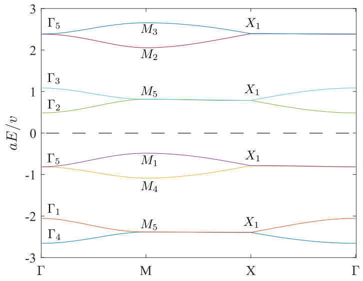


(b)$\tilde{t} = 0.3v/a$

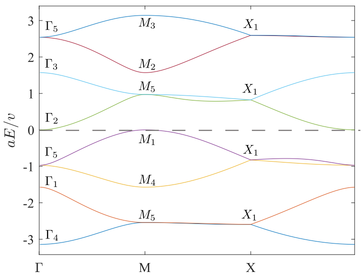


(c)$\tilde{t} = \pi v/4a$

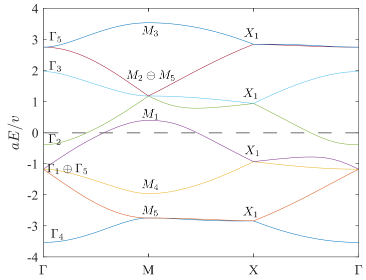


(d)$\tilde{t} = 3\pi v/8a$

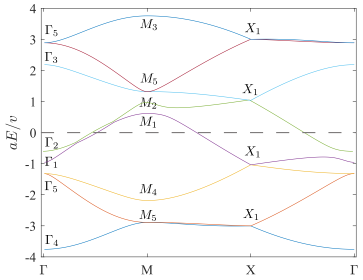


(e)$\tilde{t} = 1.4v/a$

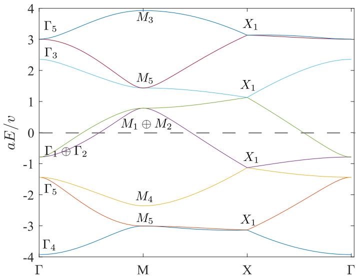


(f)$\tilde{t} = \pi v/2a$

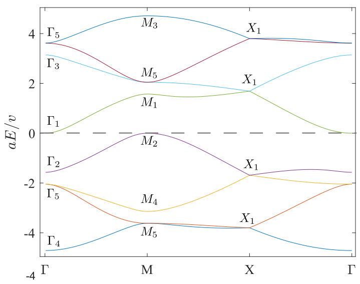


(g)$\tilde{t} = 3\pi v/4a$

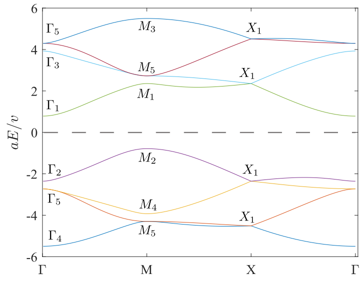


(h)$\tilde{t} = \pi v/a$

Supplementary Fig. 22. Band structures of lattice model $H_{8B}$ with different scattering strengths $\tilde{t}$. The gray dashed lines indicate the Fermi level $E_F = 0$. Note that we have shifted the zero energy to the middle of the eight bands.

and $\tilde{t} \to \pi v/a$. (Even though bands are not flat for $\tilde{t} = \pi v/a$ as they were in the network model.) This is indeed the case for the network model with a Fermi level $E_{F-N} = \pi v/4a$ laid in the middle of a repeating unit (the gray dashed lines in Supplementary Fig. 18). Second, for $\tilde{t} \approx \pi v/2a$, the band structure of the two bands closest to the Fermi level $E_{F-8B}$ is similar to a superposition of two 1D bands with linear dispersion in $x$ & $y$ direction, respectively (see Supplementary Fig. 23). Such a superposition is approximately the quasi-1D band structure of the network model with $\theta = 0$ ($\tilde{t} = \pi v/2a$), *i.e.*, which consists of decoupled chiral wires put along $x$ & $y$ directions, respectively (see the inset of Fig. 1(h)).

Another supporting evidence is the change of representations near the Fermi level in the phase transition. As $\tilde{t}$ increases from $0$ to $\pi v/a$, $\Gamma_1$, $M_1$ rise across the Fermi level while $\Gamma_2$, $M_2$ fall below the Fermi level. In the network model, if we take the red dashed pane (contains eight bands) in Supplementary Fig. 18 as a repeating unit and set the Fermi level at the gray dashed line in Supplementary Fig. 18 (the middle of the unit), during the transition ($\theta = -\pi/2 \to \pi/2$), the same pairs of representations will switch across the Fermi level in the same way. One may ask why we choose the upper eight bands in Supplementary Fig. 18 rather than the lower eight as the ‘repeating unit. The reason is that the standing waves with $K \in [0, 3]$ at $\tilde{t} = 0$ (Supplementary Fig. 22(a)) correspond to the upper eight bands in Supplementary Fig. 18(a) at $\theta = -\pi/2$ (Supplementary Fig. 18(a)). Based on these observations and the numerical results that will be presented in Sec. I, we claim that $H_{8B}$ correctly captures the low energy physics of the network model in Sec. III.

Despite similarities in low energy physics, we must point out that the BR transition of $H_{8B}$ differs from that of the network model. In the network model with a sufficient number of bands, the four bands below the Fermi level and inside a repeating

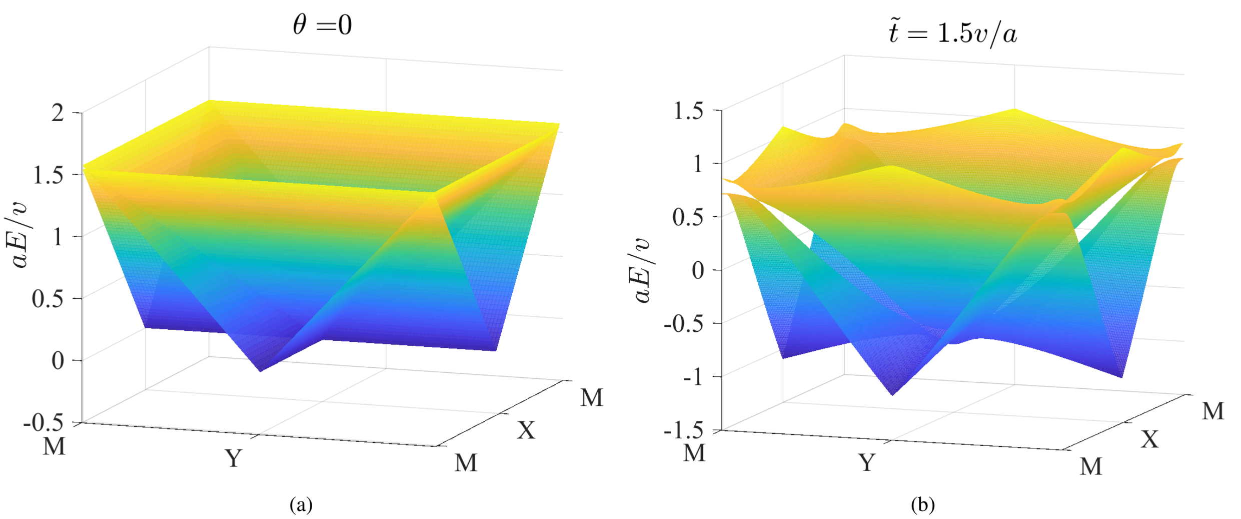


Supplementary Fig. 23. (a) The quasi-1D band structure of the network model in the metal limit ($\theta = 0$) and near the Fermi level ($E_{F-N} = \pi v/4a$). It is exactly a superposition of two 1D bands with linear dispersion in $x$ & $y$ directions (chiral wires in $x$ & $y$ directions), respectively. The corresponding plot in the main text (the inset of Fig. 1(h)) chooses $E_{F-N}$ here as the zero energy. (b) The quasi-1D band structure of $H_{8B}$ in the metal limit ($\tilde{t} \approx \pi v/2a$) and near the Fermi level ($E_{F-8B} = 0$). It is similar to (a) but with a minor coupling between two 1D subsystems. Note that we have shifted the zero energy of $H_{8B}$ to the middle of the eight bands. And the corresponding plot in the main text (inset of Fig. 2(d)) chooses the same zero energy.

unit (inside the red pane and below the gray dashed line in Supplementary Fig. 18) not only exchange representations with the bands above them but also with bands (in another repeating unit) below them. During the transition ($\theta = -\pi/2 \to \pi/2$), these four bands change from ${}^2\mathrm{E_b}\uparrow\mathrm{G} \oplus \mathrm{A_b}\uparrow\mathrm{G}$ to ${}^1\mathrm{E_b}\uparrow\mathrm{G} \oplus \mathrm{B_b}\uparrow\mathrm{G}$. As we explained, such four bands corresponds to the lower four bands of $H_{8B}$. However, in $H_{8B}$, there is no band below these four bands. As a result, different from the network model, the lower four bands of $H_{8B}$ change from ${}^2\mathrm{E_b}\uparrow\mathrm{G} \oplus \mathrm{A_b}\uparrow\mathrm{G}$ to $\mathrm{A_c''}\uparrow\mathrm{G}$ during the transition ($\tilde{t} = 0 \to \pi v/a$). The Wyckoff position of $\mathrm{A_c''}\uparrow\mathrm{G}$ is 4c, which is the $C_{2z}T$ center rather than the $C_{4z}$ center (Supplementary Table II). This difference of molecular orbital transition is unavoidable since the transition in the network model involves exchanges of representations between different repeating units, while $H_{8B}$ only has one unit. Nevertheless, since the origin of this difference is well below the Fermi level, it is not a difference in the low energy physics. And we have seen that $H_{8B}$ and the network model have the same representation exchange near the Fermi surface; therefore, $H_{8B}$ still reproduces the low energy physics of the network model.

It is worth mentioning that the transition in $H_{8B}$ changes the position of the MEBRs from $C_{4z}$-centers (2b) to $C_{2z}T$-centers (4c) (see Supplementary Fig. 24). No $C_{2z}T$ center is occupied before the transition, and the Real Space Invariant $\delta_w = 0$. Given that there are four $C_{2z}T$ centers per cell and four occupied bands, every $C_{2z}T$ center is occupied by one electron after the transition, and the system has Real Space Invariant $\delta_w = 1$. Therefore, the second Stiefel-Whitney class $w_2$ [10, 11] must change from 0 to 1. The transition process must involve braiding of the Dirac points. We will discuss this in detail in Supplementary Sec. IV D. In addition, although the lower four bands form $\mathrm{A_c''}\uparrow\mathrm{G}$ are always connected in our models, in general cases, $\mathrm{A_c''}\uparrow\mathrm{G}$ can be decomposed into two fragile topological bands ($\Gamma_5,\ M_5,\ X_1$) and two trivial bands (forming MEBR $A_2\uparrow G$ with Wyckoff position $2a$ $(\frac{1}{4}\,\frac{1}{4},0)$, $(\frac{3}{4}\,\frac{3}{4},0)$, *i.e.*, the centers of white squares in Supplementary Fig. 16(a)), which is expected from $w_2 = 1$.

### C. $H_{8B}$ on corner state basis

In previous sections, we have seen that the low energy physics of the network model can be reconstructed using four standing waves per square. In order to obtain a more local tight-binding model, we can convert the standing waves into four "corner states" $c_{n,m}(w,\alpha)$ $(w = 1,2,3,4)$, which are wave-packets centered at four corners of a square (see Supplementary Fig. 24). The relation between the "corner states" and the standing waves is the Fourier transform on each square:

$$\begin{pmatrix} c^\dagger_{n,m}(1,\alpha) \\ c^\dagger_{n,m}(2,\alpha) \\ c^\dagger_{n,m}(3,\alpha) \\ c^\dagger_{n,m}(4,\alpha) \end{pmatrix} = \frac{1}{2}\begin{pmatrix} 1 & 1 & 1 & 1 \\ 1 & -\alpha i & -1 & \alpha i \\ 1 & -1 & 1 & -1 \\ 1 & \alpha i & -1 & -\alpha i \end{pmatrix}\begin{pmatrix} \phi^\dagger_{n,m}(0,\alpha) \\ \phi^\dagger_{n,m}(1,\alpha) \\ \phi^\dagger_{n,m}(2,\alpha) \\ \phi^\dagger_{n,m}(3,\alpha) \end{pmatrix} = U_\alpha \begin{pmatrix} \phi^\dagger_{n,m}(0,\alpha) \\ \phi^\dagger_{n,m}(1,\alpha) \\ \phi^\dagger_{n,m}(2,\alpha) \\ \phi^\dagger_{n,m}(3,\alpha) \end{pmatrix}, \tag{64}$$

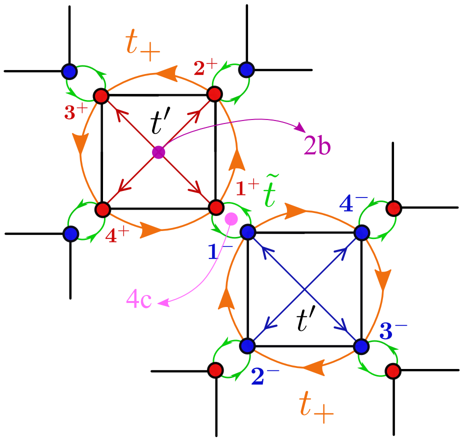


Supplementary Fig. 24. Unit cell of $H_{8B}$ and the corner state basis. The orange and green curves with arrows illustrate the hoppings of Eq. (66). Inside one square, $t_+ = -(1+i)\pi v/4a$ denote the square edge hopping and $t' = -\pi v/4a$ denotes the square diagonal hopping. The direction of $t_+$ is indicated by the orange arrows surrounding the square. $\tilde{t}$ denotes the nearest neighbor (NN) hopping between squares. The red & blue circles and decorated numbers $w^\pm$ ($w = 1, 2, 3, 4$) indicate the positions of corner states $c_{n,m}(w, \pm)$. 4c and 2b indicate the (representative of) corresponding Wyckoff positions.

where the transform coefficient between $c^\dagger_{n,m}(w,\alpha)$ and $\phi^\dagger_{n,m}(K,\alpha)$ is $(U_\alpha)_{w,K+1} = e^{-i\frac{\pi}{2}\alpha K(w-1)}$. More explicitly, in the real space representation

$$\langle 0|\psi_{n,m}(x_\alpha)c^\dagger_{n,m}(w,\alpha)|0\rangle = \frac{1}{2}\sum_{K=0}^{3} e^{i\frac{\pi}{2}\alpha K[x_\alpha-(w-1)a]}. \tag{65}$$

The right side does not contain $n, m$ because $x_\alpha$ particularly refers to the coordinate around the $\alpha$ square in the $(n,m)$ cell as defined in Supplementary Fig. 21(a). From Eq. (65), we can see that $c_{n,m}(w,\alpha)$ represents a superposition of standing wave states with different wave vectors $K = [0,3]$ and the same phase origin $x_{\alpha,w} = (w-1)a$ in the $\alpha$ square of $(n,m)$ cell. (Further explanation of $x_{\alpha,w}$: it represents a specific point in the $\alpha$ square with coordinate value $(w-1)a$ in terms of coordinate $x_\alpha$.) Hence, $c_{n,m}(\alpha,w)$ should be a wave packet centered at $x_{\alpha,w} = (w-1)a$, *i.e.*, at a corner of the $\alpha$ square of $(n,m)$ cell. The four $c_{n,m}(\alpha,w)$ defined in Eq. (64) respectively center at four $\alpha$ square corners according to Supplementary Fig. 24, and we call them "corner states". Since each corner state is a summation over only four waves, it has a considerable broadening that results in various hoppings shown in Eq. (66).

Substituting Eq. (64) into Eqs. (58) and (59) we obtain a tight-binding Hamiltonian on the corner state basis. Note that the notation in Eq. (66) is different from that of Eq. (4). The site indices $p, q$ in Eq. (4) become $(n,m,\alpha,w)$ here. Square edge and diagonal hopping, *i.e.*, $\langle\langle\cdot\rangle\rangle$ and $\langle\langle\langle\cdot\rangle\rangle\rangle$, are described by $B_\alpha$ in the following $H_{8\mathrm{B},0}$. And the nearest neighbor hopping $\langle\cdot\rangle$ corresponds to the following $H_{8\mathrm{B},1}$.

$$\begin{aligned} H_{8\mathrm{B},0} &= \sum_{nm}\sum_{\alpha}\sum_{w_1 w_2} (B_\alpha)_{w_1,w_2}\, c^\dagger_{n,m}(w_1,\alpha)c_{n,m}(w_2,\alpha) \\ H_{8\mathrm{B},1} &= \sum_{nm}\sum_{\alpha} \tilde{t}\,[c^\dagger_{n,m}(1,\alpha)c_{n,m}(1,\bar\alpha) + c^\dagger_{n,m}(2,\alpha)c_{n,(m+\alpha)}(2,\bar\alpha) + \\ &\qquad c^\dagger_{n,m}(3,\alpha)c_{(n-\alpha),(m+\alpha)}(3,\bar\alpha) + c^\dagger_{n,m}(4,\alpha)c_{(n-\alpha),m}(4,\bar\alpha)] \end{aligned} \tag{66}$$

where

$$B_\alpha = U^\dagger_\alpha \mathbb{K} U_\alpha = \frac{\pi v}{4a}\begin{pmatrix} 0 & \alpha i-1 & -1 & -\alpha i-1 \\ -\alpha i-1 & 0 & \alpha i-1 & -1 \\ -1 & -\alpha i-1 & 0 & \alpha i-1 \\ \alpha i-1 & -1 & -\alpha i-1 & 0 \end{pmatrix} \qquad \mathbb{K} = \begin{pmatrix} 0 & 0 & 0 & 0 \\ 0 & \frac{\pi v}{2a} & 0 & 0 \\ 0 & 0 & \frac{\pi v}{a} & 0 \\ 0 & 0 & 0 & \frac{3\pi v}{2a} \end{pmatrix} - \frac{3\pi v}{4a}\mathbb{I}_4\,. \tag{67}$$

Here $\mathbb{K}$ represents the on-site energies of the standing wave basis ($K = 0,1,2,3$) in Eq. (58). We have shifted these on-site energies by a constant $-\frac{3\pi v}{4a}$ such that the energy bands are centered at the zero energy. Eq. (66) can be visualized by Supplementary Fig. 24. Inside one square, there are both the square diagonal and square edge hoppings: $t' = -\frac{\pi v}{4a}$ and

$t_+ = -(1+i)\frac{\pi v}{4a}$ (along the directions indicated by the orange arrows around the square). For different squares, there are real hoppings $\tilde{t}$ between adjacent corners (green arrows in Supplementary Fig. 24).

In the corner state basis, the nature of the phase transition becomes more conspicuous. When $\tilde{t}$ approaches 0, electrons mainly go around the squares, *i.e.*, around $C_{4z}$ centers. As $\tilde{t} \to +\infty$, electrons are trapped in corners *i.e.*, $C_{2z}T$ centers.

The actions of $C_{2z}T$, $C_{4z}$, $M_{xy}$ on the corner state basis are

$$\begin{aligned} (C_{2z}T)c_{n,m}(w,\alpha)(C_{2z}T)^{-1} &= c_{-n-1,-m-1}(w,\bar{\alpha}) \\ C_{4z}c_{n,m}(w,\alpha)C_{4z}^{-1} &= c_{-m-1,n+1-(1+\alpha)/2}(w+1 \bmod 4,\alpha)\ . \\ M_{xy}c_{n,m}(w,\alpha)M_{xy}^{-1} &= c_{m,n}(4-w \bmod 4,\bar{\alpha}) \end{aligned} \tag{68}$$

Basically, they are the same as the actions on the circular chiral basis (Eq. (60)), but now the continuous coordinate $x_\alpha$ is discretized to $w$. One can check that the Hamiltonian in Eq. (66) respects the crystalline symmetries.

### D. Evolution of the Dirac points

As shown in Supplementary Fig. 22, as we tune $\tilde{t}$ from 0 to $\frac{\pi v}{a}$, a pair of Dirac points is created on the $\Gamma M$ pave. Due to the $C_{4z}$ symmetry, there should be four pairs of Dirac points created along equivalent paths. As $\lambda$ continues to increase, four Dirac points move to $\Gamma$ and then annihilate each other, and the other four move to $M$ and then annihilate each other (shown in Supplementary Fig. 25(a)).

In a generic multi-band system with $C_{2z}T$ symmetry, the only topological charge of a Dirac point is its $\pi$ Berry's phase, which is $Z_2$-valued. However, if the considered two bands are disconnected from other bands, the Dirac points instead carry $Z$-valued topological charges, *i.e.*, chiralities, characterized by the non-Abelian Berry's connection [10, 12, 13]. Even if the considered two bands are connected to other bands, locally two Dirac points can only annihilate each other if they have opposite chiralities. Thus, given that we are only interested in a small region of the Brillouin zone where the two bands are well separated from other bands, we can still talk about this $Z$-valued topological charge [10, 12, 13]. That means if in the evolution (i) the Dirac points do not move outside this region and (ii) the two bands in this region are always well separated from other bands, then two Dirac points will annihilate each other only if they carry opposite chiralities.

We first look at the four Dirac points annihilated at $\Gamma$. When they are close to $\Gamma$, they can be described by a k·p model around $\Gamma$. We will derive the chiralities of the Dirac points by studying such a k·p model. The two involved energy levels at $\Gamma$ are $\Gamma_1$ and $\Gamma_2$. According to the character table in Supplementary Table I, we can write the symmetry operators as

$$C_{4z} = \sigma_z, \qquad M_{xy} = \sigma_z, \qquad C_{2z}T = K, \tag{69}$$

where $K$ is the complex conjugation. $C_{2z}T$ restricts the Hamiltonian to be a real matrix. Thus, we parameterize the Hamiltonian as

$$H(\vec{k}) = \epsilon_0(\vec{k})\sigma_0 + Z(\vec{k})\sigma_z + X(\vec{k})\sigma_x \tag{70}$$

with $\epsilon_0(\vec{k}), Z(\vec{k}), X(\vec{k})$ being real valued parameters to be determined. The $C_{4z}$ symmetry implies

$$\epsilon_0(-k_y,k_x) = \epsilon_0(k_x,k_y), \qquad Z(-k_y,k_x) = Z(k_x,k_y), \qquad X(-k_y,k_x) = -X(k_x,k_y)\ . \tag{71}$$

The mirror symmetry $M_{xy}$ implies

$$\epsilon_0(k_y,k_x) = \epsilon_0(k_x,k_y), \qquad Z(k_y,k_x) = Z(k_x,k_y), \qquad X(k_y,k_x) = -X(k_x,k_y)\ . \tag{72}$$

To second order of $\vec{k}$, $\epsilon_0, Z, X$ must hence have the forms

$$\epsilon_0(\vec{k}) = E_0 + C\vec{k}^2, \qquad Z(\vec{k}) = -\Delta + A\vec{k}^2, \qquad X(\vec{k}) = B(k_x^2 - k_y^2)\ , \tag{73}$$

respectively. Here $A, B, C, E_0, \Delta$ are all real parameters. $Z(\vec{k})$ is the term that creates band inversion at the $\Gamma$ point The band energies are given by

$$E_\pm(\vec{k}) = E_0 + C\vec{k}^2 \pm \sqrt{(\Delta - A\vec{k}^2)^2 + B^2(k_x^2-k_y^2)^2}\ . \tag{74}$$

If $A\cdot\Delta > 0$, then there are four Dirac points locating at $k_y = \pm k_x$, $k_x = \pm\sqrt{\Delta/A}$. When $\Delta \to 0$, the four Dirac points move to $\Gamma$ to annihilate each other. Some quantitative constraints on $A, B, C, \Delta$ can be inferred from Supplementary Fig. 22. First, the

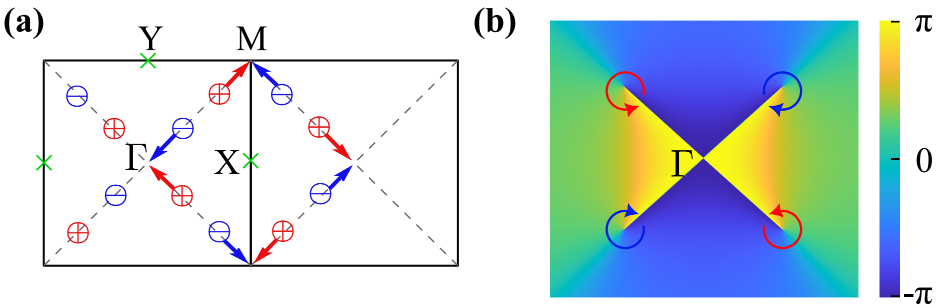


Supplementary Fig. 25. Evolution of Dirac points. (a) The trajectory of the Dirac points, where red and blue circles represent Dirac points with positive and negative chiralities, respectively. (b) An illustration of the phase $\varphi_{\vec{k}}$ in the k·p expansion around Γ point. Anti-clockwise (clockwise) winding of $\varphi_{\vec{k}}$ around a Dirac point indicates a positive (negative) chirality.

Dirac points exist when $\Gamma_2$ has a higher energy than $\Gamma_1$, *i.e.*, $\Delta > 0$. Then there must be $A > 0$ due to the existence condition $A \cdot \Delta > 0$ of Dirac points. Second, since the sign of the off-diagonal coefficient $B$ can be changed by a gauge transformation $e^{i\frac{\pi}{2}\sigma_z}$, the sign of $B$ is a gauge choice and we choose $B > 0$. Third, when $\Delta = 0$ (Supplementary Fig. 22(e)), both the two bands increase in energy with increasing $\vec{k}$ in the $\Gamma M$ direction, implying $C > A > 0$. Fourth, when $\Delta = 0$, one band increases in energy while the other decreases in energy along the $\Gamma X$ direction, implying $C < \sqrt{A^2 + B^2}$.

The eigenstates corresponding to $E_\pm(\vec{k})$ are

$$|u_+\rangle = e^{i\varphi(\vec{k})/2} \begin{pmatrix} \cos\frac{1}{2}\varphi(\vec{k}) \\ \sin\frac{1}{2}\varphi(\vec{k}) \end{pmatrix}, \qquad |u_-\rangle = e^{i\varphi(\vec{k})/2} \begin{pmatrix} \sin\frac{1}{2}\varphi(\vec{k}) \\ -\cos\frac{1}{2}\varphi(\vec{k}) \end{pmatrix} \tag{75}$$

with $\varphi_{\vec{k}}$ given by

$$\varphi_{\vec{k}} = \arccos \frac{-\Delta + A\vec{k}^2}{\sqrt{(\Delta - A\vec{k}^2)^2 + B^2(k_x^2 - k_y^2)^2}} \,. \tag{76}$$

We illustrate $\varphi_{\vec{k}}$ in Supplementary Fig. 25(b). One can see that at the Dirac points, $\varphi_{\vec{k}}$ is not well-defined, and there is a nontrivial winding of $\varphi_{\vec{k}}$ around a Dirac point. The prefactors $e^{i\varphi(\vec{k})/2}$ in $|u_\pm(\vec{k})\rangle$ are needed for $|u_\pm(\vec{k})\rangle$ (away from Dirac points) to be single-valued because the entries of the two-by-one wavefunction vectors transform as $\sin\frac{\varphi}{2} \to -\sin\frac{\varphi}{2}$, $\cos\frac{\varphi}{2} \to -\cos\frac{\varphi}{2}$ upon $\varphi \to \varphi + 2\pi$. The sewing matrix of $C_{2z}T$ is given by

$$C_{2z}T|u_n(\vec{k})\rangle = |u_n(\vec{k})\rangle e^{i\theta_n(\vec{k})}, \qquad \theta_n(\vec{k}) = -\varphi(\vec{k}), \qquad (n = \pm) \,. \tag{77}$$

The non-Abelian Berry's connection can be calculated as

$$\vec{\mathcal{A}}_{m,n}(\vec{k}) = i\langle u_m(\vec{k})|\partial_{\vec{k}}|u_n(\vec{k})\rangle = \begin{pmatrix} -\frac{1}{2}\partial_{\vec{k}}\varphi(\vec{k}) & i\frac{1}{2}\partial_{\vec{k}}\varphi(\vec{k}) \\ -i\frac{1}{2}\partial_{\vec{k}}\varphi(\vec{k}) & -\frac{1}{2}\partial_{\vec{k}}\varphi(\vec{k}) \end{pmatrix}_{m,n} \,. \tag{78}$$

We compare it to the generic form of non-Abelian Berry's connection with $C_{2z}T$ symmetry [10]

$$\vec{\mathcal{A}}(\vec{k}) = \begin{pmatrix} \frac{1}{2}\partial_{\vec{k}}\theta_+(\vec{k}) & i\vec{a}(\vec{k})e^{i\frac{\theta_+(\vec{k})-\theta_-(\vec{k})}{2}} \\ -i\vec{a}(\vec{k})e^{-i\frac{\theta_+(\vec{k})-\theta_-(\vec{k})}{2}} & \frac{1}{2}\partial_{\vec{k}}\theta_-(\vec{k}) \end{pmatrix} , \tag{79}$$

where $\vec{a}$ is the real-valued off-diagonal Berry's connection and $\theta_\pm(\vec{k})$ are the phase factors appearing in the $C_{2z}T$ sewing matrix. $\vec{a}$ is gauge invariant up to a global ambiguity of $\pm$ sign. After the global sign is fixed (as will be done in Eq. (81)), the chirality of the $i$th Dirac point can be defined through $\vec{a}(\vec{k})$ as

$$\chi_i = \frac{1}{\pi}\oint_{\partial D_i} d\vec{k}\cdot\vec{a}(\vec{k}) \,, \tag{80}$$

where $D_i$ is an infinitely small disk containing the $i$th Dirac point and $\partial D_i$ is its boundary. Comparing Eq. (78) to Eq. (79), we choose $\theta_+(\vec{k}) = \theta_-(\vec{k}) = \varphi(\vec{k})$ and

$$\vec{a}(\vec{k}) = \frac{1}{2}\partial_{\vec{k}}\varphi_{\vec{k}} \,. \tag{81}$$

For this gauge choice, the chirality of a Dirac point is simply the winding number of $\varphi_{\vec{k}}$

$$\chi_i = \frac{1}{2\pi} \oint_{\partial D_i} d\vec{k} \cdot \partial_{\vec{k}} \varphi_{\vec{k}} \ . \tag{82}$$

(Another gauge choice can be obtained by replacing $|u_-(\vec{k})\rangle \to -|u_-(\vec{k})\rangle$, then there would be $\vec{a}(\vec{k}) = -\frac{1}{2}\varphi(\vec{k})$ and $\chi_i$ would be reversed.) As shown in Supplementary Fig. 25, the Dirac points at $(k_0, k_0)$, $(-k_0, k_0)$, $(-k_0, -k_0)$, $(k_0, -k_0)$ ($k_0 = \sqrt{\Delta/A}$) have the chiralities $-1$, $+1$, $-1$, $+1$, respectively. If the considered two bands were disconnected from other bands, then their Euler class was given by $\frac{1}{2}\sum_i \chi_i$ [10]. Even if the two bands are connected to other bands, $\chi_i$ is still a *locally* well-defined $Z$-valued quantity in the sense that two Dirac points can *locally* annihilate each other only if they carry opposite $\chi$'s.

One can see that two Dirac points related by the $C_{4z}$ operation have opposite chiralities. This is true even in the absence of the mirror symmetry $M_{xy}$, which allows $X(\vec{k})$ to have the form $B(k_x^2 - k_y^2) + B' 2k_x k_y$ to the second order of $\vec{k}$. By a proper re-definition of the coordinate ($\vec{k}'$), this term can be rewritten as $B''(k_x'^2 - k_y'^2)$ for some $B''$. Then the analysis in the above paragraph applies. Therefore, we conclude that as long as the two bases of the k·p expansion have $C_{4z}$ eigenvalues 1 and $-1$, a pair of $C_{4z}$-related Dirac points should have opposite chiralities.

The k·p theory at the $M$ point is equivalent to the one at $\Gamma$. The two involved levels at $M$ are $M_1$ and $M_2$. According to Supplementary Table I, we can write the symmetry operators as

$$C_{4z} = i\sigma_z, \qquad M_{xy} = -\sigma_z, \qquad C_{2z}T = K \ . \tag{83}$$

It is worth mentioning that, at the $M$ point, $C_{2z}T$ anti-commutes with $C_{4z}$ due to the translation part of $C_{2z}T$. Nevertheless, $C_{4z}$, $M_{xy}$, and $C_{2z}T$ impose the same constraints on the k·p Hamiltonian as those at $\Gamma$. Therefore, the analyses in the two paragraphs above also apply to $M$, and the $C_{4z}$-related Dirac points must have opposite chiralities. Suppose the four Dirac points locate at $(\pi - p_0, \pi - p_0)$, $(\pi + p_0, \pi - p_0)$, $(\pi + p_0, \pi + p_0)$, $(\pi - p_0, \pi + p_0)$, respectively, for some small positive $p_0$. The first Dirac point ($(\pi - p_0, \pi - p_0)$) must have $\chi = 1$ because it is emerged together with the Dirac point at $(k_0, k_0)$, which has $\chi = -1$ according to the last two paragraphs. Since $C_{4z}$-related Dirac points have opposite chiralities, the four Dirac points should have the chiralities $1$, $-1$, $1$, $-1$, respectively.

One can see that, upon the phase transition form the initial gapped state to the final gapped state, the trajectories of Dirac points form a closed path $\mathcal{C}$ separating $X$ from $Y$ (Supplementary Fig. 25). As shown in Supplementary Fig. 22, $X$ has a Dirac point between the third and fourth bands; $Y$ must also have a Dirac point according to $C_{4z}$. Drawing a path connecting $X$ and $Y$, it must cross $\mathcal{C}$ odd times. According to Refs. [10, 12, 13], the relative chirality between the Dirac points at $X$ and $Y$ changes after such a phase transition, as expected because the transition changes $w_2$.

### E. Simplified eight-band lattice model $H'_{8B}$

The motivation of a further simplification on $H_{8B}$ is to obtain a more convincing critical metal phase. We leave the detailed discussion of localization for Supplementary Sec. V & I and the disorder potential for Supplementary Sec. IV F. Here we merely quote the result: both the network model and $H_{8B}$ have a critical metal phase. However, in the clean limit, $H_{8B}$ has a finite density of states at the Fermi level ($DOS_F$) $E_F = 0$ in the critical region ($\tilde{t} \approx \pi v/2a$). Finite $DOS_F$ in the clean limit may lead to a large localization length that exceeds the numerical accessible transversal size. As explained in Supplementary Sec. V A, such a large localization length will weaken the validity of our proof of the critical metal phase in $H_{8B}$. Although we offer some evidences (see Supplementary Sec. V A) that the critical phase in $H_{8B}$ is reliable, a critical phase with $DOS_F = 0$ in the clean limit will be more convincing.

We first simplify $H_{8B}$ by removing the square diagonal hopping. The motivation is that the square diagonal hopping is not relevant for the phase transition: (i) without $t'$, $\tilde{t} = 0$ and $\tilde{t} = \infty$ still represent the molecular orbital limit with charge centers at $C_{4z}$-centers and the bonding state limit with charge centers at $C_{2z}T$-centers, respectively, (ii) omitting $t'$ does not change the symmetry class because time-reversal-symmetry (complex conjugation) is already broken by the complex square edge hopping $t_+$. Therefore, in the absence of $t'$, changing $\tilde{t} = 0$ to $\tilde{t} = \infty$ still realizes a phase transition of $w_2$ in the symmetry class A, where time-reversal symmetry is broken.

Removing square diagonal hopping changes the band structure near the Fermi level but $DOS_F$ is still finite (see Supplementary Fig. 26). Thus, in addition to omitting square diagonal hopping, we will replace the square edge hopping $t_+ = (1 + i)$ by $t = (1 + Ai)$ for some real parameter $A$. (One should not confuse it with the vector potential $\mathcal{A}$.) We denote the eight-band lattice model with these modifications as $H'_{8B}$. We leave the numerical localization calculation of $H'_{8B}$ for Supplementary Sec. I. Here we merely mention that $H'_{8B}$ also has a critical phase. In the following paragraphs, we will show that $H'_{8B}$ indeed has vanishing $DOS_F$ when $A \neq 1$, and $A$ will determine the range of the critical phase. Hence, we obtain a convincing critical metal phase in $H'_{8B}$.

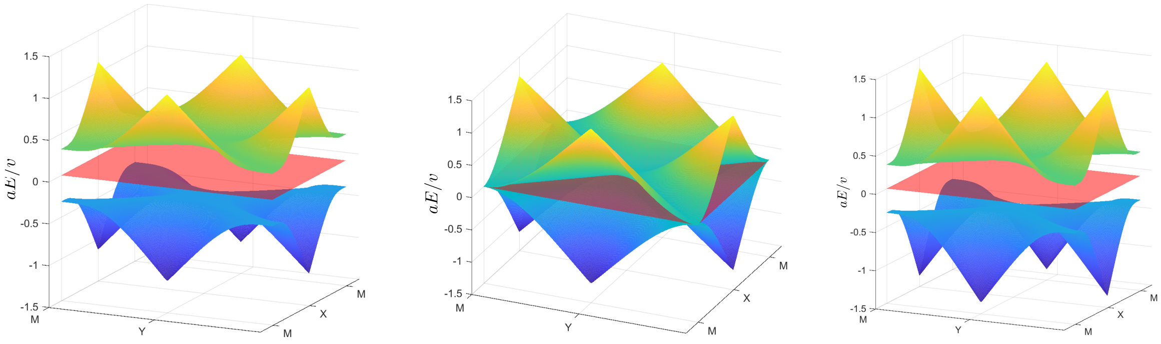


(a)$\tilde{t} = 0.4\pi v/a$ (b)$\tilde{t} = 0.5\pi v/a$ (c)$\tilde{t} = 0.6\pi v/a$

Supplementary Fig. 26. 3D plot of the band structure of $H'_{8B}$ with $A = 1$ (merely remove square diagonal hopping from $H_{8B}$) near the Fermi level $E_F = 0$ (the red plane). The two bands closest to the Fermi level touch at $\tilde{t} = \pi v/2a$ and are separated for other $\tilde{t}$. Such an evolution of band structure ($A = 1$) can be viewed as a special case of the evolution in Supplementary Fig. 28 where $A \neq 1$.

In order to obtain the band structure of $H'_{8B}$, we introduce the Fourier transform:

$$c_{\vec{q}}(w,\alpha) = \frac{1}{N}\sum_{nm} e^{-i\vec{q}\cdot(\vec{R}_{nm}+\alpha\,\vec{t}_w+\vec{\Delta})} c_{n,m}(w,\alpha)$$
$$\vec{t_1} = (0,0) \qquad \vec{t_2} = (0,a) \qquad \vec{t_3} = (-a,a) \qquad \vec{t_4} = (-a,0) \qquad \vec{\Delta} = (a,a) \tag{84}$$

where $\alpha\vec{t_w} + \vec{\Delta}$ are the relative positions of the corner states in each unit cell. We obtain the simplified tight-binding Hamiltonian $H'_{8B}$ in reciprocal space:

$$H'_{8B,0} = \sum_{\vec{q}}\sum_{\alpha}\sum_{w_1 w_2} (\widetilde{B}_\alpha)_{w_1,w_2}\, c^\dagger_{\vec{q}}(w_1,\alpha)c_{\vec{q}}(w_2,\alpha), \qquad H'_{8B,1} = \sum_{\vec{q}}\sum_{\alpha}\sum_{w} \tilde{t}\; c^\dagger_{\vec{q}}(w,\alpha)c_{\vec{q}}(w,\bar{\alpha}) \tag{85}$$

$$\widetilde{B}_\alpha = \frac{\pi v}{4a}\begin{pmatrix} 0 & -(1-\alpha Ai)e^{i\alpha q_y} & 0 & -(1+\alpha Ai)e^{-i\alpha q_x} \\ -(1+\alpha Ai)e^{-i\alpha q_y} & 0 & -(1-\alpha Ai)e^{-i\alpha q_x} & 0 \\ 0 & -(1+\alpha Ai)e^{i\alpha q_x} & 0 & -(1-\alpha Ai)e^{-i\alpha q_y} \\ -(1-\alpha Ai)e^{i\alpha q_x} & 0 & -(1+\alpha Ai)e^{i\alpha q_y} & 0 \end{pmatrix} \tag{86}$$

Supplementary Fig. 28 shows the two bands closest to the Fermi level with $A = 1.2$ and various $\tilde{t}$. Comparing 28 with Supplementary Fig. 23, we can see that removing the square diagonal hopping and introducing $A$ indeed vanish the density of state at the Fermi level while the picture of phase transition is similar. For $A > 1$, bands touch only at four Dirac points when $\pi v/2a\tilde{t} < A\pi v/2a$ and are separated for other $\tilde{t}$. These Dirac points appear at $\Gamma$ point when $\tilde{t} = \pi v/2a$, move along the $\Gamma - M$ line as $\tilde{t}$ increases, and merge at $M$ point when $\tilde{t} = A\pi v/2a$. Thus the system is metallic for $\pi v/2a\tilde{t} < A\pi v/2a$ and insulated for $\tilde{t} < \pi v/2a$ & $\tilde{t} > A\pi v/2a$. The number of Dirac points here is four, which seems different from the transition process of $H_{8B}$ that has eight. However, as shown in Supplementary Fig. 29, these two transition processes can continuously deform to each other by changing the dispersion of bands. During the deformation, four of the eight Dirac points become more and more close to the $\Gamma$ point; finally, they merge at the $\Gamma$ point, leaving the other four Dirac points moving toward the $M$ point. Hence, we can view $H'_{8B}$ as having eight Dirac points, four of which have zero life spans. Additionally, the Dirac point evolution of $H_{8B}$, $H'_{8B}$ and their intermediate states are equivalent to one positive Dirac point going clockwise around the $X$ point one circle. Hence, we regard the transition processes of $H_{8B}$ and $H'_{8B}$ to be equivalent.

If $A < 1$, the situation is reversed: Dirac points exist when $A\pi v/2a < \tilde{t} < \pi v/2a$ and appear (merge) at $M$ ($\Gamma$) point when $\tilde{t} = A\pi v/2a$ ($\tilde{t} = \pi v/2a$). It is straightforward to see that this process can also connect to $H_{8B}$ by deforming the band dispersion in an opposite way, thus also equivalent to $H_{8B}$. If $A = 1$, bands touch at two lines that form an"X-shape" when $\tilde{t} = \pi v/2a$ and are separated for other $\tilde{t}$ (see Supplementary Fig. 26). We call the region where the bands touch at the Fermi level as the semi-metal region. Only in the semi-metal region, a critical metal can arise. $\det\left[H'_{8B}(\vec{q})\right] = 0$ shows that the reciprocal coordinates of Dirac points are given by

$$|q_{0x}| = |q_{0y}| = \frac{1}{2a}\arccos\left[\frac{A^2-\tilde{\gamma}^4}{\tilde{\gamma}^2(A^2-1)}\right] \qquad \tilde{\gamma} = \frac{a\tilde{t}}{\pi v/2} \tag{87}$$

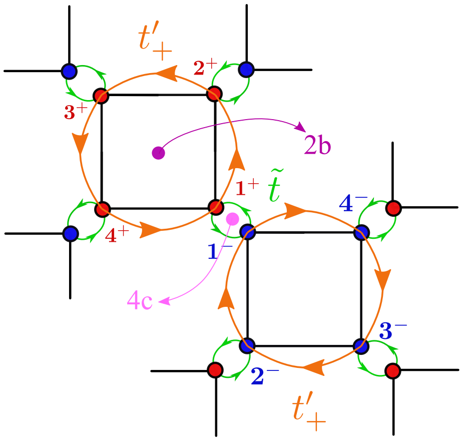


Supplementary Fig. 27. Unit cell of $H'_{8B}$. The orange and green curves with arrows illustrate the hoppings of Eq. (85). Inside one square, $t'_+ = -(1 + Ai)\pi v/4a$ denotes the modified square edge hopping. And the direction of $t'_+$ is indicated by the orange arrows. $\tilde{t}$ denotes the hopping between squares. The red & blue circles and decorated numbers $w^\pm$ ($w = 1, 2, 3, 4$) indicate the positions of corner states $c_{n,m}(w, \pm)$. 4c and 2b indicate the (representative of) corresponding Wyckoff positions.

We should also mention that the BR transition of $H'_{8B}$ is the same as that of $H_{8B}$. The four bands forming $\mathrm{A}''_c\uparrow\mathrm{G}$ are also connected, even though $\mathrm{A}''_c\uparrow\mathrm{G}$ is decomposable for general cases. Thus, the k·p analysis of the Dirac points presented in Supplementary Sec. IV D also applies to the four Dirac points here because the gap closes at $\Gamma$ and $M$ involve the same irreps as in the $H_{8B}$ model. Hence, two $C_{4z}$-related Dirac points here also have opposite chiralities.

An additional chiral symmetry in the clean limit arises from the simplification: The Hamiltonian in momentum space anti-commutes with the diagonal matrix $C = \tau_z \otimes \xi_0 \otimes \sigma_z$, where $\tau_z$ is a Pauli matrix representing the two Chern blocks, $\xi_0$ and $\sigma_z$ are Pauli matrices representing the four corner state basis within each Chern block. More concretely, $\xi_0 \otimes \sigma_z$ is a rank-4 diagonal matrix that multiplies 1 to corners $w = 1, 3$ and $-1$ to $w = 2, 4$. Nevertheless, the disorder we considered (Supplementary Sec. IV F) will break it. We also perform numerical works in Supplementary Sec. I C with $E_F \neq 0$ where the chiral symmetry *on average* is broken (see Supplementary Fig. 10) and the critical phase still exist.

To end this subsection, we summarize all the models described above. We start from the Manhattan network model (Eq. (36)), which can describe the percolation process on chiral edge states (Fig. 1(a), (b)). We notice that the eigenstates of the network model are circular chiral states when $\theta = \pm\pi/2$. Hence, we use the circular states of $\theta = -\pi/2$ as a basis and obtain a un-truncated lattice model (Eq. (63)). The network and the un-truncated lattice model describe the same system from different bases, and we no longer distinguish them hereafter unless otherwise stated. Then we take a low truncation on the lattice model and obtain the eight-band lattice model $H_{8B}$. We claim that $H_{8B}$ with $E_F = 0$ reproduces the low energy physics of the network model with $E_F = \pi v/4a$ and show evidence for this. $H_{8B}$ can be described by both (truncated) standing wave basis (Eq. (56)) and corner state basis (Eq. (64)). According to Supplementary Sec. I, $H_{8B}$ with $E_F \approx 0$ has a similar critical metal phase to the network model. However, when $H_{8B}$ is critical, the corresponding band structure in the clean limit has a finite $DOS_F$ (Supplementary Fig. 23) which makes the critical phase harder to distinguish from a very large localization length Anderson insulator (see Supplementary Sec. V A for an explanation). In order to obtain a more convincing critical phase, we modify $H_{8B}$ to $H'_{8B}$ which also has a critical metal phase when $E_F = 0$(Supplementary Sec. I C) while the corresponding $DOS_F = 0$ (Supplementary Fig. 28). As explained in Supplementary Sec. IV F, the criticality on the network model can be directly related to those in random flux models [14–16]. On the other hand, the criticality of $H_{8B}$ and $H'_{8B}$ is due to the $w_2$ transition between two OAIs. The analysis in the above subsections (Supplementary Sec. IV A, Supplementary Sec. IV B, IV C, IV E) offers a quantitative mapping between these criticalities. In this paper, we focus on specific Fermi levels. We choose $E_{F-8B} = E'_{F-8B} = 0$ for $H_{8B}$ and $H'_{8B}$ because they sit in the middle of the eight bands. We choose $E_{F-N} = \pi v/4a$ for the network model because its band structure (in the metal limit) near $E_{F-N}$ is similar to that of $H_{8B}$ near $E_{F-8B}$ (Supplementary Fig. 23). However, additional numerical works show that these choices of Fermi levels are not essential for the critical metal: first, as explained in Supplementary Sec. V B, $E_{F-N}$ is not essential because the localization behavior of a network model is blind to the Fermi level; second, see Supplementary Fig. 9 and Supplementary Fig. 10, $E_{F-8B}$ and $E'_{F-8B}$ are not essential because $H_{8B}$ and $H'_{8B}$ still have critical phases at $E_F \neq 0$ as long as the OAI limits are intact.

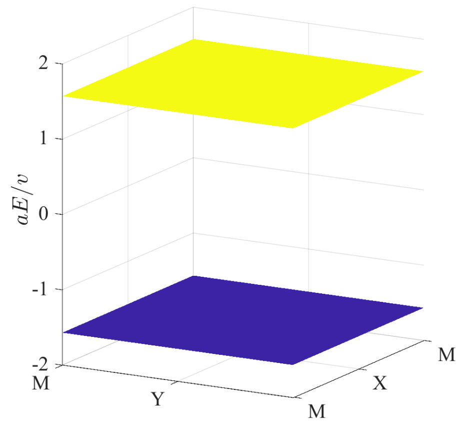


(a)$\tilde{t} = 0$

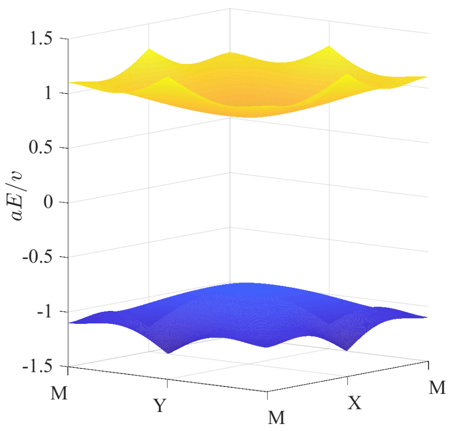


(b)$\tilde{t} = \pi v/4a$

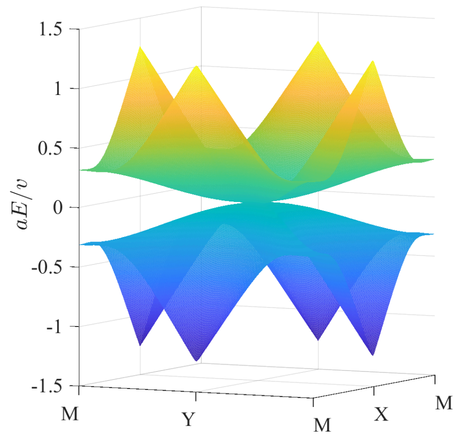


(c)$\tilde{t} = \pi v/2a$

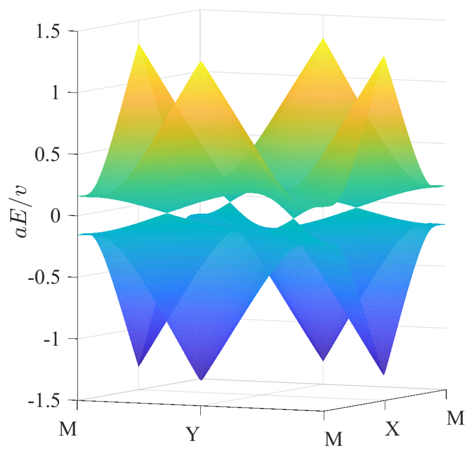


(d)$\tilde{t} = 1.1\pi v/2a$

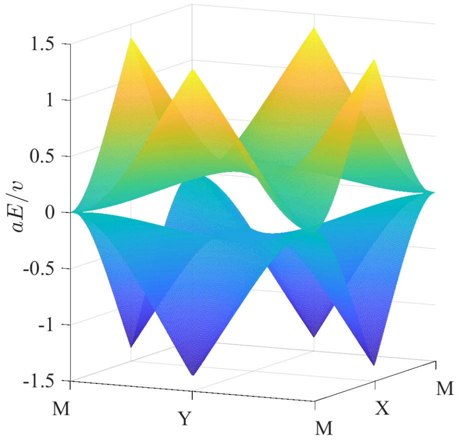


(e)$\tilde{t} = 1.2\pi v/2a$

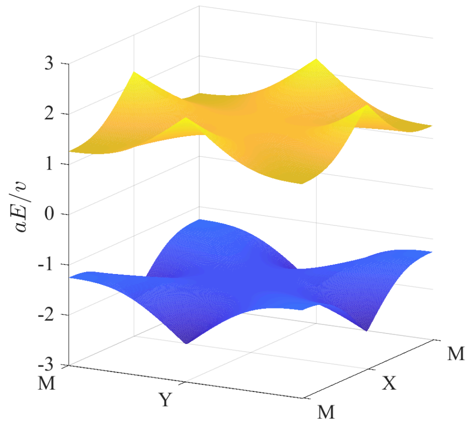


(f)$\tilde{t} = \pi v/a$

Supplementary Fig. 28. 3D Band structure of simplified lattice model near the Fermi level ($A = 1.2$). (a)∼(b) correspond to the insulate limit with $C_{4z}$ center as the OAI center. (c)∼(e) correspond to the transition region, where four Dirac points appear and merge at $\Gamma$ and $M$ points, respectively. (f) corresponds to the insulate limit with $C_{2z}T$ center as the OAI center.

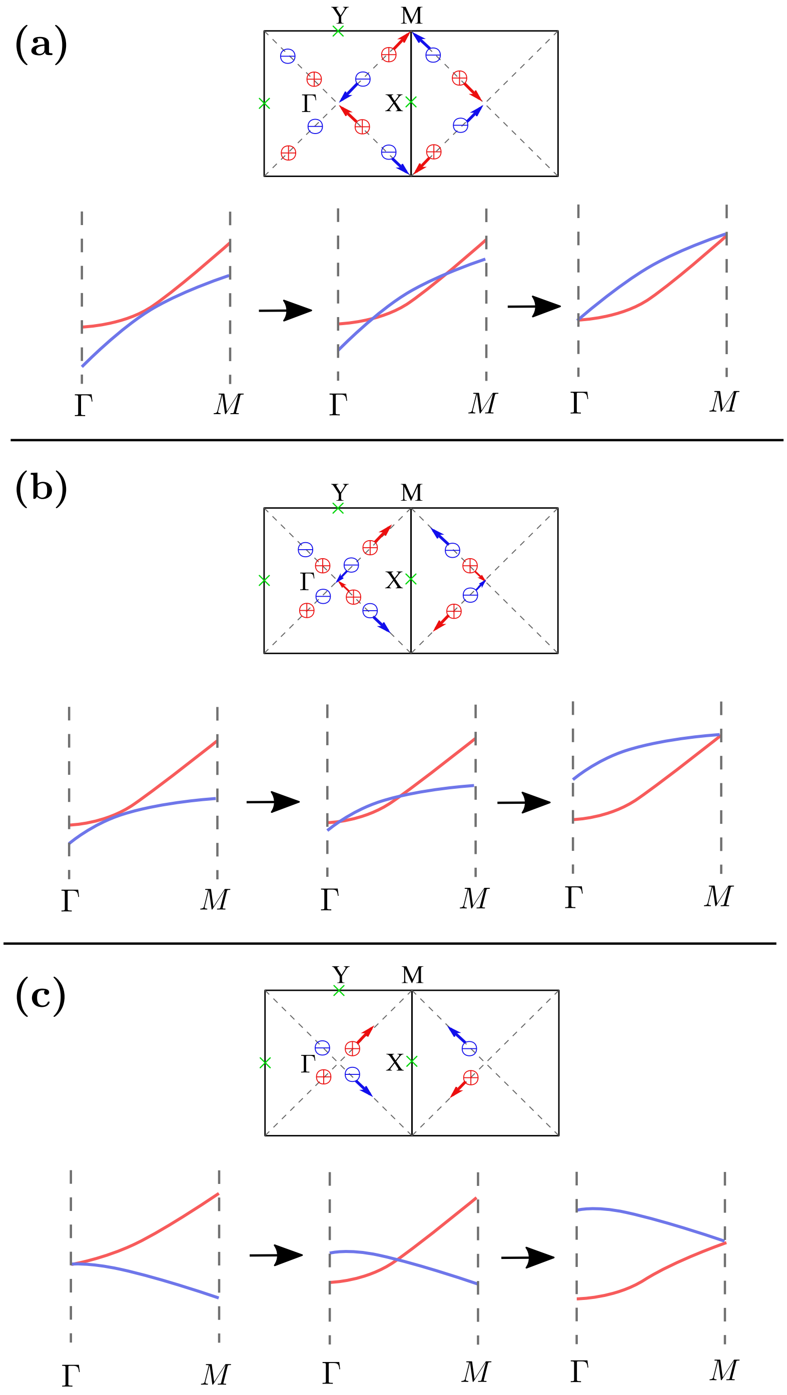


Supplementary Fig. 29. Continuous deformation from the transition process of $H_{8B}$ to that of $H'_{8B}$. (a) The evolution of Dirac points of $H_{8B}$ and the corresponding band structure evolution according to Supplementary Fig. 22(d)∼22(e). Dirac points appear approximately at the middles of the $\Gamma$-$M$ lines. (b) An intermediate state between $H_{8B}$ and $H'_{8B}$. Dirac points appear at the positions that are closer to the $\Gamma$ point. (c) The evolution of Dirac points of $H'_{8B}$ and the corresponding band structure evolution according to Supplementary Fig. 28. All these transition processes are equivalent to one positive Dirac point going around the $X$ point clockwise once.

### F. Disorder potential

Previous sections do not involve the disorder explicitly. In order to study the localization, we should add disorder to our models (especially the transfer matrices in Supplementary Sec. V). As mentioned in Supplementary Sec. III A, in order to depict the percolation process in Fig. 1(a), (b), the disorder should be the random size of the Chern blocks. Since an electron will accumulate phase when propagating along a chiral wire, the random size of Chern blocks will manifest in the random phases along the edges of Chern blocks. For the network model, these random phases can be viewed as random fluxes through the colored squares in Supplementary Fig. 16(a). And the flux disorder can be directly added to the transfer matrix (see Supplementary Sec. V B). In the un-truncated lattice model (Eq. (55)), the Chern blocks are also represented by the red and blue squares (see Supplementary Fig. 21(a)), and the random fluxes can be realized by random vector potentials around these squares. Therefore, under the circular chiral basis, *i.e.*, $\psi_{n,m}(x_\alpha,\alpha)$ in Eq. 57, $H_{N,0}$ (in Eq. (55)) and the disorder potential can be written as

$$H_0 + H_{rand} = \sum_{nm}\sum_{\alpha}\sum_{s=1}^{4}\int_{(s-1)a}^{sa} \mathrm{d}x\, \psi^\dagger_{n,m}(x,\alpha)(-i\alpha v\partial_x - \alpha v\mathcal{A}_{nm\alpha s})\psi_{n,m}(x,\alpha), \tag{88}$$

where $s = 1,2,3,4$ is the index of the four edges of one square. $(n,m,\alpha,s)$ indicates the $s$th side, *i.e.*, $x_\alpha \in [(s-1)a, sa)$, of the $\alpha$ square in the $(n,m)$ cell. $\mathcal{A}_{nm\alpha s}$ is the random vector potential on the associated edge, and it is assumed to be uniform within each edge. In this paper, the random vector potentials on different edges are independent.

Then, we project $H_{rand}$ in Eq. (88) into the space of truncated lattice model $H_{8B}$ and $H'_{8B}$, *i.e.*, the space spanned by standing waves defined in Eq. (56) with $K = 0,\ 1,\ 2,\ 3$. And we have

$$\begin{aligned} H_{8B,rand} &= \sum_{nm}\sum_{\alpha s}\sum_{K_1K_2}\frac{1}{4a}\int_{(s-1)a}^{sa}\mathrm{d}x \exp\left[i\alpha\frac{\pi(K_2-K_1)}{2a}x\right][-\alpha v\mathcal{A}_{nm\alpha s}]\phi^\dagger_{n,m}(K_1,\alpha)\phi_{n,m}(K_2,\alpha) \\ &= \sum_{nm}\sum_{\alpha s}[-\alpha v\mathcal{A}_{nm\alpha s}]\sum_{K_1K_2}\left[\frac{\delta_{K_1,K_2}}{4} + (1-\delta_{K_1,K_2})\frac{(\alpha i)^{s(K_2-K_1)}(1-(\alpha i)^{(K_1-K_2)})}{2\pi\alpha i(K_2-K_1)}\right]\phi^\dagger_{n,m}(K_1,\alpha)\phi_{n,m}(K_2,\alpha), \end{aligned} \tag{89}$$

where $K_1, K_2$ are limited to $0,1,2,3$. We define a 4-by-4 matrix

$$(C_{\alpha s})_{I_1,I_2} = \begin{cases} \dfrac{(\alpha i)^{s(I_2-I_1)}(1-(\alpha i)^{(I_1-I_2)})}{2\pi\alpha i(I_2-I_1)} & (I_1 \neq I_2) \\ \dfrac{1}{4} & (I_1 = I_2) \end{cases} \quad (I_1, I_2 = 1,2,3,4). \tag{90}$$

Since the corner state basis describes the system in a more local way and the transfer matrix used for further numerical calculations (see Supplementary Sec. V C) is simpler on this basis. We now represent $H_{8B,rand}$ on the corner state basis. Making use of Eq. (64), we have

$$\begin{aligned} H_{8B,rand} &= \sum_{nm}\sum_{\alpha s}[-\alpha v\mathcal{A}_{nm\alpha s}](\vec{c}_{nm\alpha})^\dagger U_{\bar\alpha}C_{\alpha s}U_\alpha\vec{c}_{nm\alpha} \\ &= \sum_{nm}\sum_{\alpha w_1 w_2}\left[\sum_{s} -\alpha v\mathcal{A}_{nm\alpha s}D_{\alpha s}(w_1,w_2)\right]c^\dagger_{n,m}(w_1,\alpha)c_{n,m}(w_2,\alpha). \end{aligned} \tag{91}$$

Here, $\vec{c}_{nm\alpha}$ is a 4-by-1 column vector of $c_{n,m}(w,\alpha)$, $(w = 1,2,3,4)$. $U_\alpha$ is the 4-by-4 transform matrix defined in Eq. (64) and $\bar\alpha = -\alpha$. One should not confuse the transform matrx $U_\alpha$ with the random vector potential $\mathcal{A}_{nm\alpha s}$. $D_{\alpha s}$ is a 4-by-4 matrix

with indices $w_1, w_2$. More concretely,

$$
\begin{aligned}
D_{-1} &= \frac{1}{12\pi}\begin{pmatrix} 8+3\pi & 3(1+i) & 4i & 7(i-1) \\ 3(1-i) & -8+3\pi & 1+i & 4i \\ -4i & 1-i & -8+3\pi & 3(1+i) \\ -7(1+i) & -4i & 3(1-i) & 8+3\pi \end{pmatrix}, \\
D_{-2} &= \frac{1}{12\pi}\begin{pmatrix} -8+3\pi & 1+i & 4i & 3(1-i) \\ 1-i & -8+3\pi & 3(1+i) & -4i \\ -4i & 3(1-i) & 8+3\pi & -7(1+i) \\ 3(1+i) & 4i & 7(i-1) & 8+3\pi \end{pmatrix}, \\
D_{-3} &= \frac{1}{12\pi}\begin{pmatrix} -8+3\pi & 3(1+i) & -4i & 1-i \\ 3(1-i) & 8+3\pi & -7(1+i) & -4i \\ 4i & 7(i-1) & 8+3\pi & 3(1+i) \\ 1+i & 4i & 3(1-i) & -8+3\pi \end{pmatrix}, \\
D_{-4} &= \frac{1}{12\pi}\begin{pmatrix} 8+3\pi & -7(1+i) & -4i & 3(1-i) \\ 7(i-1) & 8+3\pi & 3(1+i) & 4i \\ 4i & 3(1-i) & -8+3\pi & 1+i \\ 3(1+i) & -4i & 1-i & -8+3\pi \end{pmatrix},
\end{aligned}
\tag{92}
$$

and $D_{+s} = D^*_{-s}$ $(s = 1, 2, 3, 4)$.

This is a quite complicated random potential ($\sum_s \mathcal{A}_{nm\alpha s} D_{\alpha s}$) because all its matrix elements are nonzero. Nevertheless, we should point out that the most prominent terms, whose norm is at least as $\frac{8+3\pi}{7\sqrt{2}} \approx 2$ times large as other terms, locate in the diagonal line. This inspired us to only keep the on-site (chemical potential) disorder for $H_{8B}$ & $H'_{8B}$. For simplicity and efficiency, we would like to ignore the correlations among on-site random potentials at different corners further. It turns out that uncorrelated on-site disorder is sufficient to reproduce the (de)localization behaviors of the network model. Benchmarking and test calculations (see Supplementary Fig. 30 for an example) show no qualitative difference between using uncorrelated on-site disorder only and using the full disorders in Eq. (91). Therefore, we take the evenly distributed, uncorrelated on-site disorder (in the range $[-W/2, W/2]$) for $H_{8B}$ and $H'_{8B}$.

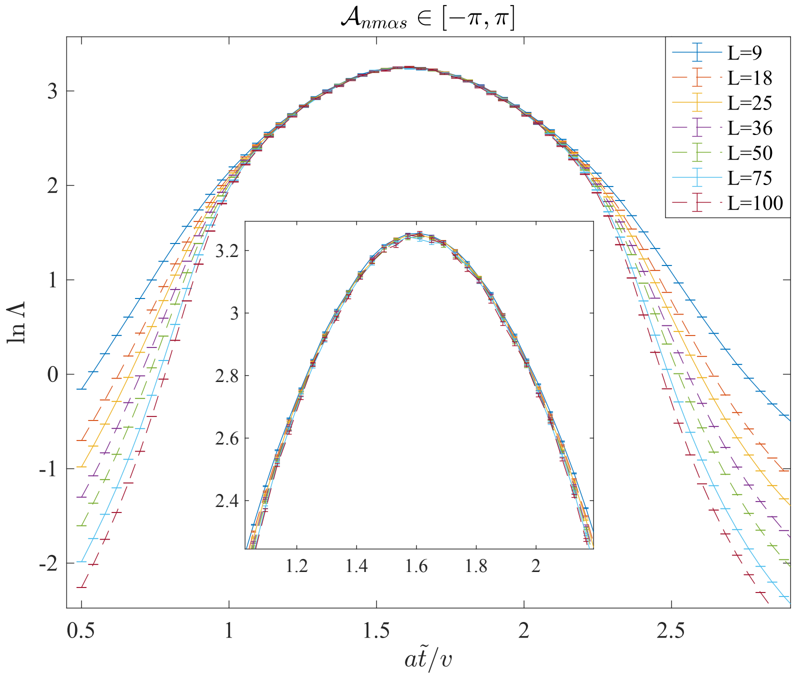


Supplementary Fig. 30. The normalized localization length $\Lambda$ versus $\tilde{t}$ in $H_{8B}$ (Eq. (66)) with all the random potentials in Eq. (91). The random vector potential $\mathcal{A}_{nm\alpha s}$ evenly distributes in $[-\pi, \pi]$ to consist with the network model. The longitudinal length is $M = 5 \times 10^7$ and the data precision ($\sigma_\Lambda/\Lambda$) has reached 0.7%. The inset is the zoomed critical region.

## V. Quasi-1D localization length and Transfer matrix method

### A. General introduction

A commonly used physical quantity in researches of localization is the *quasi-1D localization length* $\rho_{\rm q-1D}$. It is defined on a 2D/3D sample prepared in quasi-1D shape, *e.g.*, a long thin cylinder with $L_{\rm axial} \gg L_{\rm radius}$. $\rho_{\rm q-1D}$ reflects the decaying rate of eigenstates in the quasi-1D direction, *e.g.*, the axial direction of a long thin cylinder. Since any 1D system will be localized under nonzero disorder strength, $\rho_{\rm q-1D}$ will always be finite except for a perfectly clean sample. Localization of the original 2D/3D system (in 2D/3D shape) can be determined by a scaling analysis of the dimensionless quasi-1D localization length $\Lambda = \rho_{\rm q-1D}/L$, where $L$ is the transversal size of the quasi-1D sample (see Supplementary Fig. 31). We denote the localization length of a normal shaped (scales of different directions are similar) and sufficiently large sample as $\rho$. For a metallic system, $\rho$ in a (normal shaped and sufficiently large) sample is much larger than the sample size. Thus, $\rho_{\rm q-1D}(L)$ increases faster than $L$, *i.e.*, $\Lambda(L) \to \infty$ in the limit $L \to \infty$. For an insulating system, $\rho$ is finite in a (normal shaped and sufficiently large) sample, so that $\rho_{\rm q-1D}$ will converge to $\rho$ when $L \gg \rho$, *i.e.*, $\Lambda(L) \to 0$ as $L \to \infty$. In practice, we identify the region where $\Lambda(L)$ monotonically increases as the metallic phase and the region where $\Lambda(L)$ monotonically decreases as the localized phase. If a system contains both localized and extended phases in some parameter space, there will be a critical region (usually a point) in the parameter space where $\Lambda(L)$ is independent on (sufficiently large) $L$. Note that such an analysis can only demonstrate the localization behavior (of a normal shaped sample) along one direction. For instance, in a 2D system, if we take the quasi-1D direction along the x axis (and the transversal direction will be the y direction), the scaling analysis of $\rho_{\rm q-1D}(L_y)$ will tell us the localization behavior (of a normal shaped sample) along the x direction. In principle, a normal shaped 2D system can be localized in the x direction while extended in the y direction. If one cannot rule out this possibility, it is necessary to do the afore-mentioned analyses of $\rho_{\rm q-1D}$ both along the x and y directions. Nevertheless, if one can rule out this possibility, *i.e.*, the normal shaped system is localized or extended in the x and y directions simultaneously, then analysing $\rho_{\rm q-1D}$ along one direction is enough. To see this, we can denote the angle between two directions $\hat{o}_1, \hat{o}_2$ as $\langle \hat{o}_1, \hat{o}_2 \rangle$. For a state on a normal shaped 2D sample, if it is extended in direction $\hat{o}_1$, it should also be extended in $\hat{o}_2$ as long as $\langle \hat{o}_1, \hat{o}_2 \rangle < \pi/2$. Notice that $\min\{\langle \hat{o}_1, \hat{x} \rangle, \langle \hat{o}_1, \hat{y} \rangle\} < \pi/2$ for any direction $\hat{o}_1$. Hence, a normal shaped 2D system extended (localized) in both the x and y directions should be extended (localized) in any direction. In such a case, scaling analyses of $\rho_{\rm q-1D}$ along different quasi-1D directions are similar, since the localization behaviors (of a normal shaped sample) in different directions have no qualitative difference. Our models all have $C_4$ symmetries. Hence, the systems are extended (localized) in the x and y directions simultaneously, and we can choose the quasi-1D directions for numerical convenience without resulting in qualitative difference.

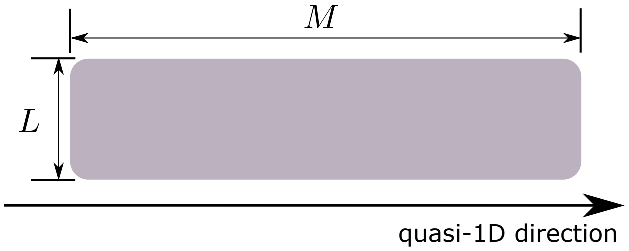


Supplementary Fig. 31. Illustration of a quasi-1D sample with transversal size $L$ and longitudinal size $M \gg L$. The quasi-1D localization length $\rho_{\rm q-1D}$ based on the Lyapunov exponents is calculated on such a sample.

However, it is questionable to apply the above strategy on a localized system with $\rho \gg$ *practical* $L$ since the finite size effect is prominent there. For such a system, a localized phase can behave as a critical phase for practical $L$. This is not the case for the network model since its critical phase begins with $\Lambda < e^2 \approx 7$ (Supplementary Fig. 1). However, it seems to be the case of $H_{8B}$ and $H'_{8B}$. Because $\Lambda \approx e^3 \approx 20$ in their critical metal phases (Supplementary Fig. 4 & 5). To rule out this possibility, we calculate $\Lambda$'s in the critical phases for a wide range of transversal sizes where $L_{\max} \approx 50 L_{\min}$ (see Supplementary Fig. 8) and no significant decline of $\Lambda(L)$ has been observed. Second, if the critical phases we observed in Supplementary Fig. 4 & 5 are actually localized phases with large localization lengths, they will eventually become localized as $L \to \infty$. For finite $L$, these fake critical phases should shrink as $L$ increases. However, according to Supplementary Fig. 4 & 5, the boundary between the localized phase and critical phase does not change as $L$ increases. In addition, we calculate the local Chern marker in Supplementary Sec. I D, which also demonstrate the delocalization of $H_{8B}$ and $H'_{8B}$ in the critical phases. (We leave the detailed explanation in Supplementary Sec. I D.) Based on these observations, we claim that the critical phase in $H_{8B}$ and $H'_{8B}$ is not due to the finite size effects and will persist in the thermodynamic limit.

The transfer matrix method [17] is a widely used numerical approach in calculating $\rho_{\rm q-1D}$. Although it has different formulae for (general) network and lattice models, the basic ideas are the same. A quasi-1D sample is divided into layers with normals

along the quasi-1D direction. The amplitudes of an energy-eigenstate on different layers are related by the Schrodinger equation. Note that the layer division on a concrete system should be specially designed for numerical convenience (we will see this in Supplementary Sec. V B & V C). A ($2s \times 2s$ shaped) transfer matrix $T_n$ in general is a transformation from the amplitudes on the $(\mathrm{l}-\mathrm{r}_1)\mathrm{th} \sim (\mathrm{l}+\mathrm{r}_2-1)\mathrm{th}$ layers to those on the $(\mathrm{l}-\mathrm{r}_1+1)\mathrm{th} \sim (\mathrm{l}+\mathrm{r}_2)\mathrm{th}$ layers. Here $s \in \mathbb{N}^+$ is proportional to the number of degrees of freedom in one layer. And $r_1,\, r_2 \in \mathbb{N}^+$ represent that only the $(\mathrm{l}-\mathrm{r}_1)\mathrm{th} \sim (\mathrm{l}+\mathrm{r}_2)\mathrm{th}$ layers can hop/propagate (in one step) to the $\mathrm{l}\,\mathrm{th}$ layer. The values of $r_1,\, r_2$ depend on the hopping of concrete models. In our models, $r_1 = r_2 = 1$. Nevertheless, for generality, we keep $r_1,\, r_2$ in general discussions about the transfer matrix method. From this point of view, network model is a special case. Since the transfer matrix of a (general) network model is determined by the transmission matrix, the amplitudes in the $(\mathrm{l}+1)\mathrm{th}$ layer depend only on the $\mathrm{l}\mathrm{th}$ layer. Hence, $r_1 = r_2 = 1$ for general network models (not only ours). We will see this more clearly in Supplementary Sec. V B. For general lattice models, that is not the case, *i.e.*, $r_1$ and $r_2$ can take arbitrary finite non-negative integer values.

For a quasi-1D system containing $M$ layers (we also use $M$ to represent the longitudinal size), we can define a consecutive product $O_M = \prod_{n=1}^{M} T_n$ that transforms the amplitudes on the first $r_1 + r_2 + 1$ layers to the last $r_1 + r_2 + 1$ layers. $\rho_{\mathrm{q-1D}}$ can be extracted from $O_M$. Because of the disorder, some elements in $T_n$ are random variables. According to the Oseledec's theorem, the limit $P = \lim_{M\to\infty}(O_M^\dagger O_M)^{1/2M}$ exists and has eigenvalues $\{\exp(\nu_1),\, \exp(-\nu_1), \ldots \exp(\nu_s),\, \exp(-\nu_s)\}$ where $\nu_i \geq \nu_{i+1} \geq 0,\; i = 1, 2 \ldots s$. These (positive) exponents are so-called *Lyapunov exponents* (LEs). The definition of $P$ indicates that an eigenvector $\vec{\eta}_i$ of $P$ with eigenvalue $\exp(-\nu_i)$ satisfies $\|O_M \vec{\eta}_i\|^2 = \vec{\eta}_i^\dagger (O_M^\dagger O_M)\, \vec{\eta}_i = \vec{\eta}_i^\dagger [(O_M^\dagger O_M)^{1/2M}]^{2M}\, \vec{\eta}_i \approx \vec{\eta}_i^\dagger P^{2M}\, \vec{\eta}_i = \|\exp(-M\nu_i)\vec{\eta}_i\|^2$ for sufficiently large M. Therefore, the smallest LE $\nu_s$ determines the decaying rate of energy-eigenstates (along the quasi-1Dc direction) since any energy-eigenstate is a superposition of eigenvectors of $P$ with eigenvalues $\exp(-\nu_i)$ (the amplitudes cannot grow exponentially hence $\exp(\nu_i)$ is excluded). In this paper, we define $\rho_{\mathrm{q-1D}} = 1/\nu_s$.

It is worth introducing some numerical details briefly. The definition of $P$ requires us to first calculate $O_M^\dagger O_M$ whose eigenvalues are $\exp(\pm 2M\nu_i)$ when $M \to \infty$. (For our calculations, we usually take $M = 10^6 \sim 10^8$.) When $M$ is sufficiently large, $\exp(M\nu_i) \gg \exp(M\nu_j)$ if $\nu_i > \nu_j$, and most of the computational resources are consumed by the large LEs. The small LEs will acquire vast round-off errors since they are stored in the low digits. Unfortunately, we are concerned with the smallest LE $\nu_s$. To circumvent such errors, the practical numerical method should extract information after every several $T$'s are multiplied to $O$. We point out without proof [17] that

$$
\begin{aligned}
&O_M = UR \\
&\nu_i = \lim_{M\to\infty} \frac{\ln(R)_{i,i}}{M}\;.
\end{aligned}
\tag{93}
$$

The first line is the QR decomposition of $O_M$, and the second line means that the LEs are determined by diagonal elements in the upper triangular matrix $R$. Suppose there are two upper triangular matrices $R_1,\, R_2$, it is direct to prove that $(R_1R_2)_{i,i} = (R_1)_{i,i}(R_2)_{i,i}$. Because of this property, we do not have to decompose the entire $O_M$ after all $T$'s are multiplied. Instead, QR decomposition can be executed after every $q$ $(2 \sim 10)$ $T$'s are multiplied to $O$. After each decomposition, diagonal elements in $R$ are stored, and the next $q$ $T$'s will be multiplied to $U$. Repeat this treatment one will obtain $M/q$ arrays of diagonal elements $(R_j)_{i,i},\; (j = 1, 2 \ldots M/q)$ in the end. We can rewrite the second line of Eq. (93) as

$$
\nu_i = \lim_{M/q\to\infty} \frac{1}{M/q - n_0} \sum_{j=n_0+1}^{M/q} \frac{\ln(R_j)_{i,i}}{q}
\tag{94}
$$

In this formula, the vast round-off error ($\sim \exp(M(\nu_1 - \nu_s))$) is replaced by a marginal round-off error ($\sim \exp(q(\nu_1 - \nu_s))$). We have excluded the first $n_0$ (set to 10) groups ($n_0 q$ layers) in practical calculations to avoid possible boundary effects.

Another advantage of Eq. (94) is that we can estimate the numerical precision of LEs. One can view Eq. (94) as an average of $\ln(R_j)_{i,i}/q$ over $M/q - n_0$ samples. Therefore, we can estimate the error of the sample average by unbiased estimation and use it to control the precision. Prudent readers may suspect the independence between "data points" $\ln(R_j)_{i,i}/q$ for different $j$ when $q$ is small. In practice, we will group up $r \sim 10^1$ "data points" and view the $((M/q - n_0)/r)$ groups independent to each other.

### B. Transfer matrices of the network model on the Manhattan lattice

In this subsection, we will introduce the concrete formulae of transfer matrices in the network model defined in Eq. (21) & (22). Supplementary Fig. 32 shows some details of transfer matrices in the network model. We choose $x + y$ as the quasi-1D direction since only in this direction the layer division and inter-layer relation are simple. The green octagons marked by $S_1 \cdots S_4$ indicate four kinds of scattering nodes, respectively. The green dashed lines indicate the layer division along the quasi-1D direction, *i.e.*, chiral edges sitting inside two adjacent green dashed lines belong to one layer. Each square edge is

represented by two composite indices $(\mathcal{X},\chi)$ & $(\mathcal{Y},\nu)$, where $\mathcal{X}\in\mathbb{Z}$ is the square index along the quasi-1D direction, $\mathcal{Y}\in\mathbb{Z}$ is the square index along the transversal direction, and $\chi,\nu=0,1$ further distinguish four edges in one square. As shown in Supplementary Fig. 32(a), three successive layers are labeled by $(\mathcal{X},\chi)=(\eta,0)$, $(\eta,1)$, $(\eta+1,0)$, respectively. Within each layer, four successive chiral edges are labeled by $(\mathcal{Y},\nu)=(\xi,0)$, $(\xi,1)$, $(\xi+1,0)$, $(\xi+1,1)$, respectively. We take the convention that $(-1)^{\mathcal{X}+\mathcal{Y}}=-1$ corresponds to the blue squares and $(-1)^{\mathcal{X}+\mathcal{Y}}=1$ corresponds to the red ones. Hence, in Supplementary Fig. 32(a), $\eta+\xi$ is even. The disorder is the collection of random phases accumulated along the edges, which reflects the random local fluxes (Supplementary Sec. IV F).

There are two parts that contribute to the transfer matrix. The first part is the random phase accumulated (when moving from left to right) on each chiral mode, denoted as $\exp[i\vartheta(\mathcal{X},\chi,\mathcal{Y},\nu)]$. The golden arrows in Supplementary Fig. 32(a) indicate the directions along which an electron will acquire the random factor $\exp[\vartheta(\eta,0,\xi,0)]$ or $\exp[\vartheta(\eta,0,\xi,1)]$. We take completely random phases $\vartheta$, which uniformly distribute in $[-\pi,\pi]$ and set $\vartheta$ on different edges independent to each other. In principle, we should include a third part that comes from free propagation $e^{\pm iEa/v}$ along chiral edges. (Inside the chiral edges, we have $iv\partial\psi=E\psi$, *i.e.*, the eigenstate behaves like a plane wave with wave vector $E/v$. Also recall that the length of an edge is $a$). However, since we uniformly take values of random phases $e^{i\vartheta}$ in entire $U(1)$, the free propagation factor $e^{\pm iaE/v}$ becomes irrelevant and can be omitted. (This is also true for generic network models with uniform $U(1)$ random phases. Hence, localization behavior of a network model with uniform $U(1)$ random phases is independent on the Fermi level. In common sense, the Fermi level is important since the system will become a band insulator/metal if we put the Fermi level inside a gap/band. For a network model with uniform $U(1)$ random phases, the situation is modified: the model can be delocalized only if the spectrum has no gap everywhere.)

The second part is the scattering nodes. Although there are four kinds of scattering nodes $S_1\cdots S_4$, all of them can be rotated into Supplementary Fig. 16(b). See Supplementary Fig. 33 for the correspondence between $in/out$ channels in Supplementary Fig. 32(b) and the $a\sim d$ channels in Supplementary Fig. 16(b). For $S_1$, $in_{1,2}$ and $out_{1,2}$ correspond to $a$, $b$ and $d$, $c$ channels, respectively. For $S_2$, $in_{1,2}$ and $out_{1,2}$ correspond to $c$, $d$ and $b$, $a$ channels, respectively. For $S_3$, $in_{1,2}$ and $out_{1,2}$ correspond to $d$, $a$ and $c$, $b$ channels, respectively. For $S_4$, $in_{1,2}$ and $out_{1,2}$ correspond to $b$, $c$ and $a$, $d$ channels, respectively. Then, after some simple calculations, we can write the scattering effects on the basis of $in/out$ channels:

$$\begin{aligned}
S_1:\quad &\begin{pmatrix} out_1\\ out_2\end{pmatrix}=\begin{pmatrix} -i\sin\theta & \cos\theta\\ \cos\theta & -i\sin\theta\end{pmatrix}\begin{pmatrix} in_1\\ in_2\end{pmatrix}\\
S_2:\quad &\begin{pmatrix} out_1\\ out_2\end{pmatrix}=\begin{pmatrix} i\sin\theta & \cos\theta\\ \cos\theta & i\sin\theta\end{pmatrix}\begin{pmatrix} in_1\\ in_2\end{pmatrix}\\
S_3:\quad &\begin{pmatrix} out_1\\ out_2\end{pmatrix}=\begin{pmatrix} -i\tan\theta & \sec\theta\\ \sec\theta & i\tan\theta\end{pmatrix}\begin{pmatrix} in_1\\ in_2\end{pmatrix}\\
S_4:\quad &\begin{pmatrix} out_1\\ out_2\end{pmatrix}=\begin{pmatrix} i\tan\theta & \sec\theta\\ \sec\theta & -i\tan\theta\end{pmatrix}\begin{pmatrix} in_1\\ in_2\end{pmatrix}
\end{aligned}\tag{95}$$

For instance, we have the relation $a-in_2$, $b-out_2$, $c-out_1$, $d-in_1$ in $S_3$. Then Eq. (19) can be written as

$$S_3:\quad \begin{pmatrix} out_1\\ in_1\end{pmatrix}=\begin{pmatrix} \cos\theta & -i\sin\theta\\ -i\sin\theta & \cos\theta\end{pmatrix}\begin{pmatrix} in_2\\ out_2\end{pmatrix}$$

$$\begin{aligned}
&\Rightarrow out_2=\sec\theta\, in_1+i\tan\theta\, in_2\\
&\Rightarrow out_1=\cos\theta\, in_2-i\sin\theta(\sec\theta\, in_1+i\tan\theta\, in_2)\\
&\qquad\quad\;=-i\tan\theta\, in_1+\sec\theta\, in_2,
\end{aligned}$$

which is the third equation of Eq. (95). Other equations in Eq. (95) can be derived similarly.

Combining the scattering and the random propagation phases, we obtain the transfer matrices between different layers. For instance, the transfer matrix from $(\eta,0)$ to $(\eta,1)$ in Supplementary Fig. 32(a) is

$$T_{(\eta,1),(\eta,0)}=\begin{array}{r} \\ (1,0)\\ \vdots\\ (\xi-1,1)\\ (\xi,0)\\ (\xi,1)\\ (\xi+1,0)\\ \vdots\\ (M,1)\end{array}\begin{array}{c}\begin{array}{cccccccc}(1,0) & \cdots\cdots & (\xi-1,1) & (\xi,0) & (\xi,1) & (\xi+1,0) & \cdots\cdots & (L,1)\end{array}\\ \left(\begin{array}{cccccccc}
-i\sin\theta & & & & & & & \cos\theta\\
 & \ddots & & & & & & \\
 & & -i\sin\theta & \cos\theta & & & & \\
 & & \cos\theta & -i\sin\theta & & & & \\
 & & & & i\sin\theta & \cos\theta & & \\
 & & & & \cos\theta & i\sin\theta & & \\
 & & & & & & \ddots & \\
\cos\theta & & & & & & & -i\sin\theta
\end{array}\right)\end{array}\times\mathrm{diag}\left[e^{i\vartheta(\eta,0,\xi,\mu)}\right].\tag{96}$$

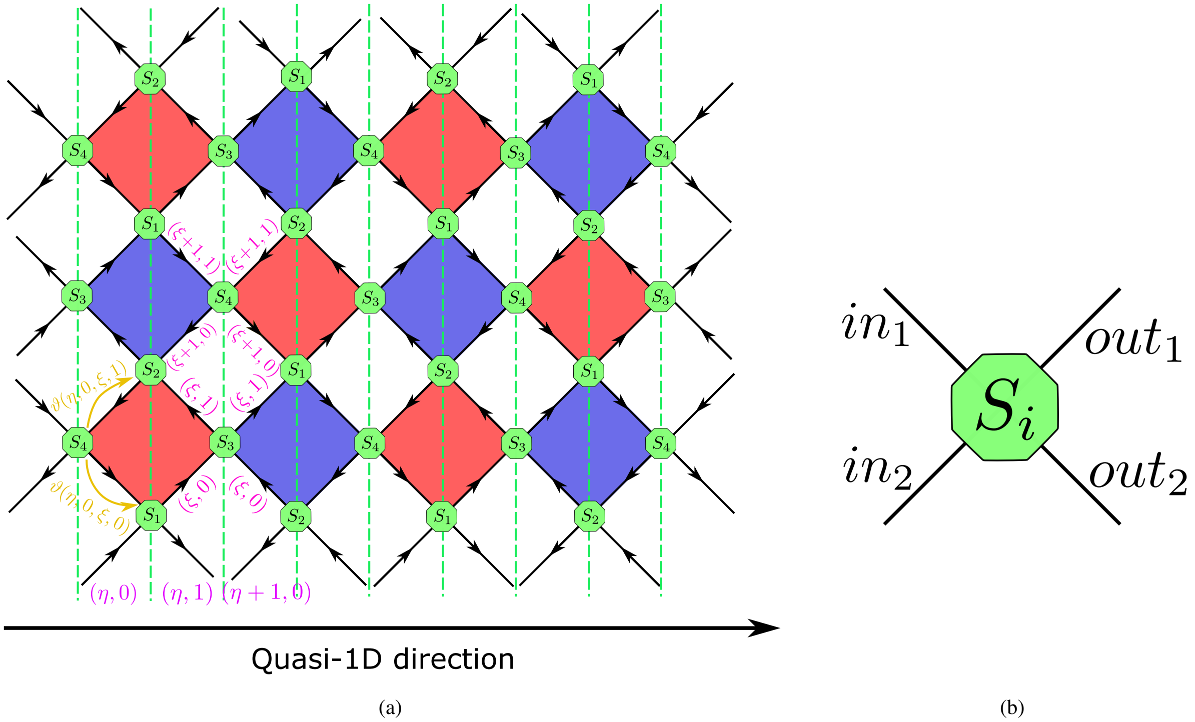


Supplementary Fig. 32. Layer division and scattering nodes of the network model (Eq. (21) & (22)). (a) Layer division of the network model Chiral edges between two adjacent green dashed lines belong to one layer. Purple indices $\{(\mathcal{Y},\nu)|\mathcal{Y}\in\mathbb{Z},\nu=0,1\}$ in the bottom are layer indices. Purple indices $\{(\mathcal{X},\chi)|\mathcal{X}\in\mathbb{Z},\chi=0,1\}$ along square edges denote different edges in one layer. The golden arrows indicate the directions of accumulating random phases $\vartheta(\mathcal{X},\chi,\mathcal{Y},\nu)$. (b) Scattering node $S_i$, $(i=1,2,3,4)$ relating chiral edges in adjacent layers. $in/out_{1,2}$ indicate scattering channels. The scattering effect controlled by $\theta$ can be found in Eq. (95).

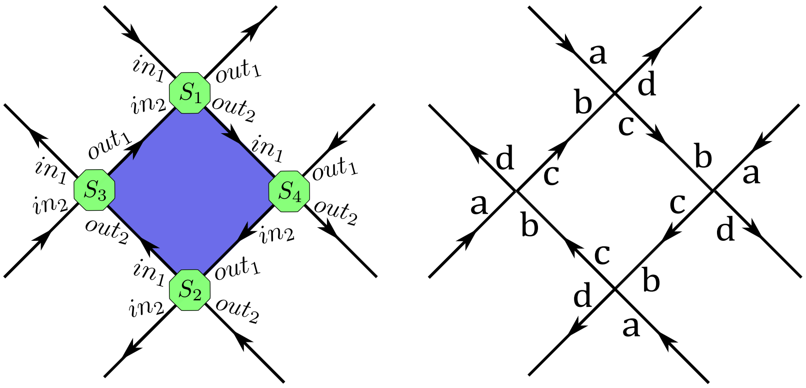


Supplementary Fig. 33. Scattering channel correspondence between Supplementary Fig. 32(b) and Supplementary Fig. 16(b). The left side is a patch of Supplementary Fig. 32(a) containing four kinds of scattering nodes. Correspondence between chiral edges and the $in/out$ channels is illustrated on the left side. And the correspondence between chiral edges and $a\sim d$ channels in Supplementary Fig. 16(b) is illustrated on the right side. Hence we can see the correspondence between the $in/out$ channels and the $a\sim d$ channels for $S_1\cdots S_4$.

and the transfer matrix from $(\eta,1)$ to $(\eta+1,0)$ is

$$T_{(\eta+1,0),(\eta,1)} = \begin{array}{c} \vdots \\ (\xi,0) \\ (\xi,1) \\ (\xi+1,0) \\ (\xi+1,1) \\ \vdots \end{array} \overset{\begin{array}{cccc} \ldots\ldots(\xi,0) & (\xi,1) & (\xi+1,0) & (\xi+1,1)\ldots\ldots \end{array}}{\begin{pmatrix} \ddots & & & & & \\ & i\tan\theta & \sec\theta & & & \\ & \sec\theta & -i\tan\theta & & & \\ & & & -i\tan\theta & \sec\theta & \\ & & & \sec\theta & i\tan\theta & \\ & & & & & \ddots \end{pmatrix}} \times \text{diag}\left[e^{i\vartheta(\eta,1,\xi,\mu)}\right], \tag{97}$$

The diagonal matrix "diag $[\cdot]$" represents the random propagation phases. The transversal size $L$ in Eq. (96) is defined as the total number of squares per layer. In addition, from the scattering part of $T_{(\eta,1),(\eta,0)}$, one can see that we take the periodic boundary condition (PBC). Hence, $L$ has to be even to make the PBC well-defined. The longitudinal length $M$ is defined as the number of layers, *i.e.*, $\eta = 1, 2, \cdots, M/2$.

Transfer matrices $T_{(\eta+1,1),(\eta+1,0)}, T_{(\eta+2,0),(\eta+1,1)}$ can be derived in a similar way, one just needs to exchange the scattering effects in Eq. (95) by $S_1 \leftrightarrow S_2$ & $S_3 \leftrightarrow S_4$ and use the corresponding random phases. The transfer matrices for other layers, apart from the random phase part, must be the same to one of $T_{(\eta,1),(\eta,0)}, T_{(\eta+1,0),(\eta,1)}, T_{(\eta+1,1),(\eta+1,0)}, T_{(\eta+2,0),(\eta+1,1)}$. By successively multiplying these transfer matrices and taking the statistical procedure described in Sec. V A, we can obtain the $\rho_{\text{q}-1\text{D}}$ of the network model. The numerical results are shown in Sec. I A.

With the help of transfer matrix, we can also calculate the conductivity $\sigma$ of the network model [18]. Consider a general 1D scattering process on a sample, the amplitudes of incoming & outgoing channels on the left & right sides (see Supplementary Fig. 34) satisfies

$$\begin{pmatrix} out_R \\ out_L \end{pmatrix} = \begin{pmatrix} t & r' \\ r & t' \end{pmatrix} \begin{pmatrix} in_L \\ in_R \end{pmatrix} \tag{98}$$

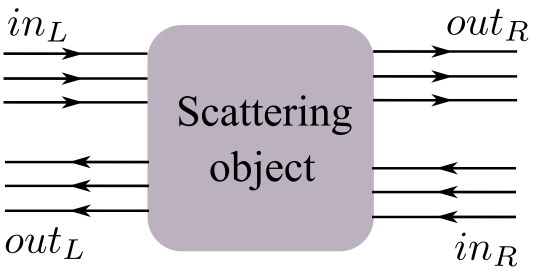


Supplementary Fig. 34. 1D scattering process on a sample. $in_L$ & $out_L$ ($in_R$ & $out_R$) represent the incoming & outgoing channels on the left (right) side, respectively. The arrows indicate the propagation directions of these channels.

According to the Landauer formula, the conductance of the sample is determined by the transmission coefficients

$$G = \frac{e^2}{h}\text{Tr}[t^\dagger t] = \frac{e^2}{h}\text{Tr}[t'^\dagger t']. \tag{99}$$

Thus, we need $t$ or $t'$ to calculate the conductance. Eq. (98) transforms the incoming channels to outgoing channels, while the transfer matrix transform the amplitudes from left to right. Hence, in order to obtain $t$ or $t'$, we should reshape Eq. (98) into

$$\begin{pmatrix} out_R \\ in_R \end{pmatrix} = \begin{pmatrix} t - r't'^{-1}r & r't'^{-1} \\ -t'^{-1}r & t'^{-1} \end{pmatrix} \begin{pmatrix} in_L \\ out_L \end{pmatrix} = V_R^\dagger O_M V_L \begin{pmatrix} in_L \\ out_L \end{pmatrix} = V_R^\dagger \prod_{\eta=1}^{M/2} T_{(\eta+1,0),(\eta,1)} T_{(\eta,1),(\eta,0)} V_L \begin{pmatrix} in_L \\ out_L \end{pmatrix}, \tag{100}$$

where $O_M = \prod_{n=1}^{M} T_n$ (defined in Supplementary Sec. V A) is a product of $M$ consecutive transfer matrices. Since the bases of transfer matrices may not coincide with the $in/out_{L,R}$ channels, unitary matrices $V_L$ & $V_R$ are introduced to transform $(in_L, out_L)^T$ & $(out_R, in_R)^T$ into the bases of $T_1$ & $T_M$, respectively. (We will see a concrete construction on the network model later.) In the third equation of Eq. (100), we apply the general formula to our network model.

The remaining part of this subsection could be too technical for most readers. Thus, in this paragraph, we summarize the main idea for readers that is not interested in the details. In Eq. (96) & (97), we have already chosen a basis (an index of chiral edges

in one layer) for the transfer matrix. We denote this basis as $\mathcal{I}_1$. However, $\mathcal{I}_1$ is designed by the spatial positions of chiral edges, not by their correspondence with $in$ & $out$ channels. Hence, we need to identify the correspondence between chiral edges and $in$ & $out$ channels, and then design a new basis $\mathcal{I}_2$ to index these channels. Finally, we use $V_L$, $V_R$ to translate $\mathcal{I}_2$ into $\mathcal{I}_1$ and show how to obtain $t'$. Now we turn to the explicit construction.

The concrete form of channels depends on the setting of leads. For convenience, we take the clean Manhattan networks as leads, *i.e.*, we add several clean layers to two terminals of the disordered sample (see Supplementary Fig. 35). Now, we try to identify the incoming and outgoing channels in terms of the notation $(\mathcal{X}, \chi, \mathcal{Y}, \nu)$ defined at the beginning of this subsection. We take $\mathcal{X} = 1 \,\&\, \chi = 0$ as the first layer inside the sample and $\mathcal{X} = M/2 \,\&\, \chi = 1$ as the last layer. The left and right leads contain layers $\{(\mathcal{X}, \chi)|\mathcal{X} \leq 0, \chi = 0, 1\}$ and $\{(\mathcal{X}, \chi)|\mathcal{X} > \frac{M}{2}, \chi = 0, 1\}$, respectively. Recall that $\{(\mathcal{X}, \mathcal{Y})|(-1)^{\mathcal{X}+\mathcal{Y}} = 1\}$ $\left(\{(\mathcal{X}, \mathcal{Y})|(-1)^{\mathcal{X}+\mathcal{Y}} = -1\}\right)$ corresponds to the red (blue) squares. Hence, $\xi$ and $\frac{M}{2}$ in Supplementary Fig. 35 are odd numbers. In the left lead, the $in_L$ (right-going) channels end at edges $\{(0, 1, \mathcal{Y}, \nu)|(-1)^{\mathcal{Y}+\nu} = 1\}$, and the $out_L$ (left-going) channels start at edges $\{(0, 1, \mathcal{Y}, \nu)|(-1)^{\mathcal{Y}+\nu} = -1\}$. Similarly, $out_R$ starts at $\{(\frac{M}{2} + 1, 0, \mathcal{Y}, \nu)|(-1)^{\mathcal{Y}+\nu} = 1\}$ and $in_R$ ends at $\{(\frac{M}{2} + 1, 0, \mathcal{Y}, \nu)|(-1)^{\mathcal{Y}+\nu} = -1\}$. If the sample ends up with $\frac{M}{2}$ = even number, the correspondence between $in/out_R$ and the edges in layer $(\frac{M}{2} + 1, 0)$ is reversed.

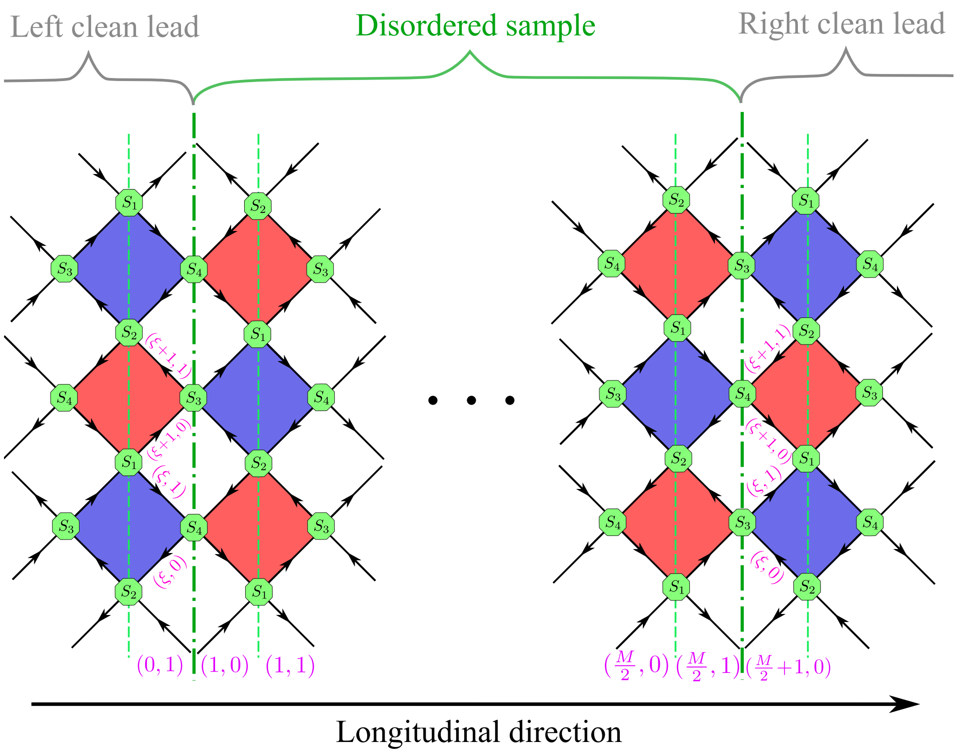


Supplementary Fig. 35. The setting of leads of the network model (Eq. (21) & (22)). The disordered sample contains layers $(1, 0) \sim (M/2, 1)$, the left lead contains $\{(\mathcal{X}, \chi)|\mathcal{X} \leq 0, \chi = 0, 1\}$, and the right lead contains $\{(\mathcal{X}, \chi)|\mathcal{X} > \frac{M}{2}, \chi = 0, 1\}$. In this figure, $(1, \chi, \xi, \nu)$ & $(M/2, \chi, \xi, \nu)$ correspond to red squares. In order to conform the convention $(-1)^{\mathcal{X}+\mathcal{Y}} = 1 \sim$ red square, $M/2$ and $\xi$ are odd numbers. Therefore, in this figure, $in_L$ corresponds to edges $\{(0, 1, \mathcal{Y}, \nu)|(-1)^{\mathcal{Y}+\nu} = 1\}$ and $out_R$ corresponds to $\{(\frac{M}{2} + 1, 0, \mathcal{Y}, \nu)|(-1)^{\mathcal{Y}+\nu} = 1\}$. Other edges in layers $(0, 1)$ and $(\frac{M}{2} + 1, 0)$ correspond to $out_L$ and $in_R$, respectively.

If there are $L$ squares in the transversal direction, $in_L$, $in_R$, $out_L$, $out_R$ all contain $L$ channels. We define the mth $in_L$ channel as the channel ends at edge $(0, 1, m, \text{mod}(m, 2))$. Similarly, the channel starts at $(0, 1, m, \text{mod}(m + 1, 2))$ is defined as the mth $out_L$ channel. When $\frac{M}{2}$ = odd number (as it is in Supplementary Fig. 35), the mth $out_R$ channel starts at $(\frac{M}{2}, 0, m, \text{mod}(m, 2))$ and the mth $in_R$ channel ends at $(\frac{M}{2}, 0, m, \text{mod}(m + 1, 2))$.

Since each lead contains both incoming and outgoing channels, we also need to index these channels together. See Supplementary Fig. 35, there are $2L$ channels in each lead, hence we need to index $in$ & $out$ channels in each lead by integers in $[1, 2L]$. We first label the left channels: the mth $in_L$ & m′th $out_L$ channels are labeled by $m$ & $L + m'$, respectively. Since $in_L$ will transmit to $out_R$, we use the same integer to label the mth $in_L$ and the mth $out_R$ channels. Similarly, we label the m′th $out_L$ and the m′th $in_R$ by the same integer. Hence, we label mth $out_R$ and m′th $in_R$ channels by $m$ and $L + m'$, respectively. We

can denote this label strategy by $\mathcal{I}_2$ and rewrite it in a vector form:

$$\begin{aligned} L_{L+m}^T &= (\overbrace{0\cdots\cdots0}^{in_L},\overbrace{0\cdots\underbrace{1}_{mth}\cdots0}^{out_L})^T \quad m\in[1,L] \\ R_{L+n}^T &= (\overbrace{0\cdots\cdots0}^{out_R},\overbrace{0\cdots\underbrace{1}_{nth}\cdots0}^{in_R})^T \quad n\in[1,L] \end{aligned} \tag{101}$$

The vectors of $in_L$ and $out_R$ are defined in a similar way.

On the other hand, as a basis of the transfer matrix, edges in one layer $\{(\mathcal{Y},\nu)|\mathcal{Y}\in[1,L],\nu=0,1\}$ have already been labeled by $\{2\mathcal{Y}+\nu\}$ (see Eq. (96) & (97)). We denote this label manner as $\mathcal{I}_1$. Therefore, we need $V_L$ & $V_R$ to relate $\mathcal{I}_1$ and $\mathcal{I}_2$. $V_L$ is a $2L\times2L$ orthogonal matrix that transforms $in_L\,\&\,out_L$ channels indexed by $\mathcal{I}_2$ to edges indexed by $\mathcal{I}_1$ on the left side, *i.e.*, layer $(0,1)$. Similarly, $V_R$ transforms $in_R\,\&\,out_R$ to the edges on right side, *i.e.*, layer $(\frac{M}{2}+1,0)$. We denote the entry of $V_{L,R}$ by $V_{L,R}[\text{row index};\ \text{column index}]$. Hence, the nonzero entries of $V_{L,R}$ have the form: $V_{L,R}[\mathcal{I}_1\,\text{index of some channel},\mathcal{I}_2\,\text{index of the same channel}]=1$. It can be checked that the nonzero entries explicitly are

$$\{V_{L,R}[2m+\text{mod}(m,2);m]=V_{L,R}[2m+\text{mod}(m+1,2);m+L]=1\,|\,m\in[1,L]\}\,. \tag{102}$$

According to Eq. (100), we can obtain $t'$ by calculating the amplitudes of $\{\text{nth}\,in_R\to\text{mth}\,out_L|n,m\in[1,L]\}$:

$$(t'^{-1})_{nm}=R_{L+n}^\dagger V_R^\dagger\prod_{\eta=1}^{M/2}T_{(\eta+1,0),(\eta,1)}T_{(\eta,1),(\eta,0)}V_L L_{L+m}, \tag{103}$$

where $L_{L+m}$, $R_{L+n}$ are defined in Eq. (101). After taking inverse of Eq. (103), we can calculate the conductance (and hence the conductivity) by Eq. (99) and the results are shown in Supplementary Sec. I A.

To end the discussion of conductivity, we should emphasize that the conductivity is not calculated on a quasi-1D sample. The calculation of $\rho_{\text{q}-1\text{D}}$ needs a precise evaluation of the Lyapunov exponents, so we need to multiply a large number of random matrices together and utilize the self-average effect to do statistical analysis (the strategy described in Supplementary Sec. V A). However, conductivity does not explicitly rely on the Lyapunov exponents. Therefore, such a statistical analysis (on segments of a quasi-1D sample) is not necessary. In addition, this strategy has two drawbacks that prevent us from using it. First, what we can directly calculate is the conductance $G=\sigma L/M$. In a quasi-1D sample ($M\gg L$), the error of conductance $\Delta G$ will be amplified by the large factor $\frac{M}{L}$ and result in a considerable error in conductivity $\Delta\sigma=\frac{M}{L}\Delta G$. Second, the $\sigma$ derived from a quasi-1D sample may intrinsically differ from that of a normal shaped 2D sample. Thus, we instead take the statistical analysis on a large number (usually $10^4\sim10^5$) of normal shaped samples with different disorder configurations.

### C. Transfer matrices of eight-band lattice models $H_{8B}$ and $H'_{8B}$

Before moving to our lattice models $H_{8B}$ (Eq. (66)) and $H'_{8B}$ (Eq. (85)), we derive the transfer matrices in a general lattice model whose Hamiltonian is

$$H=\sum_{ij}t_{ij}c_i^\dagger c_j=\sum_{q=-r_1}^{r_2}\sum_{l=1}^{M}\sum_{\alpha,\beta=1}^{s}t_{l\alpha,(l+q)\beta}\,c_{l\alpha}^\dagger c_{(l+q)\beta} \tag{104}$$

where $i$, $j$ are site indices and $t_{i,j}$ is the hopping coefficient from $j$ to $i$. The communication relation is $\{c_i,c_j^\dagger\}=\delta_{i,j}$. In the second equation, we decompose site indices into layer indices ($l$ & $l+q$) and indices inside one layer ($\alpha\,\&\,\beta$). $M$ is the total number of layers and $s$ is the total number of degrees of freedom per layer. The restriction on the summation over $q$, *i.e.*, $q\in[-r_1,r_2]$ means that only the $(l-r_1)$th $\sim$ $(l+r_2)$th layers can hop to the $l$ th layer (in one step). This restriction does not significantly damage the generality of Eq. (104) since it merely forbids the hopping to be infinite-long range in the longitudinal direction.

The eigenstates of $H$ should satisfy the stationary equation $E|\psi\rangle=H|\psi\rangle$ and we extract one component:

$$E\psi_{l\alpha}=\sum_{q=-r_1}^{r_2}\sum_{\beta}t_{l\alpha,(l+q)\beta}\psi_{(l+q)\beta}$$

where $\psi_{l\alpha}$ is the amplitude on site $(l\alpha)$, *i.e.*, $|\psi\rangle = \sum_{l,\alpha}\psi_{l\alpha}c^\dagger_{l\alpha}|0\rangle$. We can combine amplitudes in one layer into a vector $\vec{\psi}_l = (\psi_{l1},\psi_{l2},...\psi_{ls})^T$ and define the Hamiltonian block $(H_{l,l'})_{\alpha\beta} = \langle 0|c_{l,\alpha}Hc^\dagger_{l',\beta}|0\rangle = t_{l\alpha,l'\beta}$. Therefore

$$\begin{aligned} E\vec{\psi}_l &= \sum_{q=-r_1}^{r_2} H_{l,l+q}\vec{\psi}_{l+q} \\ \Longrightarrow H_{l,l+r_2}\vec{\psi}_{l+r_2} &= -\left(\sum_{q=1}^{r_2-1}+\sum_{q=-r_1}^{-1}\right)H_{l,l+q}\vec{\psi}_{l+q} - (H_{l,l}-E)\,\vec{\psi}_l \end{aligned} \tag{105}$$

If $H_{l,l+r_2}$ is invertible, we can reformulate the second equation as

$$\begin{bmatrix} \vec{\psi}_{l+r_2} \\ \hline \vec{\psi}_{l+r_2-1} \\ \vdots \\ \vdots \\ \vec{\psi}_{l-r_1+1} \end{bmatrix} = \left[\begin{array}{c|c} -H^{-1}_{l,l+r_2}H_{l,l+r_2-1} \;\cdots\; -H^{-1}_{l,l+r_2}(H_{l,l}-E) \;\cdots\; -H^{-1}_{l,l+r_2}H_{l,l-r_1+1} & -H^{-1}_{l,l+r_2}H_{l,n-r_1} \\ \hline I_{s(r_1+r_2-1)\times s(r_1+r_2-1)} & 0_{s(r_1+r_2-1)\times s} \end{array}\right] \begin{bmatrix} \vec{\psi}_{l+r_2-1} \\ \vdots \\ \vec{\psi}_l \\ \vdots \\ \hline \vec{\psi}_{l-r_1} \end{bmatrix} \tag{106}$$

Eq. (106) relates $\vec{\psi}_{l-r_1} \sim \vec{\psi}_{l+r_2-1}$ with $\vec{\psi}_{l-r_1+1} \sim \vec{\psi}_{l+r_2}$. And we can take the transformation matrix in Eq. (106) as the transfer matrix of $l$th layer ($T_l$).

If different choices of the quasi-1D directions do not demonstrate qualitative different localization behaviors, we are free to choose the quasi-1D direction to optimize the numerical performance. First, we should take the quasi-1D direction that makes $(r_1+r_2)$ as small as possible since the transfer matrix in Eq. (106) is $s(r_1+r_2)$-dimensional, and we want the transversal size $L \propto s$ to be as large as possible. Second, we should avoid a nearly singular $H_{l,l+r_2}$, *i.e.*, $\det(H_{l,l+r_2}) \approx 0$, since Eq. (106) explicitly depends on $H^{-1}_{l,l+r_2}$. If one, unfortunately, encounters a nearly singular $H_{l,l+r_2}$, he/she can try changing the quasi-1D direction or taking a finer layer division to resolve it.

Now, we turn to our lattice models $H_{8B}$ and $H'_{8B}$. In Supplementary Sec. V A, we have argued that different choices of the quasi-1D directions will not make qualitative difference in our models. Therefore, we are free to choose the quasi-1D directions of $H_{8B}$ and $H'_{8B}$ without losing the comparability with the network model. In order to minimize $(r_1+r_2)$, we take a different quasi-1D direction (x direction) in $H_{8B}$ and $H'_{8B}$ from the network model (x+y direction). As shown in Supplementary Fig. 36, sites inside two adjacent pink dashed lines belong to one layer. Such a choice gives $r_1 = r_2 = 1$, no matter whether the square diagonal hopping is removed or not. In other words, we only have hoppings between the nearest neighbor layers for both $H_{8B}$ and $H'_{8B}$. The disorder, as we have explained in Sec. IV F, is restricted to the on-site form $U_{rand}(i)c^\dagger_i c_i$, where the random potentials $U_{rand}(i)$ uniformly distribute in $[-W/2, W/2]$ and are independent to each other.

The concrete formulae of transfer matrices in our lattice models is quite complicated, since every unit cell is divided into four layers. As illustrated in Supplementary Fig. 36, the layer index has three components $(n,\alpha,\beta)$ and intra-layer index has two components $(m,\gamma)$, where $(n,m)$ and $\alpha$ are previous cell and chiral indices, respectively. Two new indices $\beta,\gamma = +,-$ together play the same role as the corner index $w$ defined in Eq. (64). The reason for splitting $w$ into $\beta$ and $\gamma$ is to fit the transfer matrix formula, *i.e.*, indicate the degrees of freedom by (layer indices)+(intra-layer indices). The relation between them is $w = 5/2 - \beta(1+\gamma/2)$, which can be directly checked by comparing Supplementary Fig. 36 with Supplementary Fig. 24.

Since we only have hoppings between nearest neighbor layers, we can denote the non-zero Hamiltonian blocks as $h_{l,\pm} = H_{l,l\pm1}$, $h_{l,0} = H_{l,l}$. Explicit expressions of these blocks are

$$\begin{aligned} \left(h_{(n,\alpha,\beta),0}\right)_{m\gamma,m\gamma} &= U_{rand}(n,m,\alpha,\beta,\gamma) \qquad \alpha,\beta,\gamma = \pm 1 \\ \left(h_{(n,\alpha,\beta),0}\right)_{m\gamma,m\bar{\gamma}} &= (-\alpha\beta\gamma A i - 1)\frac{\pi v}{4a} \\ \left(h_{(n,\alpha,\beta),\overline{\alpha\beta}}\right)_{m\gamma,m\gamma} &= (\alpha\beta\gamma A i - 1)\frac{\pi v}{4a} \\ \left(h_{(n,\alpha,\beta),\overline{\alpha\beta}}\right)_{m\gamma,m\bar{\gamma}} &= t' \\ \left(h_{(n,\alpha,\beta),\alpha\beta}\right)_{m+,m+} &= \left(h_{(n,\alpha,\beta),\alpha\beta}\right)_{m-,(m+\alpha)-} = \tilde{t} \end{aligned} \tag{107}$$

Other elements in $h_{(n,\alpha,\beta),0}$ and $h_{(n,\alpha,\beta),\pm}$ are zero.

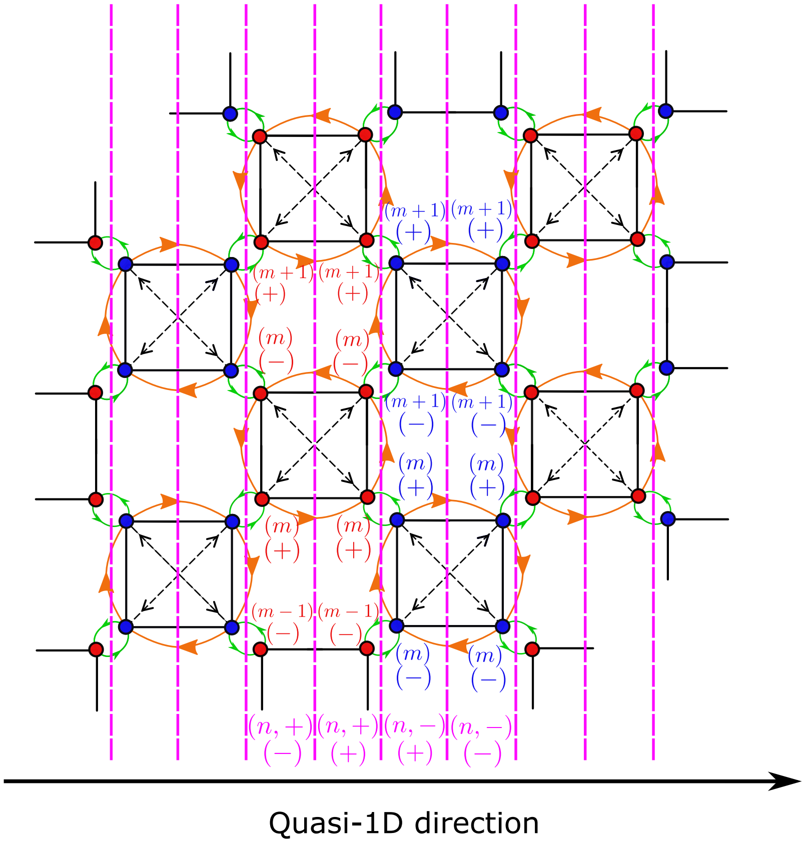


Supplementary Fig. 36. Layer division of $H_{8B}$ defined in Eq. (66). For $H'_{8B}$ defined in Eq. (85), the division is the same but without square diagonal hopping. Corners inside two adjacent pink dashed lines belong to one layer. The pink indices $\{(n, \alpha, \beta) | n \in \mathbb{Z}, \alpha, \beta = \pm\}$ in the bottom are layer indices. The red and blue indices $\{(m, \gamma) | m \in \mathbb{Z}, \gamma = \pm\}$ further distinguish different corners in one layer. Among these indices, $(n, m)$ and $\alpha$ are previous cell index and chiral index, respectively. Two new indices $\beta$ & $\gamma$ together play the same role as the corner index $w$. The relation between them is $w = \frac{5}{2} - \beta(1 + \frac{\gamma}{2})$.

Here, $\overline{(\cdots)}$ means $(-1) \times (\cdots)$, $t'$ & $\tilde{t}$ are square diagonal hopping & inter-square hopping, and $A$ is the coefficient controlling $DOS_F$ defined in Eq. (86). For $H_{8B}$, $A = 1, t' = -\frac{\pi v}{4a}$ (Eq. (66)) and for $H'_{8B}$, $A > 0, t' = 0$ (Eq. (85)). The transversal size $L$ is defined by the number of cells involved in one layer, *i.e.*, $m = 1, 2, \cdots, L$. We use the periodic boundary condition in the transversal direction. The definition of longitudinal size $M$ is the number of layers, *i.e.*, $n = 1, 2, \cdots, M/4$. We choose $A = 1.2$ in our calculations and exhibit the results in Supplementary Sec. I B & I C.

If one finds Eq. (107) confusing, one can span it with explicit $\alpha, \beta$ indices. For example, for $\alpha = +, \beta = +$, the nonzero matrix elements are given by

$$
\begin{aligned}
\left(h_{(n,+,+),0}\right)_{m+,m-} &= \left(h_{(n,+,+),0}\right)^{*}_{m-,m+} = (-Ai-1)\frac{\pi v}{4a} \\
\left(h_{(n,+,+),-}\right)_{m+,m+} &= \left(h_{(n,+,+),-}\right)^{*}_{m-,m-} = (Ai-1)\frac{\pi v}{4a} \\
\left(h_{(n,+,+),-}\right)_{m+,m-} &= \left(h_{(n,+,+),-}\right)_{m-,m+} = t' \\
\left(h_{(n,+,+),+}\right)_{m+,m+} &= \left(h_{(n,+,+),+}\right)_{m-,(m+1)-} = \tilde{t},
\end{aligned}
.
$$

**Supplementary References**


[1] Howard J. Seltman, *Experimental Design and Analysis* (2008).
[2] Tilo Strutz, *Data fitting and uncertainty: A practical introduction to weighted least squares and beyond*, Vol. 1 (Springer, 2011).
[3] Terry A Loring and Matthew B Hastings, "Disordered topological insulators via c*-algebras," EPL (Europhysics Letters) **92**, 67004 (2011).
[4] Emil Prodan, Taylor L. Hughes, and B. Andrei Bernevig, "Entanglement spectrum of a disordered topological chern insulator," Phys. Rev. Lett. **105**, 115501 (2010).
[5] Raffaello Bianco and Raffaele Resta, "Mapping topological order in coordinate space," Phys. Rev. B **84**, 241106 (2011).
[6] Zhi-Da Song, Luis Elcoro, and B. Andrei Bernevig, "Twisted bulk-boundary correspondence of fragile topology," Science **367**, 794–797 (2020).
[7] Luis Elcoro, Benjamin J. Wieder, Zhida Song, Yuanfeng Xu, Barry Bradlyn, and B. Andrei Bernevig, "Magnetic topological quantum chemistry," Nature Communications **12**, 5965 (2021), number: 1 Publisher: Nature Publishing Group.
[8] Jonah Herzog-Arbeitman, Zhi-Da Song, Luis Elcoro, and B Andrei Bernevig, "Hofstadter topology with real space invariants and reentrant projective symmetries," arXiv preprint arXiv:2209.10559 (2022).
[9] S. V. Gallego, E. S. Tasci, G. de la Flor, J. M. Perez-Mato, and M. I. Aroyo, "Magnetic symmetry in the Bilbao Crystallographic Server: a computer program to provide systematic absences of magnetic neutron diffraction," Journal of Applied Crystallography **45**, 1236–1247 (2012), number: 6 Publisher: International Union of Crystallography.
[10] Junyeong Ahn, Sungjoon Park, and Bohm-Jung Yang, "Failure of nielsen-ninomiya theorem and fragile topology in two-dimensional systems with space-time inversion symmetry: Application to twisted bilayer graphene at magic angle," Physical Review X **9**, 021013 (2019).
[11] Chen Fang, Yige Chen, Hae-Young Kee, and Liang Fu, "Topological nodal line semimetals with and without spin-orbital coupling," Physical Review B **92**, 081201 (2015).
[12] QuanSheng Wu, Alexey A. Soluyanov, and Tomáš Bzdušek, "Non-abelian band topology in noninteracting metals," Science **365**, 1273–1277 (2019).
[13] Adrien Bouhon, QuanSheng Wu, Robert-Jan Slager, Hongming Weng, Oleg V Yazyev, and Tomáš Bzdušek, "Non-abelian reciprocal braiding of weyl points and its manifestation in zrte," Nature Physics **16**, 1137–1143 (2020).
[14] X. C. Xie, X. R. Wang, and D. Z. Liu, "Kosterlitz-Thouless-Type Metal-Insulator Transition of a 2D Electron Gas in a Random Magnetic Field," Physical Review Letters **80**, 3563–3566 (1998), publisher: American Physical Society.
[15] Viktor Z. Cerovski, "Critical exponent of the random flux model on an infinite two-dimensional square lattice and anomalous critical states," Physical Review B **64**, 161101 (2001).
[16] Gang Xiong, Shi-Dong Wang, Qian Niu, De-Cheng Tian, and X. R. Wang, "Metallic Phase in Quantum Hall Systems due to Inter-Landau-Band Mixing," Physical Review Letters **87**, 216802 (2001).
[17] J L Pichard and G Sarma, "Finite size scaling approach to anderson localisation," Journal of Physics C: Solid State Physics **14**, L127–L132 (1981).
[18] P. W. Anderson, D. J. Thouless, E. Abrahams, and D. S. Fisher, "New method for a scaling theory of localization," Phys. Rev. B **22**, 3519–3526 (1980).
